# Value chain interventions for improving women's economic empowerment: A mixed‐methods systematic review and meta‐analysis: A systematic review

**DOI:** 10.1002/cl2.1428

**Published:** 2024-08-12

**Authors:** Suchi Kapoor Malhotra, Swati Mantri, Neha Gupta, Ratika Bhandari, Ralph Nii Armah, Hamdiyah Alhassan, Sarah Young, Howard White, Ranjitha Puskur, Hugh Sharma Waddington, Edoardo Masset

**Affiliations:** ^1^ Campbell South Asia Vasant Kunj Delhi India; ^2^ Global Development Network Vasant Kunj New Delhi India; ^3^ Institute of Statistical, Social and Economic Research University of Ghana Accra Ghana; ^4^ Department of Applied Economics University for Development Studies Tamale Ghana; ^5^ Research and Academic Services Carnegie Mellon University Pittsburgh Pennsylvania USA; ^6^ Campbell Collaboration, Global Development Network Lanzhou University Lanzhou China; ^7^ Sustainable Impact Platform, International Rice Research Institute Los Baños Philippines; ^8^ London School of Hygiene and Tropical Medicine London UK; ^9^ London School of Hygiene and Tropical Medicine, Centre of Excellence in Development Impact and Learning (CEDIL) London UK

**Keywords:** mixed‐method evidence synthesis, self help groups, smallholder farmers, value chain, value chain interventions, women's coperatives and fair trade, women's economic empowerment

## Abstract

**Background:**

Value chain interventions have become widespread throughout the international development sector over the last 20 years, and there is a need to evaluate their effectiveness in improving women's welfare across multiple dimensions. Agricultural value chains are influenced by socio‐cultural norms and gender dynamics that have an impact on the distribution of resources, benefits, and access to opportunities. While women play a critical role in agriculture, they are generally confined to the least‐valued parts of the value chain with the lowest economic returns, depending on the local, social and institutional contexts.

**Objectives:**

The review assesses the effectiveness of approaches, strategies and interventions focused on women's engagement in agricultural value chains that lead to women's economic empowerment in low‐ and middle‐income countries. It explores the contextual barriers and facilitators that determine women's participation in value chains and ultimately impact their effectiveness.

**Search Methods:**

We searched completed and on‐going studies from Scopus, Web of Science Core Collection (Social Sciences Citation Index [SSCI], Science Citation Index Expanded [SCI‐EXPANDED], Conference Proceedings Citation Index – Science [CPCI‐S], Conference Proceedings Citation Index – Social Science & Humanities [CPCI‐SSH], and Emerging Sources Citation Index [ESCI]), International Bibliography of the Social Sciences, EconLit, Business Source Premier, APA PsycInfo, Cochrane Central Register of Controlled Trials, Cochrane, Database of Systematic Reviews, CAB Abstracts and Sociological Abstracts. We also searched relevant websites such as Consortium of International Agricultural Research Centers (CGIAR); the International Fund for Agricultural Development (IFAD); AgriProFocus; the Bill & Melinda Gates Foundation (BMGF); Donor Committee for Enterprise Development; the UN Food and Agriculture Organisation (FAO); the International Labour Organisation (ILO); the Netherlands Development Organisation; USAID; the Swiss Agency for Development and Cooperation; the International Food Policy Research Institute; World Agroforestry; the International Livestock Research Institute; the Foreign, Commonwealth & Development Office; the British Library for Development Studies (BLDS); AGRIS; the IMMANA grant database; the 3ie impact evaluation database; Innovations for Poverty Action (IPA); The Abdul Latif Jameel Poverty Action Lab (J‐PAL); the World Bank IEG evaluations; the USAID Development Data Library; Experience Clearinghouse; the proceedings of the Agriculture, Nutrition and Health Academy conference; the proceedings of the Centre for the Study of African Economies (CSAE) Conference; the proceedings of the North East Universities Development Consortium (NEUDC) Conference; and the World Bank Economic Review. The database search was conducted in March 2022, and the website search was completed in August 2022.

**Selection Criteria:**

The review includes value chain interventions evaluating the economic empowerment outcomes. The review includes effectiveness studies (experimental and non‐experimental studies with a comparison group) and process evaluations.

**Data Collection and Analysis:**

Two review authors independently assessed studies for inclusion, extracted data, critically appraised the studies, and synthesised findings.

**Results:**

We found that value chain interventions are successful in improving the economic conditions of their intended beneficiaries. The interventions were found to improve women's economic outcomes such as income, assets holdings, productivity, and savings, but these effects were small in size and limited by low confidence in methodological quality. The meta‐analysis suggests that this occurs more via the acquisition of skills and improved inputs, rather than through improvement in access to profitable markets. The qualitative evidence on interventions points to the persistence of cultural barriers and other constraints. Those interventions implemented in Sub‐Saharan Africa and South Asia are consistently more successful for all outcomes considered, although there are few studies conducted in other areas of the world.

**Conclusions:**

The review concludes that value chain interventions empower women, but perhaps to a lesser extent than expected. Economic empowerment does not immediately translate into empowerment within families and communities. Interventions should either moderate their expectations of empowerment goals, or they should be implemented in a way that ensures higher rates of participation among women and the acquisition of greater decision‐making power.

## 1 PLAIN LANGUAGE SUMMARY

Value chain interventions for improving women's economic empowerment in low‐ and middle‐income countries have small positive effects on women's mobility, decision‐making, and leadership of organisations but low confidence due to methodological limitations.

### 1.1 The review in brief

This review evaluates the effectiveness of value chain interventions in improving women's welfare. The review indicates that these interventions are successful in improving economic conditions for their beneficiaries; through acquisition of skills and improved inputs, rather than through improved access to markets. This review found small positive effects on women's mobility, decision‐making, and leadership of organisations but with low confidence due to methodological limitations. Qualitative findings point to the persistence of cultural barriers and other constraints.

### 1.2 What is the review about?

While women play a critical role in agriculture, they are confined to the least‐valued sections of the value chain that have the lowest economic returns – this is influenced by the local, social and institutional contexts. Women have restricted access to execute their roles in value chains while at the same time they are also burdened with a higher expectation to deliver.

Value chain is conceptualised as the understanding of agricultural product markets and identifying unexploited non‐farm opportunities.

### 1.3 What is the aim of this review?

The review aims to evaluate effectiveness of approaches, strategies and interventions focused on women's engagement in the agricultural value chains that lead to their economic empowerment in low‐ and middle‐income countries. The review summarises evidence from 118 studies: 4 mixed methods, 59 effectiveness and 55 process evaluations.

### 1.4 What are the main findings of the review?

The review finds that value chain interventions are successful in modestly improving women's economic outcomes – income, assets holdings, productivity, and savings. The meta‐analysis suggests that this occurs more through acquisition of skills and improved inputs, over improvement in access to profitable markets. These findings are limited by low confidence in methodological quality of the studies.

The quantitative analysis suggests that the interventions' success in improving women's economic outcomes does not always translate into an increase in non‐economic empowerment. The quantitative effects on women's mobility, decision‐making, and leadership of organisations are positive, but limited in size. This suggests that although the interventions are able to change economic roles within value chains, these changes are not sufficiently strong to significantly alter gender roles within families and communities. Qualitative evidence displays persistence of cultural barriers and other constraints.

The interventions implemented in Sub‐Saharan Africa and South Asia are consistently more successful for all outcomes considered. However, few studies have been conducted in other areas of the world, therefore, the estimates from this area lack precision and statistical power.

Value chain interventions specifically targeted to women do not produce significantly larger economic impacts than non‐targeted interventions. However, they produce slightly larger impacts on indicators of time use and non‐economic empowerment. The qualitative findings identified facilitators to women's empowerment which included capacity building, network formation, market access and land ownership as well as barriers such as exploitation, lack of information, stereotypes and lack of control over resources. Based on the qualitative findings, it is suggested that interventions promoting horizontal integration through farmer‐based organisations, self‐help groups, and cooperatives are important facilitators for promoting women's participation, including leadership, decision‐making, and access to the economic benefits of the interventions.

### 1.5 What do the findings of this review mean?

The review concludes that value chain interventions empower women, but perhaps to a lesser extent than expected. Economic empowerment does not immediately translate into empowerment within families and communities. Interventions should either moderate their expectations on empowerment goals, or they should be implemented in a way that ensures higher rates of participation among women and the acquisition of greater decision‐making power.

This review is unable to conclude whether some interventions are more successful than others or to point to intervention mechanisms that are more likely to improve empowerment indicators to a sufficient level of confidence. However, qualitative and quantitative evidence included in the review suggest that untargeted value chain interventions are unlikely to produce the same impact on women's empowerment as interventions targeted at women.

## 2 BACKGROUND

### 2.1 The issue

Agricultural value chains are influenced by socio‐cultural norms and gender dynamics that impact distribution of resources, benefits, and access to opportunities (Rubin et al., 2010). While women play a critical role in agriculture, they are generally confined to areas of the value chain with the fewest or lowest economic returns, as per the social and institutional contexts.

Several development initiatives (both governmental and bilateral) and results for development programmes have prioritised investment in attaining women's economic empowerment; they accorded these goals via women's participation in agricultural value chains or food systems (as entrepreneurs) and through stronger market engagement. The UN secretary general's panel on women's economic empowerment emphasises the importance of ‘supporting and enabling women to reach their full potential at all levels of the value chain’ (Pyburn & van Eerdewijk, 2021).

There are multiple definitions of economic empowerment. One of the most accepted was advanced by Eyben and colleagues (2008) as ‘the capacity of poor women and men to participate in, contribute to and benefit from growth processes in ways that recognise the value of their contribution, respect their dignity, and make it possible to negotiate a fairer distribution of the benefits of growth’. Another definition of relevance is by Golla and colleagues (2011), who emphasised that ‘a woman is economically empowered when she has both the ability to succeed and advance economically and the power to make and act on economic decisions’.

Research by Kabeer and Natali (2013) found that although women's entry into the labour market contributes significantly to productivity gains and economic growth, the outcomes of economic growth do not essentially result in gender equality or lead to recognition of power relations constructs. Gender dynamics within value chains operate from individual interactions at the household level up to the level of the value chain, and that of participation‐related issues versus factors that govern levels of gains from participation.

Women have restricted access to resources and information, less control over assets, and heavier workloads, thereby constraining their capacity to engage with and operate within the higher nodes of the value chain (i.e., processing and trading, which often require a minimum number of resources and training) (Forsythe et al., 2016; Meinzen‐Dick et al., 2011). Agricultural value chain interventions aim to create equitable participation in agricultural markets among men and women by enabling women to overcome the above‐mentioned barriers and facilitating their participation in markets. These efforts support women's economic advancement to realise their entrepreneurial aspirations.

#### 2.1.1 Value chains

Traditionally, agricultural interventions have tried to improve farmers' incomes by increasing farm productivity. This has been pursued, for example, through the introduction of new farming technologies and agricultural extension, and by providing access to inputs such as fertiliser, seeds, and loans. However, developments in global market integration over the last 30–40 years suggest that farmers will have a chance to access better income opportunities by strengthening their positions within agricultural product markets. For example, farmers may gain considerably by supplying their goods to a large retailer located in a high‐income country, or they may obtain better prices after storing and processing their produce.

The ‘value chain’ is a conceptual framework for understanding agricultural product markets and identifying unexploited non‐farm opportunities. Although the concept of a value chain is multivariate in how it is used, for example, by different UN agencies (Stamm & Von Drachenfels, 2011); it is primarily used to represent a set of primary and support activities undertaken by an organisation to create value for its customers and, thus, to enhance the organisation's competitiveness (Porter, 1985).

To this end, Kaplinsky and Morris (2000) explained a value chain as ‘the full range of activities which are required to bring a product or service from conception, through the different phases of production (involving a combination of physical transformation and the input of various producer services), delivery to final consumers, and final disposal after use’. The simplest characterisation of a value chain, therefore, includes four links: design, production, marketing and consumption.

However, agricultural value chains are more complex than this simple characterisation. For example, the FAO guidelines on ‘developing sustainable food value chains’ include two additional stages between production and marketing: ‘aggregation’ and ‘processing’ (Neven, 2014). Aggregation refers to the process of aggregating and storing the produce of a multiplicity of small farmers by intermediaries, while processing refers to the physical processing of agricultural goods (e.g., pulping and drying coffee beans).

**Figure 1:**
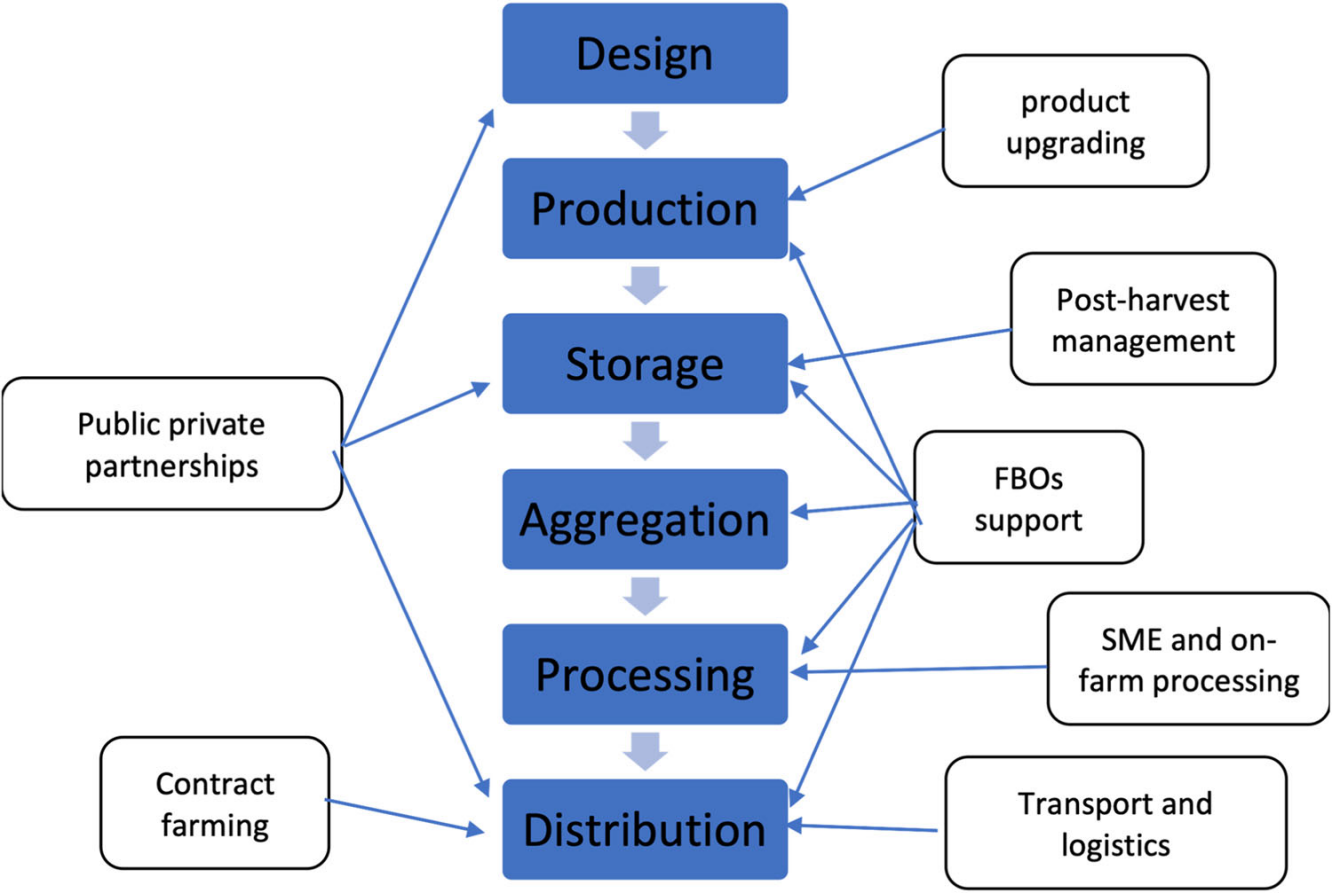
Agricultural value chain and associated interventions. FBOs, farmer‐based organisations; SME, small and medium‐sized enterprise.

Figure 1 illustrates the typical nodes of an agricultural value chain, as well as a tentative classification of ‘value chain interventions’ indicating the node of the chain in which they primarily operate. The tangle of arrows leading from interventions to the nodes in the chain shows how interventions can address constraints at different points in the chain simultaneously. Value chains are embedded within institutional, economic, social and natural environments that condition their operation. For example, the movement of products along the chain will be conditioned by legal frameworks on quality standards, availability of transport infrastructure, or vulnerability to droughts.

Value chains may develop different levels of complexity. A useful distinction is made between traditional, transitional and complex value chains (De Brauw & Bulte, 2021; Barrett et al., 2022). Traditional value chains, often related to subsistence crops, are localised and include few nodes, sometimes directly connecting producers to consumers. Transitional value chains, often involving ‘cash crops’, include more nodes in the chain, such as storing and processing, and a complicated distribution system.

Complex value chains, normally based on internationally traded crops, are articulated in several nodes, and include the participation of several actors, some of which are large firms. In complex value chains, goods travel long distances, and relationships between actors are regulated by contracts and other institutional arrangements that certify the quality of the products or insure their value.

**Figure 2:**
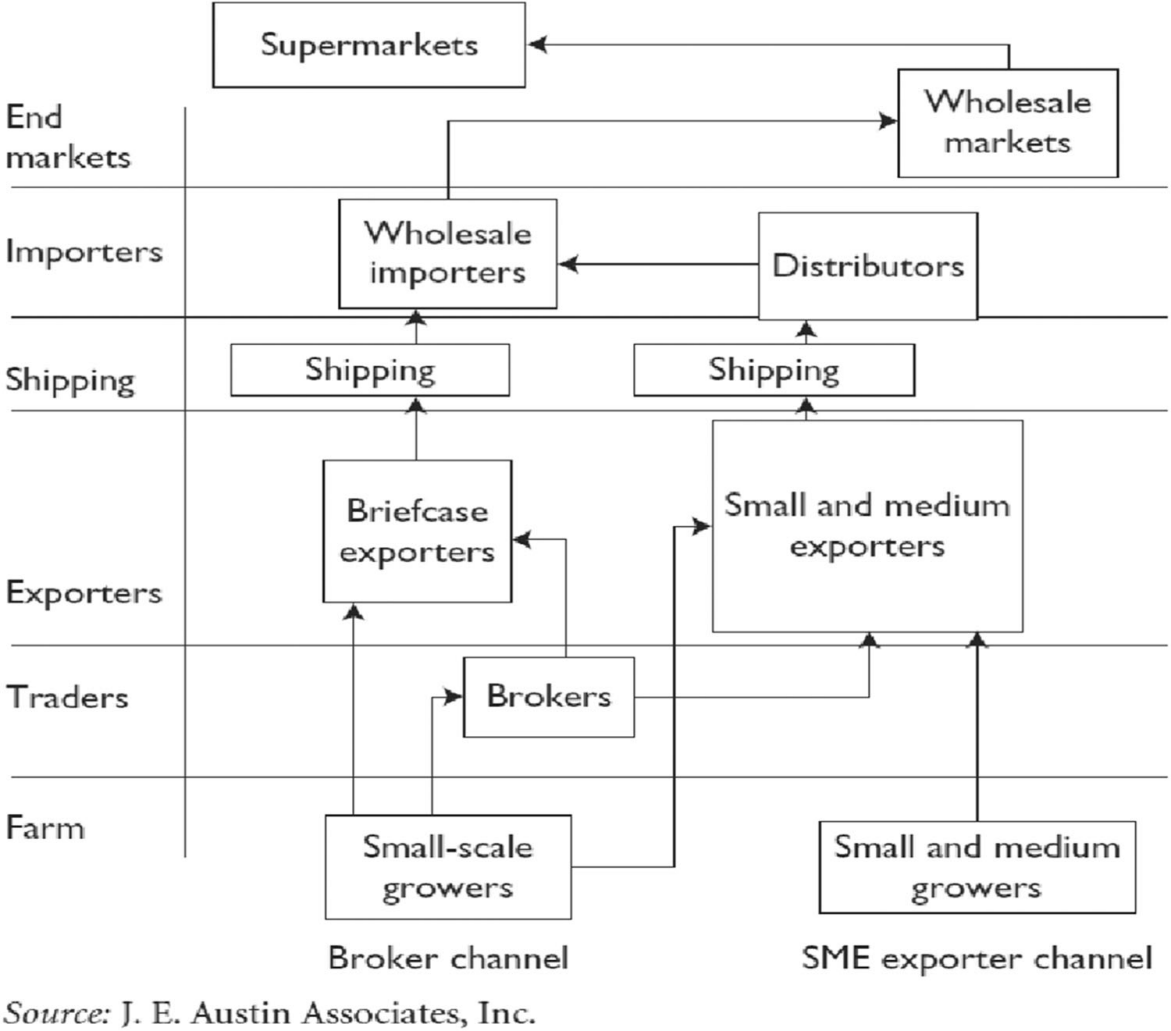
Example of a value chain: Green beans in Kenya. SME, small and medium‐sized enterprise. *Source:* Webber and Labaste (2010).

In practice, value chains are much more complex. For example, Figure 2 provides a representation of a value chain of green beans in Kenya. It displays a higher degree of complexity than Figure 1; however, value chain diagrams in the literature tend to be more complex than this. Typically, they are composed of several vertical supply chains, which illustrate the journey of a product from conception to consumption. The same product is often supplied through different channels in the same country, resulting in several vertical chains in the same value chain.

The functions performed at each link can be read horizontally: for example, in Figure 2, importers can be ‘wholesale importers’ or ‘distributors’, and end markets may consist of ‘supermarkets’ or ‘wholesale markets’. The entire value chain is therefore a matrix of functions and channels.

Value chains are built around specific agricultural products. There is no common definition of agricultural products. The FAO FAOSTAT database includes the following items: primary crops (e.g., rice, wheat), processed crops (e.g., cotton, oil, wine), live animals (e.g., goats, pigs), primary livestock products (e.g., milk, meat, eggs) and processed livestock (e.g., butter, cheese, silk). Forestry products include fuel wood, industrial wood and processed woods (e.g., paper, pulp). Fishery products include all fish varieties, aquatic plants, aquatic animal products (e.g., pearls, corals), aquatic animals (e.g., frogs, crocodiles, crustaceans, molluscs) and mammals (e.g., whales, seals).

Webber and Labaste (2010) and Haggblade and colleagues (2012) provided several examples of value chains including sorghum beer in Botswana, dairy products in Kenya, domestic catfish in Kenya, floriculture in Uganda, green beans in Kenya, cashews in Mozambique, pineapples in Ghana, coffee in Rwanda, cotton in Zambia and avocados in Kenya.

**Figure 3:**
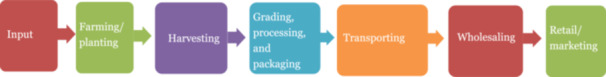
Value chain processes.

The core stages along the value chain are *production, aggregation, processing* and *distribution* (wholesale and retail). Embedded in each of these stages are several value chain interventions. In Figure 3 below, we present the value chain stages that all products went through (Morioka & Nicholas, 2014).

In value chain analysis, the following strategies are identified to improve farmers' incomes: vertical coordination, horizontal coordination and upgrading. These can be interpreted as fundamental mechanisms that are expected to operate in isolation or in conjunction with other value chain interventions. All these mechanisms add value to products along the chain and/or allow the actors to capture a higher fraction of the added value.

One finds the mechanisms articulated by different terminologies. Multiple terms used to identify the same mechanism (e.g., ‘functional upgrading’, ‘vertical integration’, or ‘value chain deepening’) could affect the clarity with which such discussions occur. Despite the multiple differences in terminologies, we identify the following mechanisms as vital to operation within value chain interventions:

*Vertical coordination*. This refers to establishing links between market agents along the value chain running from production to final consumption. New links can be established between farmers and buyers that can offer more remunerative prices (contract production). For example, farmers may sign profitable supply contracts with supermarket chains. Furthermore, farmers can undertake operations related to connected links (vertical integration), such as producers taking charge of the first stages of processing the product before selling it, thus adding considerable value to the end product.

*Horizontal coordination*. Farmers at one particular node of the value chain may aggregate and coordinate activities through farmer‐based organisations or co‐operatives. There are many potential benefits arising from coordination, such as an increase in bargaining power when negotiating prices, the exploitation of scale economies (e.g., through collective purchase of machinery), risk pooling, and other reductions in transaction costs.

*Upgrading*. Activities along the chain can be improved in several ways, and authors often distinguish ‘product’ from ‘process’ and ‘functional’ upgrading.^1^ Specifically, farmers may obtain higher prices for their goods by improving quality standards (e.g., through certification, fair‐trade or organic farming) or producing new goods altogether. Several efficiency gains can be achieved by organising the production process in different ways, or by differently mixing production and processing activities.

The distinction between these types of strategies is not always implicit and overlaps are possible. For example, horizontal integration (the organisation of farmer‐based organisations) may lead to vertical integration (producers' farmer‐based organisations taking charge of processing activities along the chain). Product upgrading (e.g., organic farming) may lead to vertical contract production as farmers gain access to specialised buyers. In general, a strategy targeting a particular link in the chain will have ripple effects on other links, and value chain interventions will often encompass a combination of the strategies above.

More recently, strategies for gender‐sensitive and gender‐responsive value chain interventions have been developed by several agencies and international NGOs. These strategies, as discussed below, aim to provide consolidated support for women to access the benefits offered by value chain interventions.

First, the gendered nature of tasks in rural economies often pushes women toward less remunerative nodes in the value chain, such as agricultural labour, petty trading and subsistence farming. There is scope to strengthen the value chain positions currently occupied by women and to enable their participation in more remunerative positions. However, to do this, it is first essential to identify and address the barriers faced by women who are willing to participate in the agricultural value chain.

Second, women are less likely to adopt new technology in their activities, primarily due to the very reasons that prevent their participation in profitable nodes in the value chain.

To this end, the FAO guidelines for sustainable value chains in particular list the following factors: poor access to information and training, limited participation and decision‐making power within household and communities, limited access to financial services and other productive assets and resources, and excessive work burdens. These factors are in turn embedded in reinforcing cultural norms and institutions. Hence, interventions aiming to make the services accessible for women is required.

We can identify three principal ways in which agencies can promote gender‐responsive value chain interventions. By gender‐responsive, we mean interventions that in their design and implementation employ gender considerations.^2^ This definition will include gender‐transformative interventions directly addressing structural barriers and power relations (Cole et al., 2020), as well as more gender‐accommodative interventions that recognise gender constraints and try to release some of them but without addressing structural barriers (Interagency Gender Working Group, 2017).

*Promote women‐led nodes in value chains*. Rural economies usually operate via gender‐specific roles. Analysis of any value chain suggests that some links are entirely occupied by male farmers, while others are dominated by women. For example, women may oversee the processing of a staple crop, or retailing. An intervention may therefore decide to strengthen the node in the chain which is dominated by women to achieve the maximum benefit for them.

*Promote women's participation in male‐dominated value chains*. An intervention may decide to promote a particularly remunerative node of the value chain (e.g., linking farmer‐based organisations to buyers connected to export markets). However, this link may be dominated by male farmers. The intervention can therefore promote women's participation in farmer‐based organisations and support their presence in leadership roles in a way that shares the benefits of the intervention across gender groups. The intervention may attempt to specifically promote the inclusion of women in highly profitable value chains or more profitable nodes of the value chain that are traditionally controlled by men.

*Address discriminatory constraints to accessing value chains*. Selecting value chain activities that are dominated by women and promoting their participation in project activities may be insufficient. Fundamental constraints must be identified that prevent women from participating in or enjoying the benefits of project activities. The value chain intervention may include specific complementary interventions that are oriented toward increasing women's participation in markets and in decision‐making capacities. For example, a project might include loans specifically targeted at women, or make provisions for childcare to enable women's effective participation in the activities.

The gender‐responsive value chain interventions that we will consider in our review are:

- Value chain interventions explicitly targeting women;
- Interventions that promote and *support women's participation in value chain domains that are traditionally male dominated*; and
- Interventions that *address constraints that prevent women from participating* or accessing benefits.

### 2.2 The intervention

The review will include interventions that engage women in agricultural value chain processes in different capacities – whether by creating an enabling environment to engage women farmers, or by focusing on land access and ownership, changes in policies, and social norms (Doss & SOFA Team, 2011).

Reviews of value chain strategies by UN agencies (Stamm & Von Drachenfels, 2011) and international NGOs (Webber & Labaste, 2010) have led to the identification of the ‘value chain’ interventions in Table 1 (classified by the main strategy above). The goal of value chain interventions is to improve farmers' incomes, particularly those of small farmers. This could be achieved through factors such as better integration into markets, access to more remunerative prices, development of new and more profitable products, and adoption of functions that add value to production (e.g., processing, storage). This review will focus on analysis of value chain interventions that are also gender responsive.

**Table 1:** List of interventions.

Value chain strategy	Interventions
Vertical coordination	Contract farming Public‐private partnerships
Horizontal coordination	Promotion of farmer‐based organisations and co‐operatives
Upgrading/other	Post‐harvest management Small and medium‐sized enterprise processing development Product quality upgrading (e.g., certification, organic, fair trade) Inclusive market systems development Infrastructure development (e.g., transport) Storage and on‐farm processing

### 2.3 How the intervention might work

In this review, we have adopted the causal chain analysis, in which we will analyse the theory of change (ToC) for an intervention. We will also synthesise the findings of process evaluations and qualitative studies to identify both the barriers and facilitators to project implementation.

We have built a ToC, also referred to as middle‐level theory. It is appropriate for systematic reviews that examine different settings and populations to identify and test the assumptions under which specific interventions generate the outcomes (White, 2018).

The analysis approach is based on specifying the logic model for intervention. It will also address the following questions:

- How does the intervention work (mechanism or methodology of intervention)?
- Why does the intervention work (causal pathway)?
- What are the weakest and/or missing links in the causal chain/pathway?

The ToC aims to enable women's economic empowerment through value‐chain interventions. This could be achieved by providing access to resources, an enabling environment, agricultural inputs, and capacity building. Here, an attempt has been made to explain the causal pathway of how women's engagement in all stages of the value chain (from production to distribution) may result in their economic empowerment.

Gender empowerment is defined as ‘a process by which those who have been denied the ability to make strategic life choices acquire the ability to do so’ (Senders et al., 2012). Although the methods and domains used to measure women's empowerment may vary across studies and are context‐specific, the component of agency is of crucial significance. Therefore, in any value chain process, the intervention should consider the various dimensions of the concept. This would not only enable impact assessments of the intervention but would also acknowledge the dynamism of the context in which the intervention is introduced. To take this discussion further, let us briefly outline the important dimensions of the concept.

First, women's empowerment is understood as a process, and not an end in and of itself. It refers to women's ability to choose three interrelated elements: resources, agency and achievement. Resources such as money, education, social capital, land and credit strengthen the empowerment process by enabling women's agency. Agency refers to the ability to formulate strategic choices and control over resources and decisions. It has been believed that the precondition to achieving gender equality lies in women's agency and decision‐making power (Okesina, 2020; Senders et al., 2012).

The second aspect of women's empowerment is that of power relations. There are four types of power relations: ‘power over’, ‘power within’, ‘power to’, and ‘power with’. ‘Power over’ (Okesina, 2020; Senders et al., 2012) refers to a zero‐sum situation (i.e., one person's gain is another person's loss), which is best explained in patriarchal societies in which men control resources and decision‐making. While ‘power within’ refers to a state of confidence, dignity and self‐esteem, ‘power to’ refers to the capacity to make desired changes (i.e., the ability to exercise choice and change one's condition). ‘Power with’ refers to networks, groups and collective strength‐based collaboration and solidarity, and could be understood as a win‐win situation for both genders (Okesina, 2020; Senders et al., 2012).

Women's empowerment in terms of a power approach suggests that a combination of ‘power within’, ‘power to’, and ‘power with’ is necessary to arrive at gender equity. Such dynamics suggest that one's agency to make effective choices subtly hints at a change in power relations, as well as in that individual's autonomy and decision‐making capacity. ActionAid employs a similar concept to gauge women's empowerment. It is referred to as ‘Power Cube 5’, and analyses power constructs via the indices of ‘power within’, ‘power with’, and ‘power to’ (Morioka & Nicholas, 2014; Powercube, 2013; Senders et al., 2012).

The third aspect of women's empowerment is that of outcome. Empowerment means that there are definite outcomes in terms of change in economic, social and political constructs – not only a change in processes or power relations. Achievement aims to increase labour participation and educational attainment (Senders et al., 2012).

The fourth aspect of women's empowerment is a multi‐dimensional concept. It comes into effect through various dimensions, including personal, relational, micro, meso, and macro levels (Okesina, 2020; Senders et al., 2012).

Oxfam has explained that effective ‘women's economic empowerment can be achieved when women enjoy their right to control and benefit from resources, assets, income and their own time, and when they can manage risk and improve their economic status and well‐being’ (Kidder et al., 2017). This is exemplified when women also have autonomy and self‐belief, as well as agency and power to influence decision‐making and make changes in their lives. To understand gender in value chains, we must bring together a value‐chain development approach and gender rights‐based approach. Below we explain the chain empowerment matrix through a gender lens (Senders et al., 2012).

#### 2.3.1 Chain empowerment matrix

**Figure 4:**
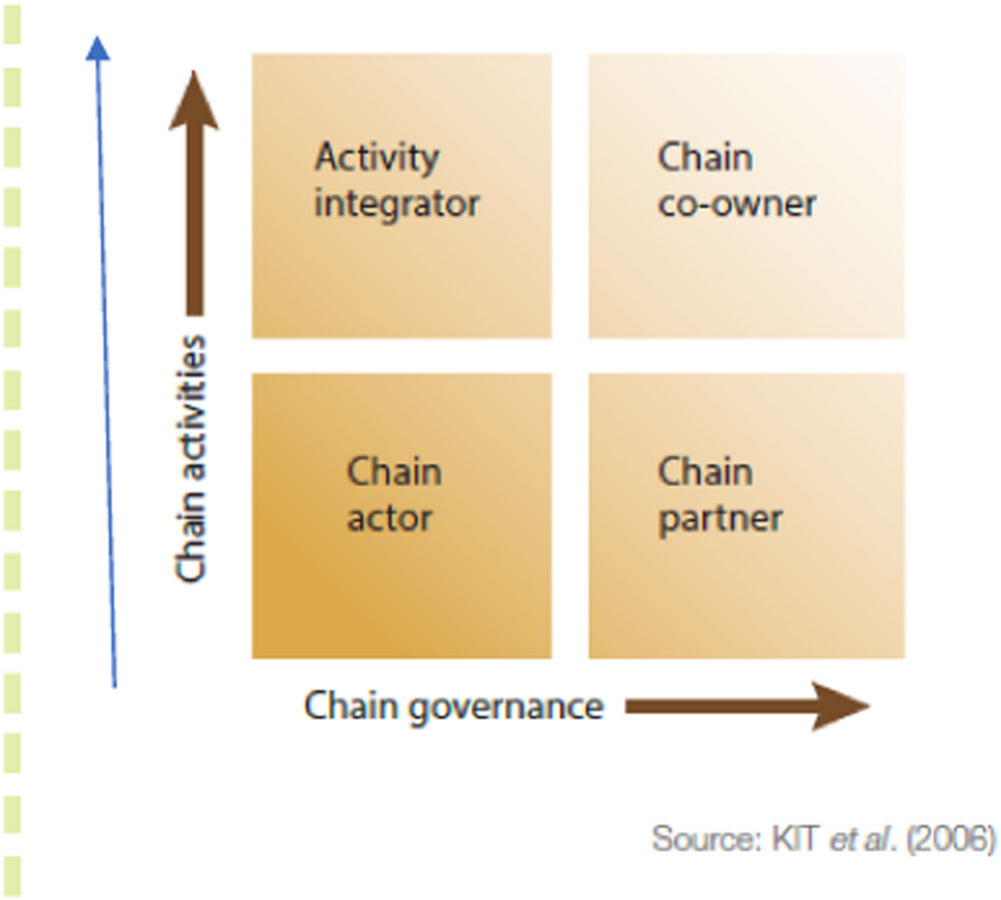
Chain empowerment matrix.

The chain empowerment matrix (Figure 4) is a useful framework^3^ to explain chain development. There are two broad dimensions through which to understand types of participation within a chain. While chain activities refer to those undertaken by farmers (i.e., who does what), chain governance refers to farmers' involvement in chain management (i.e., who determines how things are done) (Senders et al., 2012).

To explain further, farmers can undertake different activities such as drying or fermentation of their crops (post‐harvest activities), or grading, processing, transporting and trading. Vertical integration/coordination is understood as involvement in various activities in the chain. Farmers concerned with management of the chain are usually involved in decision‐making processes and control over management issues. Farmers are in control of decisions such as sale quantities and prices. Involvement in multiple chain management issues is known as horizontal integration/coordination (Senders et al., 2012).

Participants' ability to conduct value chain activities (e.g., processing, post‐harvesting) more efficiently and profitably could result in chain empowerment. It reduces transaction costs by integrating activities that were previously handled or controlled by others. Participants gain control over the value chain processes through improved negotiation capacities, and build stronger relationships with other stakeholders in the value chain (Morioka & Nicholas, 2014).

Four dimensions of gender empowerment in value chains are mentioned below (Table 2). For this review, we focus on economic empowerment.

**Table 2:** Dimensions of gender empowerment.

Vertical integration into chain	What activities do women and men in the chain undertake?What benefits do women and men gain?
Horizontal integration into chain	Who determines the conditions under which these activities are performed?How are benefits gained and distributed?
Gender dynamics in household and community	How do changes in the first two dimensions affect the gender division of labour, assets and decision–making within the household?How do changes in the first two dimensions affect the gender dynamics within the community?
Individual context: rules, norms and values	Which economic, political and social factors enable or constrain women's empowerment in the other three dimensions?How do changes in the first two dimensions influence the institutional context?

It is important to note that globally, approximately 75% of women farmers play active roles as producers, traders, processors, labourers and entrepreneurs; however, they are disadvantaged as their roles are largely unrecognised (Gurung et al., 2015). This is due to the many constraints they face, such as limited access to land, irrigation, productive inputs, financial credit, property rights, new technologies and markets (Agarwal, 2018; Doss & SOFA Team, 2011; Gurung et al., 2015; Mayoux & Mackie, 2008; Mutua et al., 2014; Rubin et al., 2010; Ugwu, 2019).

The evidence suggests that gender differences and inequalities exist at all levels of the value chain, and are the main barriers to women's participation in agricultural activities (Gurung et al., 2015; Ugwu, 2019). Other barriers include: (1) customs, beliefs and attitudes that confine women to the domestic sphere; (2) women's workload and time poverty; and (3) laws that affect access to resources, employment and education. It has been further elaborated that women's participation in markets is restricted due to a lack of accessible transportation, as well as social norms that prohibit them from travelling (Mayoux & Mackie, 2008; Quisumbing et al., 2013; Ugwu, 2019). The intended outcomes of some value chain interventions entail addressing of these constraints – particularly by increasing women's free time, mobility, income and decision‐making.

**Figure 5:**
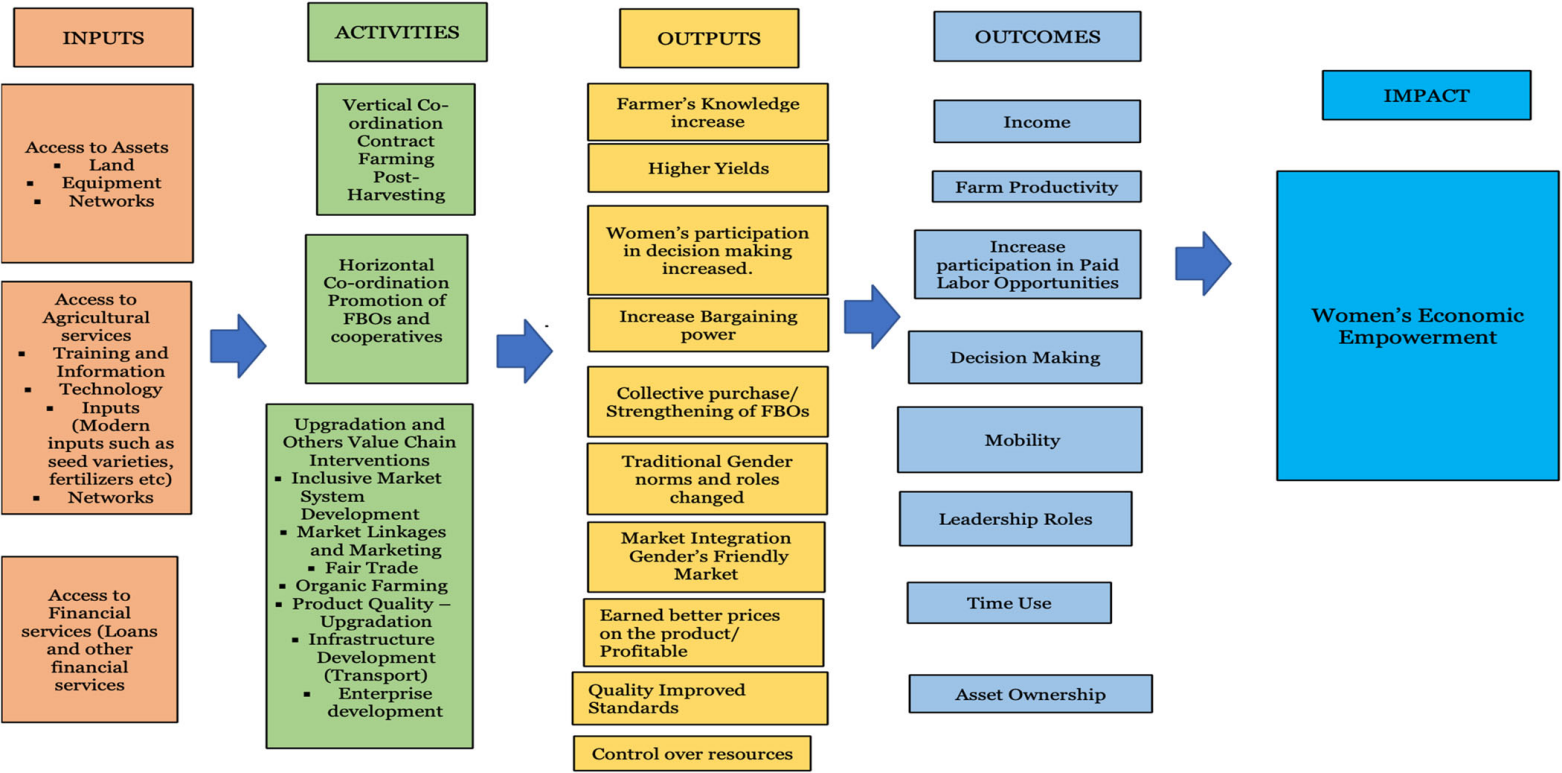
Theory of change. FBOs, farmer‐based organisations.

For the ToC, we combine the existing approaches and focus on interrelated dimensions, access to productive resources, power, and agency. The same approach was adopted by FAO.^4^ The visual representation in Figure 5 shows the causal process through inputs – meaning access to productive resources that facilitate activity implementation (value chain interventions in horizontal and vertical coordination and upgradation) – which in turn help to achieve the immediate outputs as a chain actor and activity integrator (increase in knowledge and skills), and outcomes such as chain co‐owner and chain partner (decision‐making). This helps to achieve the outcome/impact of women's economic empowerment.

The left side of the figure lists the value chain interventions:

- Vertical coordination (contract farming [production]);
- Horizontal coordination (promotion of farmer‐based organisations); and
- Upgrading (fair trade, upgrading product quality).
  The objectives of the value chain intervention mentioned are as follows:
- To enhance women's roles in agricultural value chains (e.g., increasing their involvement in specific nodes or stages of the value chain such as processing or marketing); and
- To expand opportunities for women (Quisumbing et al., 2013).

This ToC explains the inputs and activities required to attain the desired intermediate outcomes and, ultimately, women's economic empowerment. The casual pathways in Figure 5 can be followed from left to right. In the proposed ToC, we illustrated how access to inputs (e.g., fertilisers, credit, loans, information, training) can lead to the intended outcomes.

The value chain interventions may provide or promote women's contributions and aim for their economic empowerment (e.g., processing or storage technologies that add value to the product, which we would consider under ‘post‐harvest management’ and ‘processing development’ interventions). The approach of ‘power with’ – such as the establishment and strengthening of farmers' groups, collectives, and more female farmers' groups – helps to improve income, decision‐making, bargaining, women's participation, and economic empowerment.

Embedded in each of the stages are several value chain interventions; our ToC explains these, as well as how they may lead to women's economic empowerment. We assume that women's participation in the value chain as actors, partners and co‐owners offers opportunities for economic benefit and empowerment.^5^

There are various stages of value chain processes and involving them all in this ToC risked overcomplication. In the protocol, we listed classifications of interventions and intermediate outcomes. The current ToC is tentative, and one aim of our work is to build one that is theory‐informed (or middle‐range).

To achieve the outcomes – considering empowerment as an outcome – we have some assumptions. The first is that women have access to resources such as irrigation, productive inputs, financial services and new technologies. It has been also assumed that women actively participate and have access to knowledge and training. There is evidence that women engaged in farming are often exploited by other actors in the value chain (buyers and suppliers) due to their high levels of illiteracy, weak bargaining power, and lack of knowledge and access to market information (Gurung et al., 2015). We assumed that value chain interventions helped to address the above‐mentioned constraints to achieve the intended outcomes.

The review will include the value chain interventions which engage women in agriculture value chain processes in different capacities. The interventions aim to increase women's rights and economic power. The interventions targeted the community to create an enabling environment to engage women farmers. It can also focus on land access and ownership, changes in policies, and social norms (Doss & SOFA Team, 2011).

A review of value chain strategies by UN agencies (Stamm & Von Drachenfels, 2011) and international NGOs (Webber & Labaste, 2010) led to the identification of the ‘value chain’ interventions in Table 1, which are classified by the main strategy above. The goal of value chain interventions is improvement in farmers' incomes, particularly small farmers. This should be achieved through better integration in markets, access to more remunerative prices, the development of new and more profitable products, and the adoption of functions that add value to production, such as processing or storage.

### 2.4 Why it is important to conduct the review

A literature review suggests that there are no systematic reviews on this topic. Several reviews address similar or overlapping questions, including the following:

Gibbs, A., Willan, S., Misselhorn, A., & Mangoma, J. (2012). Combined structural interventions for gender equality and livelihood security: A critical review of the evidence from southern and eastern Africa and the implications for young people. *Journal of the International AIDS Society*, *15*, 1–10.

Riisgaard, L., Fibla, A. M., & Ponte, S. (2010). *Evaluation study: Gender and value chain development*. Copenhagen: The Evaluation Department of the Danish Foreign Ministry.

Stewart, R., Van Rooyen, C., Korth, M., Chereni, A., Da Silva, N. R., & De Wet, T. (2012). *Do micro‐credit, micro‐savings and micro‐leasing serve as effective financial inclusion interventions enabling poor people, and especially women, to engage in meaningful economic opportunities in low‐ and middle‐income countries? A systematic review of the evidence*. EPPI‐Centre.

As is evident, there remains a significant gap in evidence on the impact of women's engagement in agricultural value chains and markets on their economic empowerment, and the barriers to achieving this.

## 3 OBJECTIVES

The review will address the following research areas:

1. The primary objective of this review is to understand and evaluate what approaches, strategies or interventions focused on women's engagement in agricultural value chains and markets have led to women's economic empowerment in low‐ and middle‐income countries.
2. The secondary objective of this review is to examine the contexts in which these approaches are effective (or ineffective). What are the contextual barriers and facilitators determining women's participation in, and benefit from, engagement in the value chain in low‐ and middle‐income countries (programme effectiveness)?
3. Finally, this review aims to refine the ToC that describes how value chain interventions lead to women's economic empowerment, using evidence drawn from both rigorous quantitative impact evaluation studies and qualitative studies.

## 4 METHODS

### 4.1 Study selection criteria

Studies were included in the review if they met the selection criteria below.

#### 4.1.1 Types of participants

Included reviews examined women of all ages from low‐ and middle‐income countries^6^ engaged in agriculture and food systems and targeted by value chain development and market engagement interventions. Population subgroups included smallholder farmers, value chain entrepreneurs, or employees in the agribusiness sector.

#### 4.1.2 Types of interventions

The interventions of interest under this review are those aiming to empower women through value chain development and market engagement implemented in the agricultural sector. These interventions aim to increase women's market participation in the value chain by removing barriers to participation, or by changing social norms in organisations, communities, and the family to facilitate participation.

The interventions may include the following:

- Inclusive market systems development
- Horizontal coordination (promotion of farmer‐based organisations and co‐ops)
- Vertical coordination (contract farming, public‐private partnerships)
- Post‐harvest management
- Gender‐friendly markets (lighting, washroom facilities, provision for childcare)
- Enabling policies and institutional environments
- Financial services (grants and subsidies, micro‐credit, savings and insurance)
- Processing and storage facilities
- Process, product, and chain upgrading
- Enterprise development and impact investing
- Infrastructure development (transport)
- Promoting the production of a new profitable product
- Improving product market quality (fair trade, organic farming, quality standards)
- Supporting horizontal integration of producers' groups to access better prices
- Improving processing techniques (on‐farm processing)

#### 4.1.3 Types of outcome measures

The outcomes of interest for this review include measures that result in women's economic empowerment and the various dimensions embedded in this (Supporting Information: Appendix E). The listed indicators reflect the main areas of empowerment within agricultural value chains and markets.

Not all studies will report impacts in terms of empowerment outcomes. We also consider other outcomes along the project causal chain (Table 3); these do not characterise empowerment but are often preconditions to it. In particular, we consider:

- Economic outcomes, such as incomes, prices and market sales; and
- Participation outcomes, such as women's participation in labour markets, membership of farmers' organisations, and leadership positions in groups.

**Table 3:** Outcome categories and subcategories.

Outcomes	Indicators
Economic empowerment	Multidimensional empowerment indices such as the Women's Empowerment in Agriculture Index and other ad hoc indices built by researchers Agency indicators: Decision‐making on value chain activities, decision‐making over the use of income, increased bargaining power, leadership positions in groups, mobility
Economic benefits	Farm productivity Income Time use Assets ownership
Participation	Access to information on production/markets Enhanced social and institutional networks Increased participation in paid labour opportunities Access to new markets Knowledge and skills Gender roles and norms

#### 4.1.4 Types of studies

To answer Objective 1, we used an experimental or quasi‐experimental design. Eligible designs included those in which the authors use a control or comparison group, and in which one of the following is true:

- Participants are randomly assigned (using a process of random allocation, such as a random number generation);
- A quasi‐random method of assignment has been used and pre‐treatment equivalence information is available regarding the nature of the group differences (and groups generated are essentially equivalent);
- Participants are non‐randomly assigned by relevant demographic characteristics (using observables, or propensity scores) and/or according to a cut‐off on an ordinal or continuous variable (regression discontinuity design); or
- Participants are non‐randomly assigned, but statistical methods have been used to control for differences between groups (e.g., using multiple regression analysis including difference‐in‐difference, cross‐sectional [single differences], or instrumental variables regression).

No restriction will be placed on the duration of follow‐up.

The evidence incorporated under Objective 2 is broader, to answer that we included impact evaluations, as well as studies collecting and analysing qualitative evidence. Fulfilling Objective 3 involved the examination of plausible pathways, barriers, and facilitators of included studies across contexts, from the evidence included in the review, and a conceptual framework will be articulated.

### 4.2 Search strategy

#### 4.2.1 Electronic searches

We used the strategies to identify completed and on‐going potential studies mentioned in Supporting Information: Appendix A. The search included the following databases: Scopus, Web of Science Core Collection (Social Sciences Citation Index [SSCI], Science Citation Index Expanded [SCI‐EXPANDED, Conference Proceedings Citation Index – Science [CPCI‐S], Conference Proceedings Citation Index – Social Science & Humanities [CPCI‐SSH], and Emerging Sources Citation Index [ESCI]), International Bibliography of the Social Sciences, EconLit, Business Source Premier, APA PsycInfo, Cochrane Central Register of Controlled Trials, Cochrane, Database of Systematic Reviews, CAB Abstracts and Sociological Abstracts.

Supporting Information: Appendix A also presents an example of the keyword strings used for publication databases and search engines, with terms for interventions, regions, and methodologies. We also conducted a machine‐learning assisted search in EPPI‐Reviewer beta version (Open Alex). Open Alex data set, like Google Scholar, is a comprehensive repository of research articles containing 250 million bibliographic records. It searches not only academic databases but also grey literature sources.

We also conducted a grey literature search from organisation websites such as: CGIAR; the International Fund for Agricultural Development; AgriProFocus; the Bill & Melinda Gates Foundation; Donor Committee for Enterprise Development; the UN Food and Agriculture Organisation; the International Labour Organisation; the Netherlands Development Organisation; USAID; the Swiss Agency for Development and Cooperation; the International Food Policy Research Institute; World Agroforestry; the International Livestock Research Institute; the Foreign, Commonwealth & Development Office; the British Library for Development Studies; AGRIS; IMMANA grant database; the 3ie impact evaluation database; Innovations for Poverty Action; J‐PAL; the World Bank IEG evaluations; the USAID Development Data Library; Experience Clearinghouse; the proceedings of the Agriculture, Nutrition and Health Academy conference; the proceedings of the Centre for the Study of African Economies Conference; the proceedings of the North East Universities Development Consortium Conference; and the World Bank Economic Review.

### 4.3 Data collection and analysis

#### 4.3.1 Screening and study selection

The screening for inclusion or exclusion was undertaken in two stages by two independent researchers in EPPI‐Reviewer. The first stage title and abstract screening were performed using EPPI‐Reviewer's machine learning software. The second stage involved full text screening. The screening was performed with the inclusion criteria for this review and a third‐party arbitrator in case of disagreement (SKM). The tool was developed based on PICOS for screening the studies to decide on eligible studies (Supporting Information: Appendix B).

#### 4.3.2 Data extraction and management

For impact and process evaluations/qualitative studies, we used a standardised data extraction form (Supporting Information: Appendix C) to extract descriptive data from all the studies that met our inclusion criteria. All outcome data was coded, with different measures of the same outcome in the same study being combined in a multi‐level meta‐analysis. Data extraction from each study included context/geographical information, population, study design and method, intervention types and outcomes type, and subcategory. Two researchers independently conducted the data extraction for each study. Both coders were trained on the tool before starting. Disagreements were resolved through discussion with a third reviewer, who was consulted as needed (SKM).

#### 4.3.3 Assessment of risk of bias in included reviews

Confidence in the findings of all studies included in the review was assessed using a critical appraisal tool for primary studies developed by the Campbell Collaboration Secretariat. The tool covers both quantitative and qualitative studies (see Supporting Information: Appendix D: Critical appraisal tool, along with a description of how the tool was developed). Critical appraisal assessment was completed by two reviewers.

The tool contains critical dimensions of the evaluation. Each of these is marked as high, medium, or low. The overall score uses the ‘weakest link in the chain’ principle. Hence, the confidence in study findings can only be as high as the lowest rating given to the six critical items in an effectiveness study, and nine critical items in a qualitative/process evaluation.

#### 4.3.4 Data analysis

##### Unit of analysis issues

The unit of analysis for the quantitative data was individuals and/or households. Multiple papers or reports based on the same study or data were treated as a single case (a unit of analysis is the case, not the paper). The report or paper was treated as a separate case when the study sample did not include study participants included in any other coded study.

##### Criteria for determination of independent findings

In multiple papers or reports, we selected the revised or updated version if all relevant information was available in a single source. However, if the multiple reports provided different information (e.g., different outcomes or different subgroups), the data from these reports were coded as a single case, taking different information from each study.

##### Dealing with missing data

Study authors were contacted if we required additional data that were missing or incomplete. In cases of non‐availability or no response from authors, we reported the characteristics of the study, but did not include such a study in the meta‐analysis.

##### Assessment of heterogeneity

Heterogeneity between effect sizes was assessed by reporting the *I* ^2^. Forest plots were generated to provide a visual representation of pooled effect size. The causes of heterogeneity, if any, were identified through visual inspection and moderator analysis. Separate forest plots were presented for important moderators.

##### Assessment of reporting biases

Publication‐selection bias was assessed for the primary outcomes by constructing a funnel plot for each of the two outcomes (Higgins & Green, 2011). The funnel plot was used for a trim‐and‐fill analysis and the calculation of Egger's test.

#### 4.3.5 Data synthesis

In this review, we computed the standardised mean difference (SMD) from the available information found in primary studies such as means, regression coefficients, or chi‐squared tests from analysis of variance. We computed the OR from available information on proportions and frequencies. The effects size calculation performed based on the effect size coding tool developed for this review, and, in a minority of instances the Campbell Online Effect Size Calculator.^7^

Meta‐analysis of effect sizes for each outcome were conducted using Stata. A weighted mean effect size for each outcome was reported under a random‐effects model. Overall effect sizes for primary outcomes were reported in the common metric of odds ratios to communicate with policymakers and practitioners.

##### Subgroup analysis and investigation of heterogeneity

Moderator analyses of a single categorical variable were conducted using a subgroup analysis, analogous to an analysis of variance, also under a random‐effects model. Moderator analyses of continuous moderators for multiple moderators were conducted using meta‐regression analysis and reported under a random‐effects model.

Our a priori planned moderator analyses include: (1) targeted interventions versus non‐targeted interventions; (2) types of the research design; and (3) region. *Post hoc* moderator analyses may be estimated if the qualitative synthesis suggests patterns of heterogeneity in the data that may be explored in quantitative analysis.

#### 4.3.6 Sensitivity analysis

The sensitivity analysis was performed by removing studies from the meta‐analysis one by one to see if the results of the meta‐analysis are sensitive to any single study. We will also examine the sensitivity of findings by risk of bias (low risk, some concerns, and high risk).

#### 4.3.7 Treatment of qualitative research

In this review, we adopted an approach that combined the qualitative data with the quantitative meta‐analysis, within the framework of a theory‐based systematic review (White, 2018). In the framework, the unit of analysis is the intervention, rather than an individual study. Different studies may contribute findings at various stages of the casual chain. For example, process evaluations and qualitative studies provide more evidence on implementation issues than most effectiveness studies (such as a lack of women's involvement in the programme and why), which can help to explain both the size of, and variations in, effect sizes.

The framework is shown in Table 4. Quantitative data are indicated as Qt and qualitative as Ql. Quantitative data refer to both effect sizes and factual quantitative data, such as participation rates.

**Table 4:** Stages of the causal chain with data to be examined at each stage.

Stage in the causal chain	Data
Awareness of the programme amongst relevant service providers and target group	Know of programme, aware of eligibility criteria, purpose, and how to access (Qt/Ql)
Enter the programme Stay with programme for whole duration	Attrition (Qt) Reasons do not participate or remain in programme (Ql)
Activities undertaken	Descriptive material (Ql)
Enabling environment	Change in social norms (Ql)
Strengthen existing groups and more involvement of women	Farmer groups, co‐operatives, and women's involvement/engagement (Qt and Ql)
Connection to services (access to markets, credit/savings)	Access to services (Qt and QI)
Economic impact and participation	Increase in income, agricultural productivity, ownership, skills and knowledge (Qt supported by Ql)
Women's empowerment	Women's economic empowerment (Qt and QI)

Table 4 shows the theory‐based systematic review TBSR framework, which is used for both horizontal and vertical synthesis (White, 2018). In Table 5 an abbreviated version of the row headings from Table 4 are pivoted to become column headings. The data in Table 4 are subject to vertical, horizontal, and total synthesis.

**Table 5:** Theory‐based systematic framework.

	Participation	Activities	Enabling environment	Services	Economic impact and participation	Women's empowerment
Case 1						Horizontal synthesis
Case 2						
‐‐‐						
Case *n*						
	Vertical synthesis					Overall synthesis

Vertical synthesis involves summarising the evidence across all cases, which is the way in which systematic reviews are usually performed, especially for quantitative analysis of effects. In the case of qualitative data, vertical synthesis is a thematic analysis, in which common themes are identified across studies.

Horizontal synthesis summarises across a case – which may be performed through in narrative reviews; however, the difference here is that the data for an intervention may be drawn from more than one study. The overall synthesis combines both, though it may well contain separate overall synthesis by subgroup. The overall synthesis approach, drawing on both horizontal and vertical synthesis, ‘tells the story’ of whether the intervention works, for whom, under what circumstances, and why.

## 5 RESULTS

### 5.1 Description of studies

The database search identified 11,631 studies out of which 2854 were duplicates, leaving 8786 studies for the title and abstract screening, plus an additional 10 benchmarking studies.^8^ We have also identified 423 records from Open Alex. Of these, 3831 studies were screened for full text. We have excluded 3595 studies at the full‐text screening stage.

**Figure 6:**
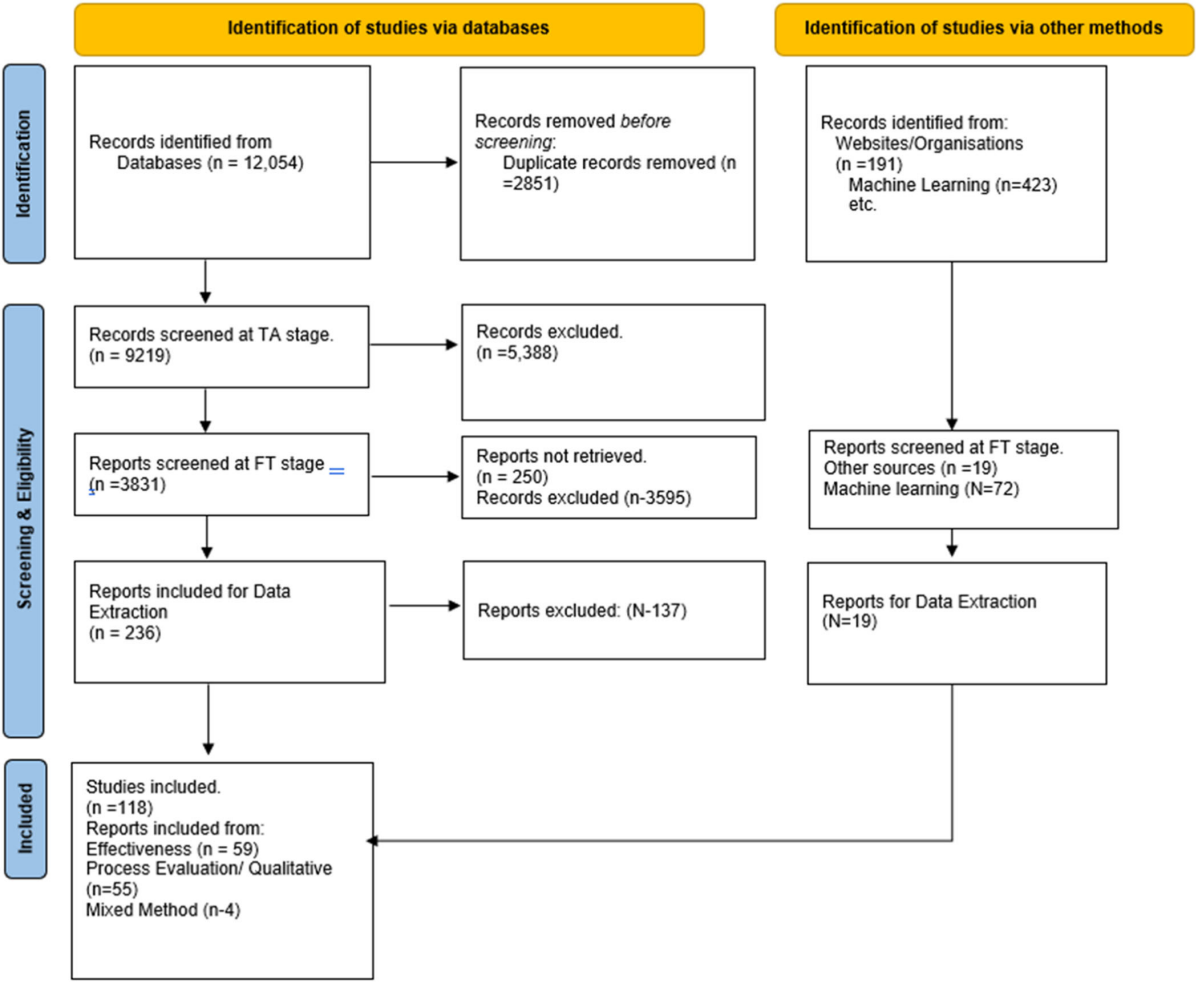
PRISMA diagram. E, number of effectiveness studies; MS, number of mixed‐methods studies; *N*, number of studies; P, number of process evaluations/qualitative.

Finally, we have included 236 studies for coding 19 studies from the grey literature search. Among them, 89 were effectiveness studies, 15 were mixed methods, and 151 were process evaluations. From these, we excluded 46 effectiveness studies, 95 qualitative and process evaluations, and 11 mixed‐methods studies due to study methodology, or the intervention not clearly defined as ‘value chain’ (See Supporting Information: Appendix G – list of excluded studies). We have also identified 17 effectiveness studies and 2 process evaluations from the grey literature search (Figure 6). The final number of included studies in the review is 59 effectiveness studies, 4 mixed‐methods studies, and 55 qualitative and process evaluations from database searches (Table 6).

#### 5.1.1 Characteristics of the included studies

The studies included:

- 4 mixed‐methods studies;
- 59 effectiveness studies; of which
  ○ 4 are randomised control trials, and
  ○ 55 are non‐experimental effectiveness studies; and

- 55 process evaluation and qualitative studies.

At the coding stage, studies were excluded that did not meet the methodological inclusion criteria (valid control group), did not evaluate a value chain intervention, or did not analyse women's economic empowerment outcomes. In some cases, we were not able to obtain the full text of the identified studies.

#### 5.1.2 Geographical area

**Figure 7:**
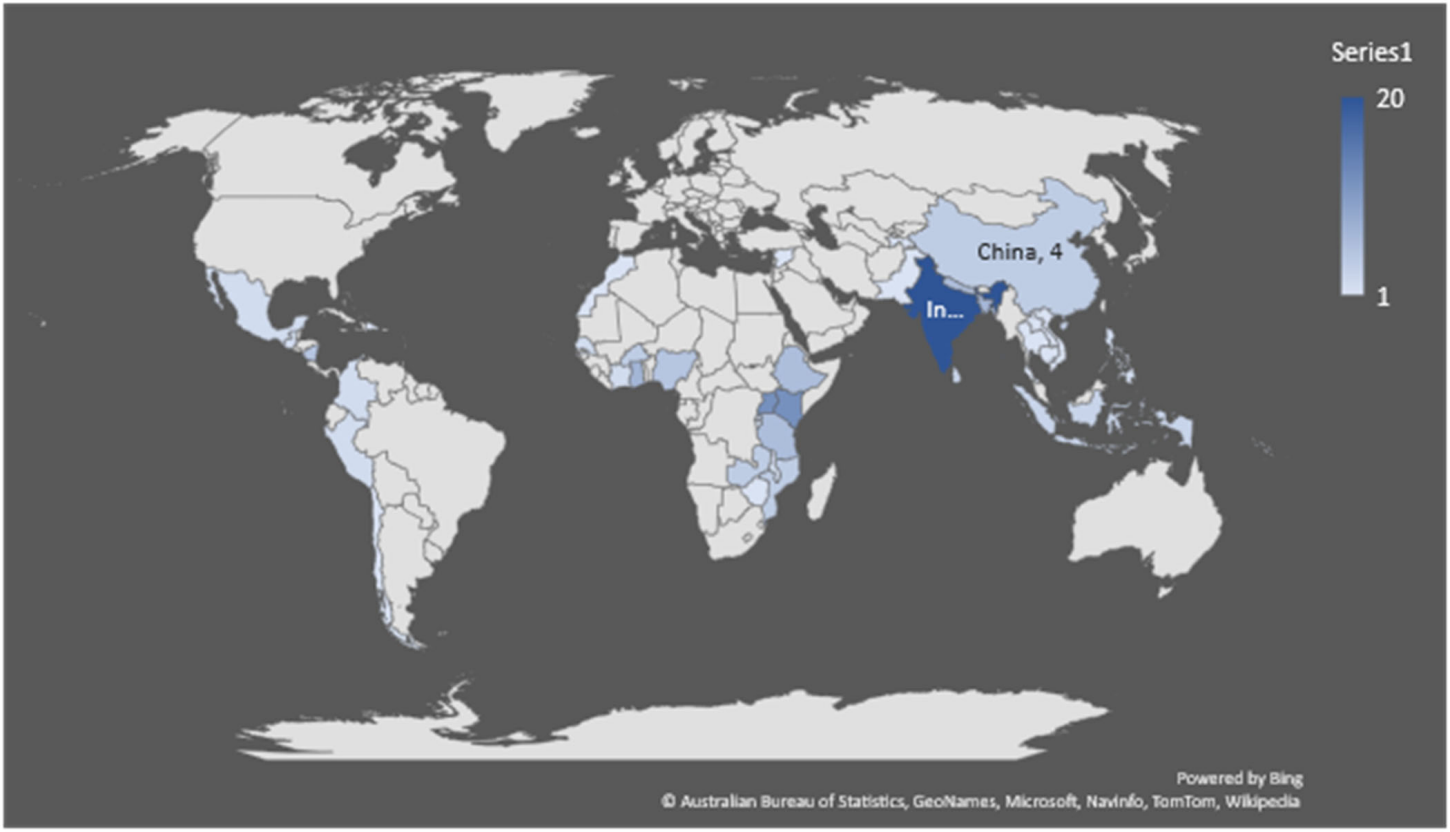
Geographical representation of the included studies.

The majority of the studies included in the analysis are from Sub‐Saharan Africa, accounting for 57% (67 studies). Approximately 30% (35 studies) are from South Asia, 8% (10 studies) from Latin America and the Caribbean, 5% (6 studies) from East Asia and the Pacific, 3% (3 studies) from South America, 2% (2 studies) from the Middle East and North Africa, and 1% (1 study) from Europe and Central Asia. The majority of the studies (63%) are from lower‐middle income countries (74 studies), followed by low‐income countries (31%, 37 studies). Only 8% (10 studies) are from upper‐middle income countries (Figure 7).

The distribution of studies across various countries shows a notable concentration in several key countries. The majority of the representation comes from India with 17% (20 studies), followed by Uganda and Kenya with 10% each (12 studies each). Following this, 6% (seven studies) represent Ghana and Bangladesh, and 5% (six studies) represent Tanzania, Ethiopia, and Nepal. Nigeria and Nicaragua each account for 4% (five studies). Similarly, China, Mozambique, Zambia, and Burkina Faso each represent approximately 3% (four studies). There are three studies from Indonesia, Rwanda, and Sri Lanka. Only one or two studies each are from the following countries: Côte d'Ivoire, Burundi, Peru, Colombia, Malawi, Senegal, Mexico, Guatemala, the Philippines, Pakistan, Syria, Chile, Zimbabwe, the Solomon Islands, Morocco, the Dominican Republic, Tajikistan, the Republic of Congo, Thailand, Laos, Vietnam, Cambodia, and East Timor.

#### 5.1.3 Study populations

##### Gender

**Figure 8:**
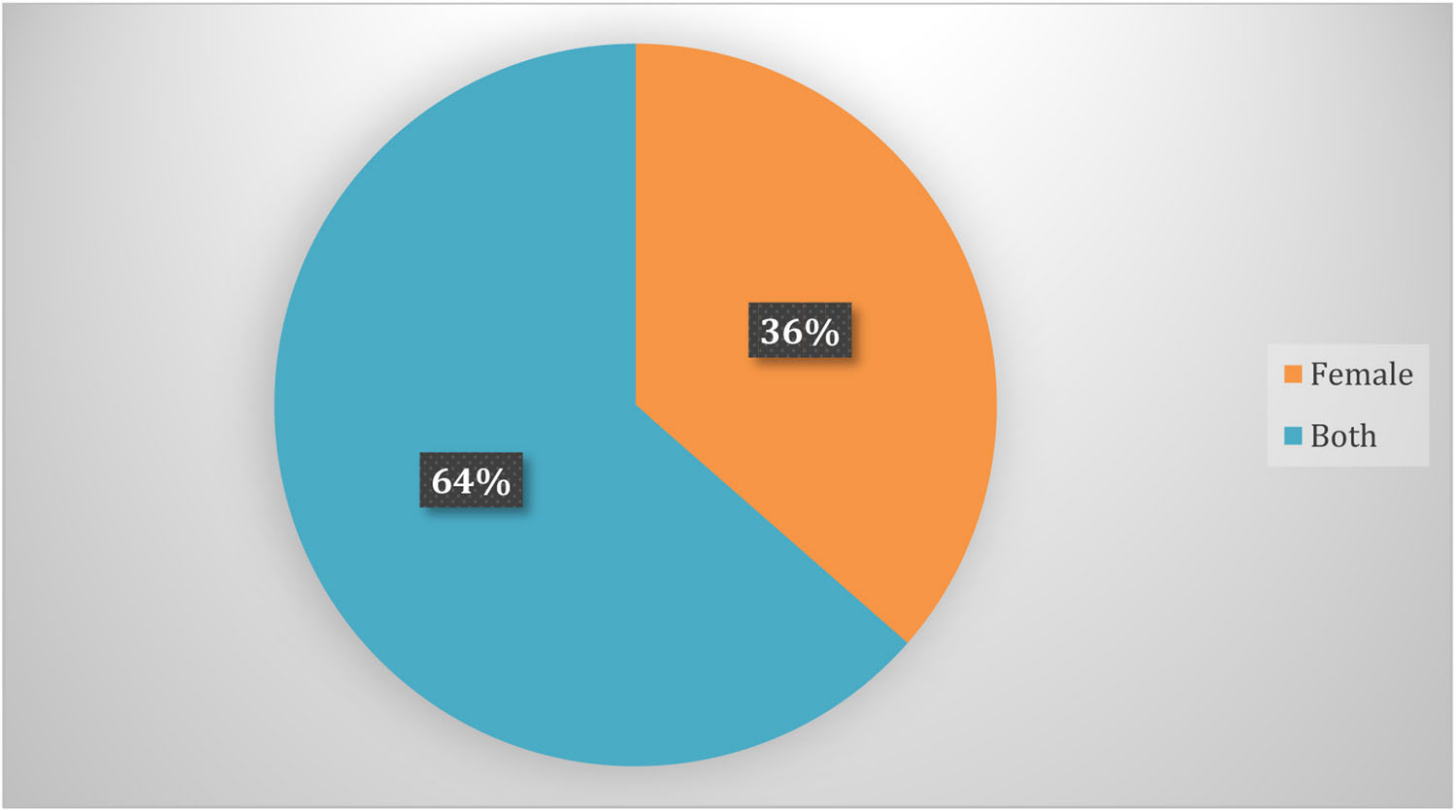
Gender representation of included studies.

A total of 64% (75 studies) included both genders, while 36% (43 studies) exclusively focused on interventions targeting women (Figure 8). There were 43 studies on interventions for women only, such as women's self‐help groups (Anand et al., 2019; Desai & Joshi, 2013; Mohapatra, 2016; Raghunathan, 2019), Grameen Ghana's microcredit scheme (Al‐Hassan et al., 2011), producer association farmers' centres (Desai, 2014), women in aquaculture (Panda et al., 2012), IMC Microcredit Programme (Pellegrina, 2021), BRAC Microfinance Programme (Namayengo, 2017), and the National Dairy Development Board programme (Papa et al., 2000).

### 5.2 Overview: Interventions and outcomes

**Figure 9:**
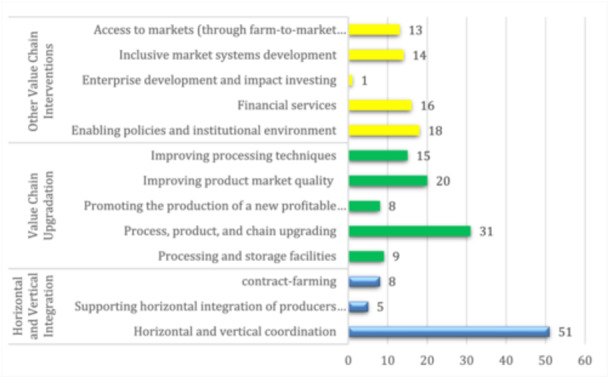
Number of studies in each intervention category and subcategory.

In this review, 54% (64 studies) evaluated horizontal and vertical integration, 70% (83 studies) looked at value chain upgradation, and 53% (62 studies) examined other value chain interventions (Figure 9). We found that the majority of studies evaluated interventions related to horizontal and vertical coordination (51 studies) such as self‐help groups, women's cooperatives, Smallholder Dairy Commercialisation Programme (SDCP) (Bonilla, 2017), certified organic cotton initiatives (Altenbuchner, 2021) SDCP Producer Associations (Desai, 2014). Additionally, 31 studies focussed on process, product, and chain upgrading interventions such as ICT‐Based Market Information Service (MIS) Projects (Asingwire, 2011) and Irrigated Rice Production Enhancement Project (Arslan, 2018). Financial services interventions were evaluated in 16 studies including Strengthening Local Development in the Highlands and High Rainforest Areas Project (PSSA) (Arslan, 2020), Agricultural Value Chains Support Project (PAFA) (Garbero et al., 2018), and micro‐credit. There were only a few studies on the production of a new profitable product, contract farming, or improved product market quality (Table 6).

**Table 6:** Characteristics of included studies.

Study name	Region	Country	Project/intervention	Gender	Population	Study design
*Effectiveness studies*						
Al‐Hassan et al. (2011)	Sub‐Saharan Africa	Ghana	Grameen Ghana's microcredit scheme	Female	Women farmers	Non‐experimental design
Ambler (2018)	Sub‐Saharan Africa	Uganda	Contract farming intervention	Both	Women farmers	Experimental design
Anand et al. (2019)	South Asia	India	Self‐help programme: SHGs in the Mahila Vikas Pariyojana (MVP) 2017	Female	Women farmers	Non‐experimental design
Arslan et al. (2020)	Latin America and the Caribbean	Peru	Highlands and High Rainforest Areas Project	Both	Producer associations	Non‐experimental design
Arslan et al. (2018)	East Asia and Pacific	Philippines	Irrigated Rice Production Enhancement Project	Both	Irrigators' associations	Non‐experimental design
Bonilla et al. (2017)	Sub‐Saharan Africa	Kenya	Smallholder Dairy Commercialisation Programme	Both	Dairy farmers	Non‐experimental design
Cavatassi and Mallia (2018)	Europe and Central Asia	Tajikistan	Livestock and Pasture Development Project	Both	Rural households	Non‐experimental design
Cavatassi et al. (2019)	East Asia and the Pacific	Indonesia	Coastal Community Development Project	Both	Rural households	Non‐experimental design
Cole et al. (2020)	Sub‐Saharan Africa	Zambia	Post‐harvest fish loss reduction intervention	Both	Small‐scale fisheries (women farmers)	Non‐experimental design
Crookston et al. (2021)	Sub‐Saharan Africa	Burkina Faso	An agricultural development programme intervention package consisting of agricultural loans and services	Both	Members of savings group and their spouses	Non‐experimental design
Desai and Joshi (2013)	South Asia	India	Backward districts initiative SHG membership or participation in SEWA programmes	Female	Women farmers	Non‐experimental design
Desai (2014)	South Asia	India	Women Farmers with Global Potential initiative rural producer organisations, 2008	Female	Women farmers	Non‐experimental design
Dohmwirth (2020)	South Asia	India	Co‐operative membership	Both	Milk producers	Non‐experimental design
Ebata and Huettel (2017)	Latin America and the Caribbean	Nicaragua	Entrepreneurial practices for farmers	Both	Smallholder	Non‐experimental design
Fischer and Qaim (2012)	Sub‐Saharan Africa	Kenya	Farmer collective action	Both	small scale banana producers	Non‐experimental design
Fuller (2013)	Sub‐Saharan Africa	Congo, Rep	Food security and livelihoods project (DRCB12)	Both	Fishers and fish processors	Non‐experimental design
Garbero and Cichaibelu (2018)	Sub‐Saharan Africa	Tanzania	ASDP‐L and ASSP projects	Both	Association	Non‐experimental design
Garbero et al. (2018)	Sub‐Saharan Africa	Senegal	The Agricultural Value Chains Support Project (Projet d'Appui aux Filieres Agricole)	Both	Farmers	Non‐experimental design
Garbero and Songsermsawas (2018)	South Asia	China	Guangxi Integrated Agricultural Development Project	Both	Rural farmers	Non‐experimental design
Ghebru et al. (2019)	Sub‐Saharan Africa	Mozambique	Innovation for Agribusiness project	Both	Smallholder farmers	Non‐experimental design
Gichungi et al. (2020)	Sub‐Saharan Africa	Kenya	The African Fruit Fly Programme	Both	Mango‐ growing household	Non‐experimental design
Handschuch and Wollni (2016)	Sub‐Saharan Africa	Kenya	Kenyan Agricultural Research Institute implemented a finger millet extension programme, 2011	Both	Finger millet producers	Non‐experimental design
Hughes et al. (2018)	Sub‐Saharan Africa	Kenya	Vi Agroforestry programme	Both	Smallholder farmers	Non‐experimental design
Ishaq et al. (2016)	South Asia	Pakistan	Plan International's Pakistan project for milk marketing co‐operatives	Both	Small‐scale milk producers	Non‐experimental design
Kafle et al. (2018)	South Asia	Nepal	High Value Agriculture Project in Hill and Mountain Areas	Both	Small‐scale producers	Non‐experimental design
Kafle et al. (2021)	South Asia	Nepal	Value chain development programme for high‐value agricultural commodities	Both	Small‐scale producers	Non‐experimental design
Kamau et al. (2018)	Sub‐Saharan Africa	Kenya	Kenya Agricultural Productivity and Agribusiness Project under the subcomponent on Promotion of Indigenous Chicken Value Chain	Both	Smallholder farmers	Non‐experimental design
Knößlsdorfer et al. (2021)	Sub‐Saharan Africa	Cote d'Ivoire	Fair trade	Both	Cocoa farmers	Non‐experimental design
Kolade and Harpham (2014)	Sub‐Saharan Africa	Nigeria	Co‐operative membership	Both	Farmers	Non‐experimental design
Kumar (2021)	South Asia	India	Self help groups	Both	Not specified	Non‐experimental design
Lecoutere (2017)	Sub‐Saharan Africa	Uganda	P'KWI co‐operative membership,1993	Both	Not specified	Non‐experimental design
Leight et al. (2021)	Sub‐Saharan Africa	Burkina Faso	SELEVER	Both	Women farmers	Experimental design
Liu (2018)	South Asia	Chine	China's land co‐operative programme	Both	Members of China's land co‐operative programme	Non‐experimental design
Mabiso et al. (2018)	Sub‐Saharan Africa	Rwanda	Rural Income through Exports project	Both	Farmers	Non‐experimental design
Manza et al. (2014)	Sub‐Saharan Africa	Nigeria	Promoting Sustainable Agriculture in Southern Borno State	Both	Farmers	Non‐experimental design
Ocasio (2016)	South Asia	Bangladesh	Grameen Bank, the Bangladesh Rural Advancement Committee	Both	Rural household	Non‐experimental design
Meemken and Qaim (2018)	Sub‐Saharan Africa	Uganda	Fair trade and UTZ	Both	Coffee producers	Non‐experimental design
Meemken et al. (2019)	Sub‐Saharan Africa	Cote d'Ivoire	Fair trade and UTZ	Both	Smallholder farmers	Non‐experimental design
Minah (2022)	Sub‐Saharan Africa	Zambia	Farmer organisation	Both	Zambian household	Non‐experimental design
Miura et al. (2020)	Sub‐Saharan Africa	Zambia	Vouchers for transportation	Both	Farmers	Experimental design
Mohapatra (2016)	South Asia	India	SHG	Female	SHG members	Non‐experimental design
Mwambi et al. (2021)	Sub‐Saharan Africa	Kenya	Producer organisation	Both	Dairy smallholders	Non‐experimental design
Navarra (2018)	Sub‐Saharan Africa	Mozambique	Contract farming	Both	Female households	Non‐experimental design
Ortega et al. (2019)	Sub‐Saharan Africa	Rwanda	Crop Intensification Programme, co‐operatives membership, 2007	Both	Coffee‐ producing households	Non‐experimental design
Panda et al. (2012)	South Asia	India	Swarna Jayanti Gram Swarozgar Yojana	Female	Women in SHGs	Non‐experimental design
Pandey et al. (2020)	South Asia	India	National dairy plan (market access)	Both	Rural households engaged in dairy	Non‐experimental design
Pellegrina (2021)	South Asia	India	IIMC Microcredit Programme	Female	Women peace council members	Experimental design
Qiao (2016)	South Asia, East Asia and the Pacific	China, Sri Lanka	Organic and fair trade tea production	Both	Small‐scale tea farmers	Non‐experimental design
Quisumbing et al. (2015)	Sub‐Saharan Africa; South Asia	Mozambique; Bangladesh	HarvestPlus' Reaching End Users project	Both	Women Farmers	Experimental design
Raghunathan (2019)	South Asia	India	PRADAN's livelihoods programmes	Female	SHGs‐ women farmers	Non‐experimental design
Shah (2017)	South Asia	Nepal	The Himalayan Nettle Value Chain Intervention in Kailash Sacred Landscape	Both	Rural households	Non‐experimental design
Van Campenhout (2013)	Sub‐Saharan Africa	Uganda	ICT‐based initiatives	Both	Smallholder farmers	Non‐experimental design
van Rijsbergen (2016)	Sub‐Saharan Africa	Kenya	Fair trade	Both	Coffee co‐operatives	Non‐experimental design
Väth et al. (2014)	Sub‐Saharan Africa	Ghana	Contract farming	Both	Rural farmers	Non‐experimental design
Väth & Kirk (2019)	Sub‐Saharan Africa	Ghana	Contract farming (Outgrowers scheme)	Both	Smallholder farmers	Non‐experimental design
Pandey et al. (2021)	South Asia	India	National Dairy Plan (NPC) ‐I	Both	SHGs	Non‐experimental design
Warinda et al. (2020)	Sub‐Saharan Africa	Uganda, Kenya, Tanzania, Burundi	Comprehensive Africa Agriculture Development Programme	Both	Association Workers	Non‐experimental design
Winters et al. (2005)	East Asia and the Pacific	Indonesia	Hybrid seed contract	Both	Smallholder farmers	Non‐experimental design
Kinati et al. (2014)	Sub‐Saharan Africa	Ethiopia	Farmers Research Group	Both	Farmers	Non‐experimental design
*Process evaluations*						
Oumer (2014)	Sub‐Saharan Africa	Ethiopia	Farmers Research Group intervention	Female	Smallholder women farmers	Qualitative/Process evaluation
Altenbuchner et al. (2018)	South Asia	India	Chetna Organic	Both	Cotton growers	Qualitative/Process evaluation
Altenbuchner et al. (2021)	Latin America and the Caribbean, Sub‐Saharan Africa, South Asia	A‐ Peru, B‐Tanzania, C‐India	A‐ Bergman/Rivera, B‐ bioRe Tanzania, C‐ Chetna Organic India	Both	Cotton growers	Qualitative/Process evaluation
Mitra (2019)	South Asia	India		Both	Farmers	Qualitative/Process evaluation
Arakal and Roy (2015)	South Asia	India	Udyogini	Female	SHG members	Qualitative/Process evaluation
Asingwire (2011)	Sub‐Saharan Africa	Uganda	ICT‐based projects (Busoga Rural Open Source Development Initiative, and Women of Uganda Network)	Both	Smallholder farmers	Qualitative/Process evaluation
Bacon (2010)	Latin America and the Caribbean	Nicaragua	Unión de Cooperativas Agropecuarias, San Ramón (San Ramón Co‐operative Union) and Central de Cooperativas Cafetaleras del Norte (Organisation of Northern Coffee Co‐operatives)	Both	Smallholders	Qualitative/Process evaluation
Badejo et al. (2017)	Sub‐Saharan Africa	Nigeria	SHGs	Female	SHG members	Qualitative/Process evaluation
Coppock (2013)	Sub‐Saharan Africa	Kenya	Collective action groups for women	Female	Women farmers	Qualitative/Process evaluation
Cuellar‐Gomez (2008)	Latin America and the Caribbean	Colombia	AMUCC (Asociación de Mujeres Caficultoras Cauca)	Female	Women farmers	Qualitative/Process evaluation
Dohmwirth and Hanisch (2019)	South Asia	India	Karnataka Milk Federation	Female	Women farmers	Qualitative/Process evaluation
Dolan (2001)	Sub‐Saharan Africa	Kenya		Female	Smallholders	Qualitative/Process evaluation
Eissler (March 2020)	Sub‐Saharan Africa	Burkina Faso	Soutenir l'Exploitation Familiale pour Lancer l'Élevage des Volailles et Valoriser l'Économie Rurale project	Female	Farmers	Qualitative/Process evaluation
Ferguson and Kepe (2011)	Sub‐Saharan Africa	Uganda	Manyakabi Area Co‐operative Enterprise	Female	Farmers	Qualitative/Process evaluation
Forsythe (2016)	Sub‐Saharan Africa	Nigeria and Malawi	Cassava: Adding Value for Africa project	Female	Farmers	Qualitative/Process evaluation
Galie (2017)	Middle East and North Africa	Syria	Participatory barley breeding	Female	Farmers	Qualitative/Process evaluation
Gallardo‐Fernández and Saunders (2018)	Latin America and the Caribbean	Chile	Territorial use rights in fisheries	Female	Farmers	Qualitative/Process evaluation
Geleta et al. (2017)	Sub‐Saharan Africa	Ethiopia	Pulse Innovation Project	Female	Farmers	Qualitative/Process evaluation
Manchon (2010)	Latin America and the Caribbean	Nicaragua	FENACOOP (National Federation of Co‐operatives)	Female	Farmers	Qualitative/Process evaluation
Gurung et al. (2015)	South Asia	Nepal	International Centre for Integrated Mountain Development together with Alital Multipurpose Co‐operative Limited, Dadeldhura and the Udayapur District chapter of the Federation of Forest Users Nepal	Female	Farmers	Qualitative/Process evaluation
Hebo (2014)	Sub‐Saharan Africa	Ethiopia	Walda Hawwii Guddinnaa Omiisha Aananii (‘Hope for Development Milk Producers Co‐operative’)	Both	Milk co‐operative members	Qualitative/Process evaluation
Hutchens (2010)	South Asia	Asia region (particularly Nepal and India)	WFTO‐Asia	Female	Craft producers	Qualitative/Process evaluation
Iradukunda et al. (2019)	Sub‐Saharan Africa	Burundi	BXW Control Packages‐ SDSR Package (single disease stem removal)	Both	Farmers	Qualitative/Process evaluation
Islam (2008)	South Asia	Bangladesh	Global agro‐food system: Global commodity change and environmental government	Both	Shrimp producers	Qualitative/Process evaluation
Johnson (2015)	Sub‐Saharan Africa	Mozambique	Manica Smallholder Dairy Development Programme	Both	Smallholders	Qualitative/Process evaluation
Jones et al. (2012)	Sub‐Saharan Africa, South Asia, Latin America and the Caribbean	Kenya, Tanzania, Uganda, India, Nepal, Nicaragua, and Mexico	Action Research Women in Informal Employment: Globalising and Organising– Fair Trade and Co‐operatives Membership	Female	Farmers	Qualitative/Process evaluation
Kasente (2012)	Sub‐Saharan Africa	Uganda	Fair trade and organic certification	Both	Farmers	Qualitative/Process evaluation
Keelson and Nanekum (2021)	Sub‐Saharan Africa	Ghana	Marketing Mix: community's trader's association	Female	Smallholder farmers	Qualitative/Process evaluation
Kent (2018)	Sub‐Saharan Africa	Ghana	Organic certification scheme	Female	Farmers	Qualitative/Process evaluation
Khatun et al. (2020)	Sub‐Saharan Africa	Ghana	Roundtable on sustainable palm oil	Both	Smallholder farmers	Qualitative/Process evaluation
Kwaramba et al. (2020)	Sub‐Saharan Africa	Zimbabwe	Agricultural Policy Research in Africa Workstream	Female	Farmers	Qualitative/Process evaluation
LeBaron (2020)	Sub‐Saharan Africa	Ghana	Primary data set produced through the Global Business of Forced Labour project	Female	Farmers	Qualitative/Process evaluation
Le Mare (2012)	South Asia	Bangladesh	Southern Fair Trade Enterprises	Female	Farmers	Qualitative/Process evaluation
Loconto (2011)	Sub‐Saharan Africa	Tanzania	Certification in tea production (fair trade, sustainable, organic)	Both	Farmers	Qualitative/Process evaluation
Lukuyu et al. (2020)	Sub‐Saharan Africa	Uganda	Planted forages in piggery systems	Both	Smallholder farmers	Qualitative/Process evaluation
Lyon (2008)	Latin America and the Caribbean	Guatemala	The Guatemalan National Coffee Association, Anacafé: Fair‐ trade coffee certification and coffee co‐operative	Female	Farmers	Qualitative/Process evaluation
Lyon et al. (2019)	Latin America and the Caribbean	Mexico	Café de Oro microbatch coffee programme (co‐operative)	Female	Farmers	Qualitative/Process evaluation
Makita (2009)	South Asia	Bangladesh	Two income‐generating programmes in poultry rearing and sericulture (silkworm rearing) supported by the Bangladeshi NGO – The Institute of Integrated Rural Development	Female	Farmers	Qualitative/Process evaluation
Matenga (2017)	Sub‐Saharan Africa	Zambia	Magobbo Outgrower Sugarcane Scheme	Both	Farmers	Qualitative/Process evaluation
Okiror et al. (2021)	Sub‐Saharan Africa	Uganda	IITA‐ Nitrogen‐to‐Africa (N2Africa) project	Female	Smallholder farmers	Qualitative/Process evaluation
Ortiz‐Miranda et al. (2015)	Latin America and the Caribbean	Guatemala	FT certification schemes through civil association‐ Guaya'b and CODECH (Coordinating Committee of Organisations for the Development of Concepción Huista)	Both	Coffee	Qualitative/Process Evaluation
Othman et al. (2021)	Sub‐Saharan Africa	Zanzibar	Farming groups	Female	Farmers	Qualitative/Process evaluation
Oumer et al. (2014)	Sub‐Saharan Africa	Ethiopia	Farmers Research Group established by the Ethiopian Institute of Agricultural Research, Holetta Agricultural Research Centre:	Female	Farmers	Qualitative/Process evaluation
Pankaj (2020)	South Asia	India	Jeevika (Bihar Rural Livelihood Promotion Society): Adarsh Mahila Cluster Level Federation: self help groups	Female	Farmers	Qualitative/Process evaluation
Papa et al. (2000)	South Asia	India	India's National Dairy Development Board launched the Co‐operative Development programme	Female	Farmers	Qualitative/Process evaluation
Perry (2020)	Middle East and North Africa	Morocco	Argan oil co‐operatives ‐ fair trade	Female	Farmers	Qualitative/Process evaluation
Pineda (2019)	Latin America and the Caribbean	Colombia	National Federation of Coffee Growers: La Familia Cafetera (the coffee producing‐family): Coffee certification	Female	Coffee producers	Qualitative/Process evaluation
Porter et al. (2014)	Sub‐Saharan Africa	Nigeria	Fadama user groups	Female	Farmers	Qualitative/Process evaluation
Ragasa et al. (2021)	Sub‐Saharan Africa	Malawi	Agricultural Technical Vocational Education and Training for Women (ATVET4Women) Programme	Female	Farmers	Qualitative/Process evaluation
Raynolds (2002)	Latin America and the Caribbean	Dominican Republic	Contract farming	Female	Farmers	Qualitative/Process evaluation
Rice et al. (2019)	East Asia and the Pacific	Solomon Islands	Sustainable Farming for Nutrition and Income initiative	Female	Farmers	Qualitative/Process evaluation
Utting‐Chamorro (2005)	Latin America and the Caribbean	Nicaragua	Larger producer organisations (CECOCAFEN and SOPPEXCCA): fair trade	Both	Farmers	Qualitative/Process evaluation
Venema and van Eijk (2004)	Sub‐Saharan Africa	Senegal	(1) Bok Xalaat women's group; (2) Bok Djoubo women's group	Female	Farmers	Qualitative/Process evaluation
Ya‐Bititi (2015)	Sub‐Saharan Africa	Rwanda	Coffee washing stations ‐ Karaba coffee co‐operative	Female	Coffee producers	Qualitative/Process evaluation
Zulu (2020)	Sub‐Saharan Africa	Malawi, Ghana	Africa RISING agriculture project	Female	Farmers	Qualitative/Process evaluation
*Mixed methods*						
Namayengo (2017)	Sub‐Saharan Africa	Uganda	BRAC microfinance programme	Female	Women	Mixed methods
Holmes and Imai (2019)	South Asia	Sri Lanka	Fair trade certification	Female	Small‐scale producers	Mixed methods
Mojo (2017)	Sub‐Saharan Africa	Ethiopia	Agricultural co‐operatives	Both	Coffee farmers‐ both	Mixed methods
Quisumbing et al. (2013)	South Asia	Bangladesh	Dairy Value Chain Project	Both	Smallholder farmers	Mixed methods

### 5.3 Critical appraisal of the studies

#### 5.3.1 Effectiveness studies

The critical appraisal tool assess the confidence in findings of the studies based on key features including study design, attrition, power calculation and reporting. The tool contains critical dimensions, each of which is marked as ‘high confidence’, ‘medium confidence’, and ‘low confidence’. The overall score uses the ‘weakest link in the chain’ (or maxi‐min) principle. Hence, the confidence in study findings can only be as high as the lowest rating given to the six critical items in effectiveness studies.

**Figure 10:**
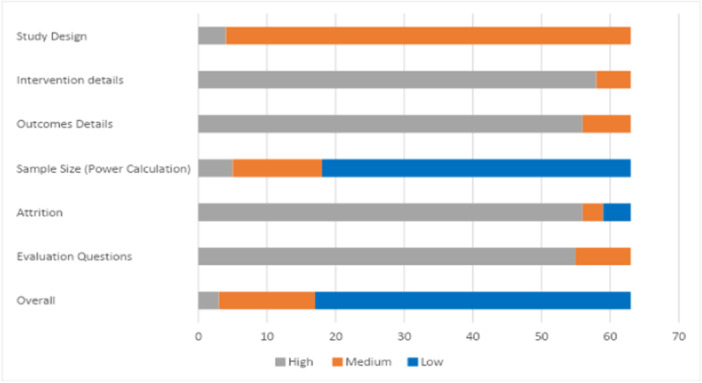
Critical appraisal of effectiveness studies.

In this review, most of the effectiveness studies included mixed‐methods studies (*n* = 46; 73%) are rated as having low confidence in their study findings, with only 22% (14 studies) rated as medium confidence, and 5% (three studies) rated as high confidence (see Figure 10). The studies score highly in terms of framing the evaluation questions, detailing the intervention and the outcomes, and attrition of study participants.

Few studies are randomised controlled trials due to the difficulty of setting up experiments to evaluate these types of interventions; the vast majority use quasi‐experimental methods such as matching, difference in difference, and instrumental variables. These types of studies often rely on existing data and do not perform power calculations beforehand to define the optimum sample size. Because of the predominance of quasi‐experimental studies and the rarity of power calculations, the included studies obtain an average score of low confidence.

#### 5.3.2 Process evaluations

**Figure 11:**
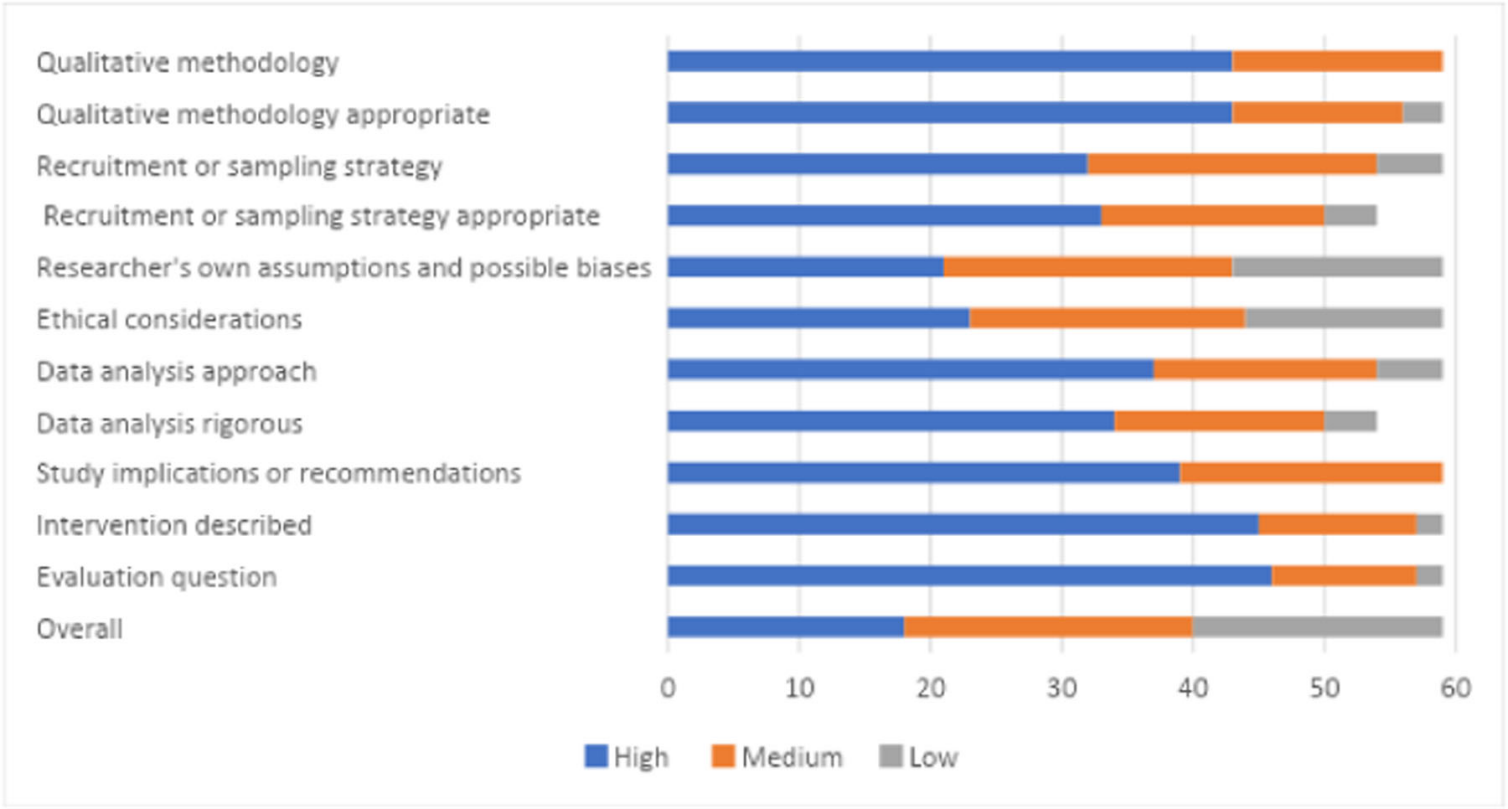
Critical appraisal of process evaluation.

In this review, there are a total of 55 process evaluations and 4 mixed method studies. According to our assessment tool, approximately 30% (18 studies) were rated as high‐confidence in study findings, 37% (22 studies) were rated as medium confidence, and 32% (19 studies) were rated as low confidence (see Figure 11). The assessment tools considered all the items critical items and it follows the principle of the weakest chain principle.

The studies score highly in terms of framing the evaluation questions, detailing the intervention and the outcomes, and mentioning the qualitative methodology and recruitment or sampling strategies.

The predominance of low rating overall is due to lower rating lack of reporting on researcher's assumption and ethical consideration.

## 6 SYNTHESIS OF THE FINDINGS (QUANTITATIVE AND QUALITATIVE)

### 6.1 Quantitative synthesis: Meta‐analysis findings

We were able to calculate 1700 effect sizes from the included studies. These are presented as SMDs (g) alongside 95% confidence intervals (CIs). To undertake meta‐analysis, the first step was to categorise outcomes across studies. Table 7 presents the outcome domains and subdomains incorporated in the synthesis.

**Table 7:** Outcome domains and subdomains.

Outcome domain	Outcome subdomain
1. Access and knowledge	1.1 Knowledge
	1.2 Gender attitudes
	1.3 Training
	1.4 Credit
	1.5 Bank account
	1.6 Collective (e.g., membership of farmer group)
2. Inputs	2.1 Land
	2.2 Labour inputs
	2.3 Input costs
	2.4 Other improved inputs (including technology adoption)
	2.5 Employment
3. Market outcomes	3.1 Bargaining
	3.2 Prices
	3.3 Transaction costs
	3.4 Quality standards
	3.5 Other market access
	3.6 Sustainable income (e.g., profits from single crop and selling)
4. Economic outcomes	4.1 Income and expenditure (outcomes related to food expenditure, nutrition expenditure, and branded food expenditure)
	4.2 Productivity
	4.3 Assets ownership and value of the assets
	4.4 Investment
	4.5 Savings
5. Time use	5.1 Workload
	5.2 Leisure
6. Empowerment	6.1 Women's empowerment
	6.2 Women's decision‐making power (e.g., agricultural production, income, or household food consumption; value chain activities)
	6.3 Women's participation
	6.4 Paid women's labour
	6.5 Women's mobility (e.g., to the spaces considered as ‘male’, the environment restricting mobility)
	6.6 Women's leadership

#### 6.1.1 Meta‐analysis by outcome domains

**Figure 12:**
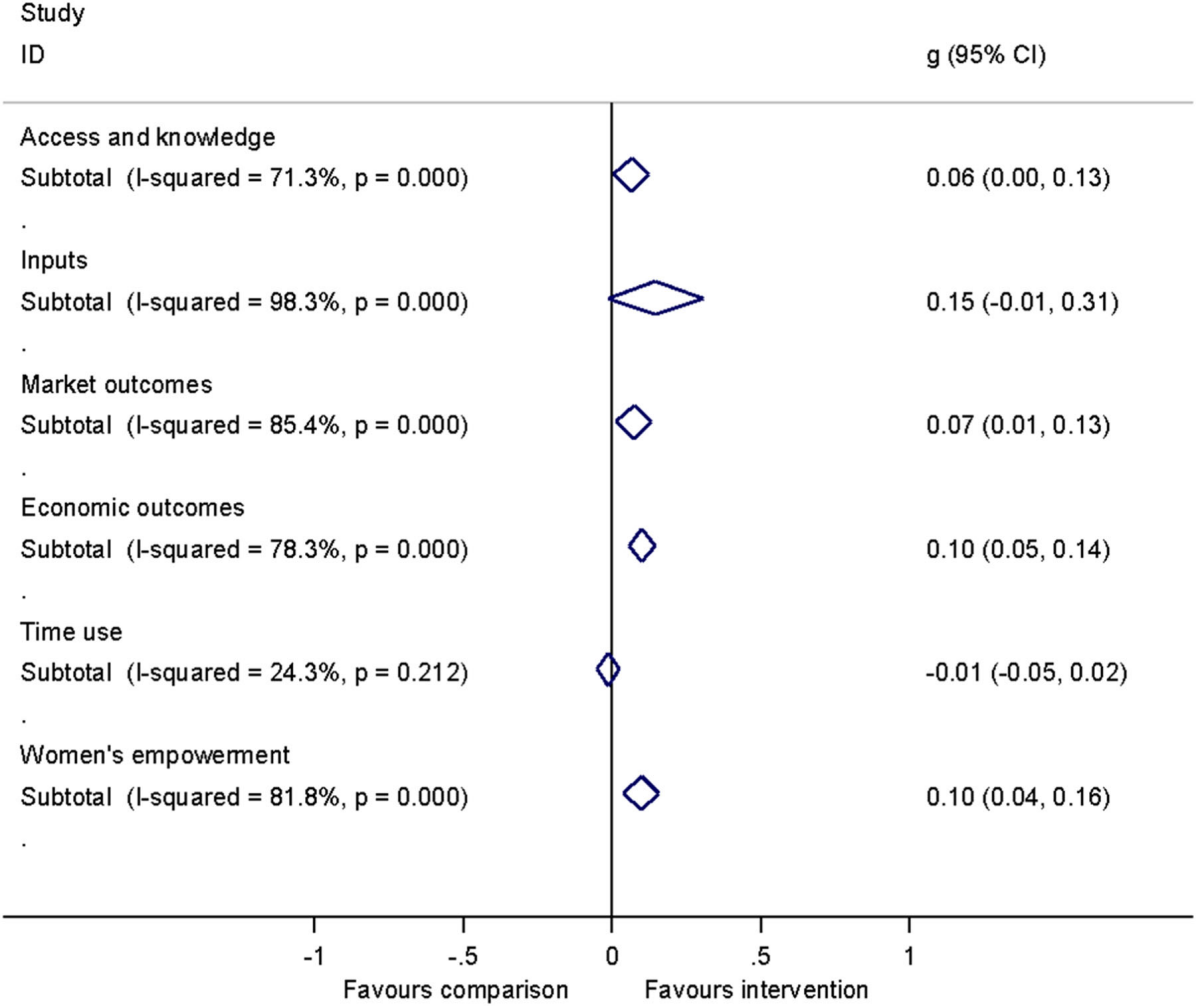
Overall meta‐analysis findings by outcome.

Average impacts of the interventions were estimated through meta‐analyses, firstly, by outcome domain and subsequently by outcome subdomain. To generate forest plots at the outcome level, we first calculated a single synthetic effect size for each study by taking the weighted average of effects across subcategories by outcome, as indicated in the methods section. The overall forest plot for outcome domains is shown in Figure 12.

The findings indicate that the outcomes in nearly all cases favour the intervention, meaning that a positive effect is estimated; the magnitude of effects are of small to medium size. In spite of the small effect sizes and owing to the large numbers of studies we were able to find and incorporate in the analysis, most effects are statistically significant (i.e., the 95% CIs do not overlap the null effect line). Figure 13 presents individual forest plots for the outcomes shown in summary in Figure 12, indicating the heterogeneity in effects across the contributing studies.

Figure 13Meta‐analysis by outcome domain, showing individual studies.

**Figure:**
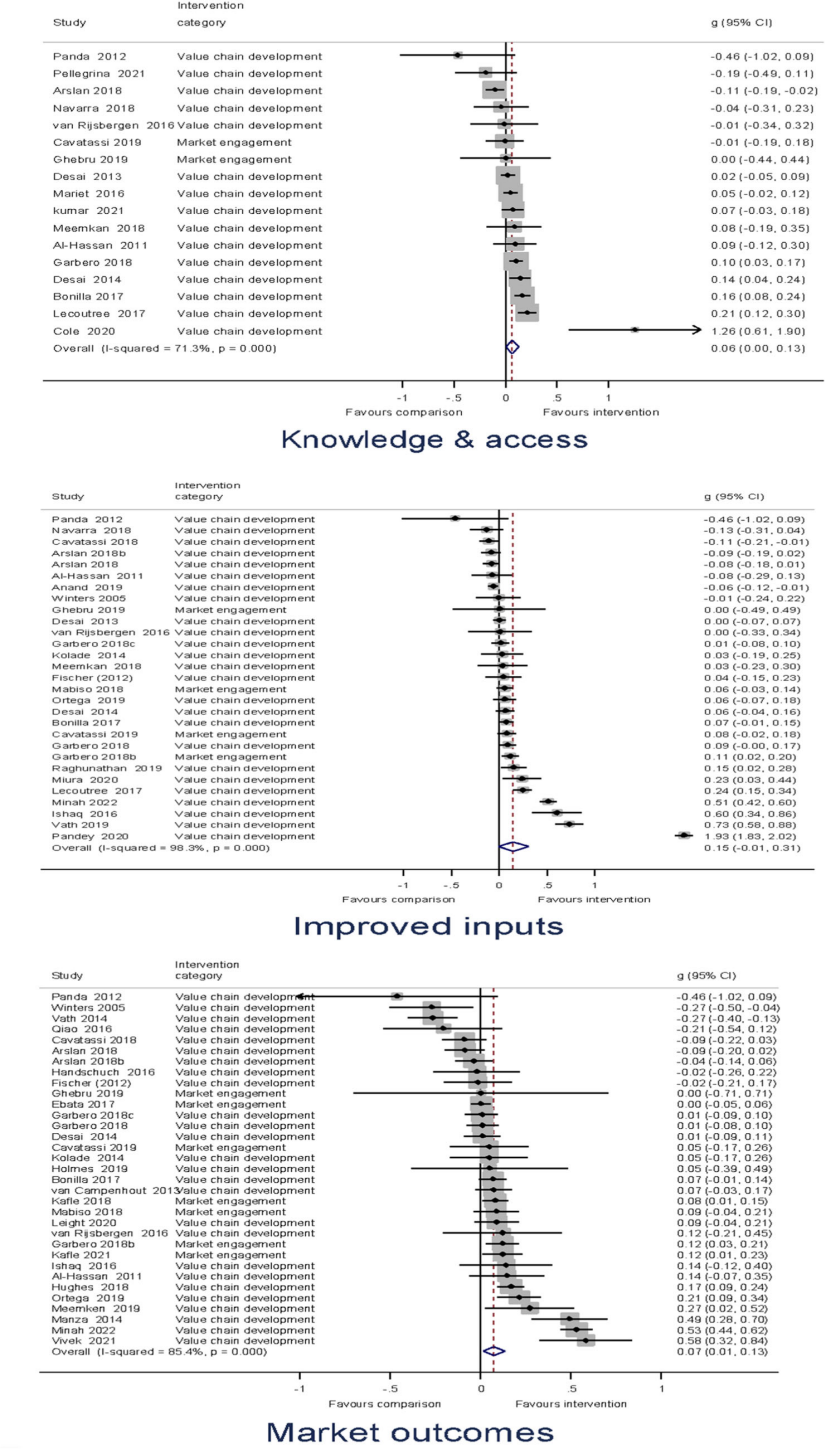

**Figure:**
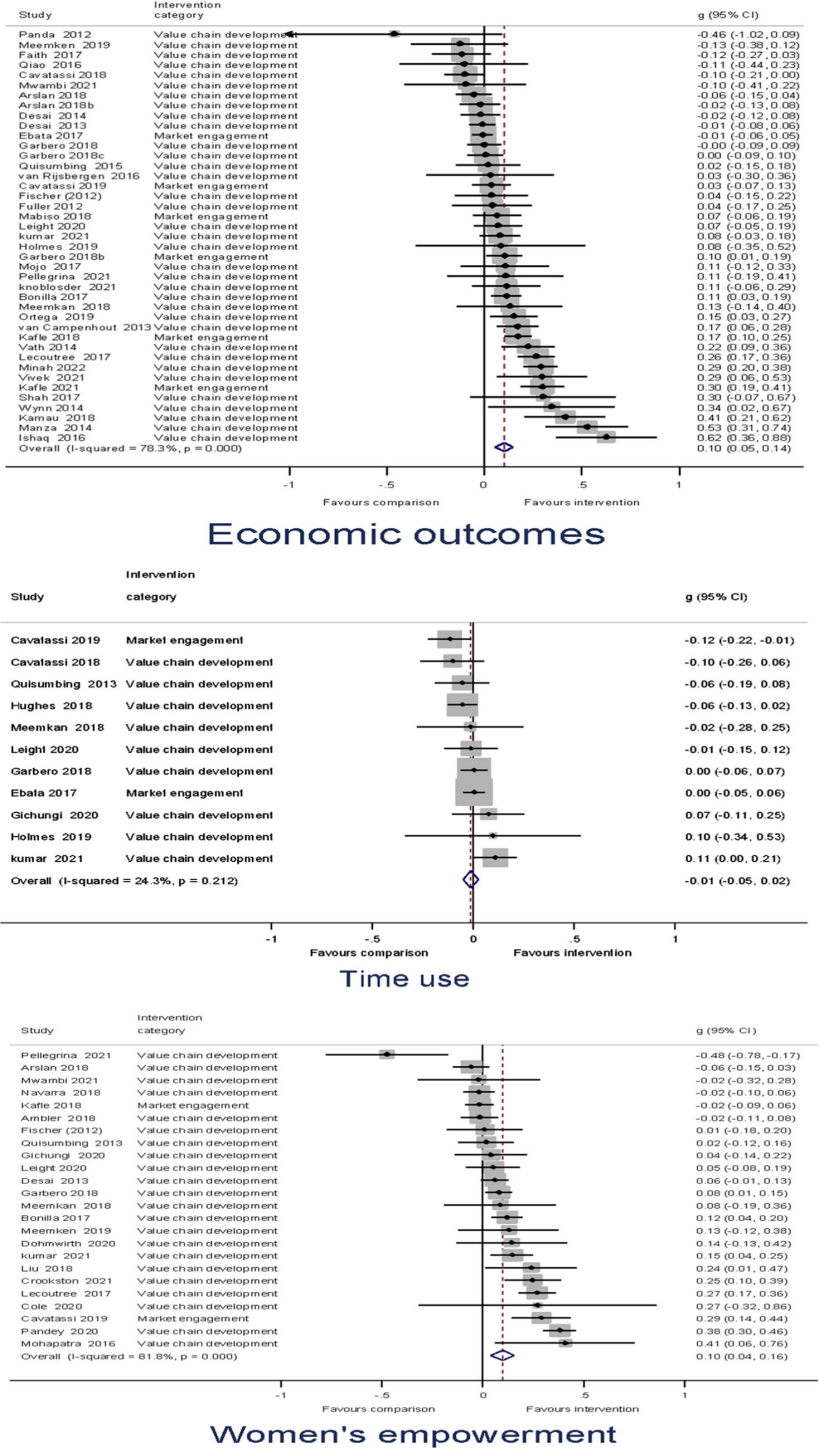

Thus, for knowledge and access, evidence from 17 studies provides the estimated pooled effect of 0.06 standard deviations improvement in the outcome (SMD = 0.06, 95% CI = 0.00, 0.13) with substantial between‐study heterogeneity (*I* ^2^ = 0.71%, Tau^2^ = 0.09). Most of the remaining findings suggest medium‐size effects: for market outcomes (SMD = 0.07, 95% CI = 0.01, 0.13; *I* ^2^ = 85%, Tau^2^ = 0.02; 33 estimates); economic outcomes (SMD = 0.10, 95% CI = 0.05, 0.14; *I* ^2^ = 78%, Tau^2^ = 0.01; 41 estimates); and empowerment outcomes (SMD = 0.10, 95% CI = 0.04, 0.16; *I* ^2^ = 82%, Tau^2^ = 0.02; 24 estimates). The effect for improved inputs, where the pooled effect is estimated of 0.15 standard deviations improvement in the outcome (SMD = 0.15, 95% CI = −0.01, 0.31), is not statistically significant and estimated with substantial estimated between‐study heterogeneity (*I* ^2^ = 98%, Tau^2^ = 0.18; 29 estimates).

These results mean that value chain interventions are on the whole successful at improving economic and empowerment outcomes. All outcome domains are positively affected by the interventions, the average impacts are not large, and the impact on empowerment outcomes is significant in comparison to outcomes observed in other domains. However, these estimates are grouping together very different outcomes and projects that differ greatly in characteristics and implementation; they are therefore concealing a large amount of heterogeneity. A better understanding of the effectiveness of the interventions requires greater differentiation of the outcomes and of the interventions.

It is also noteworthy that, while effects on outcomes are positive in most cases, there is no beneficial effect on time use outcomes – that is, interventions do not act to reduce women's burden of working time or increase their available time for leisure. Indeed, effects were time‐neutral, as there were neither significant reductions nor significant increases in time spent on agriculture and household chores (SMD = −0.01, 95% CI = −0.05, 0.02) with very little heterogeneity in findings across the studies (*I* ^2^ = 24%, Tau^2^ = 0.00; 11 estimates).

#### 6.1.2 Meta‐analysis by outcome subdomains

**Figure 14:**
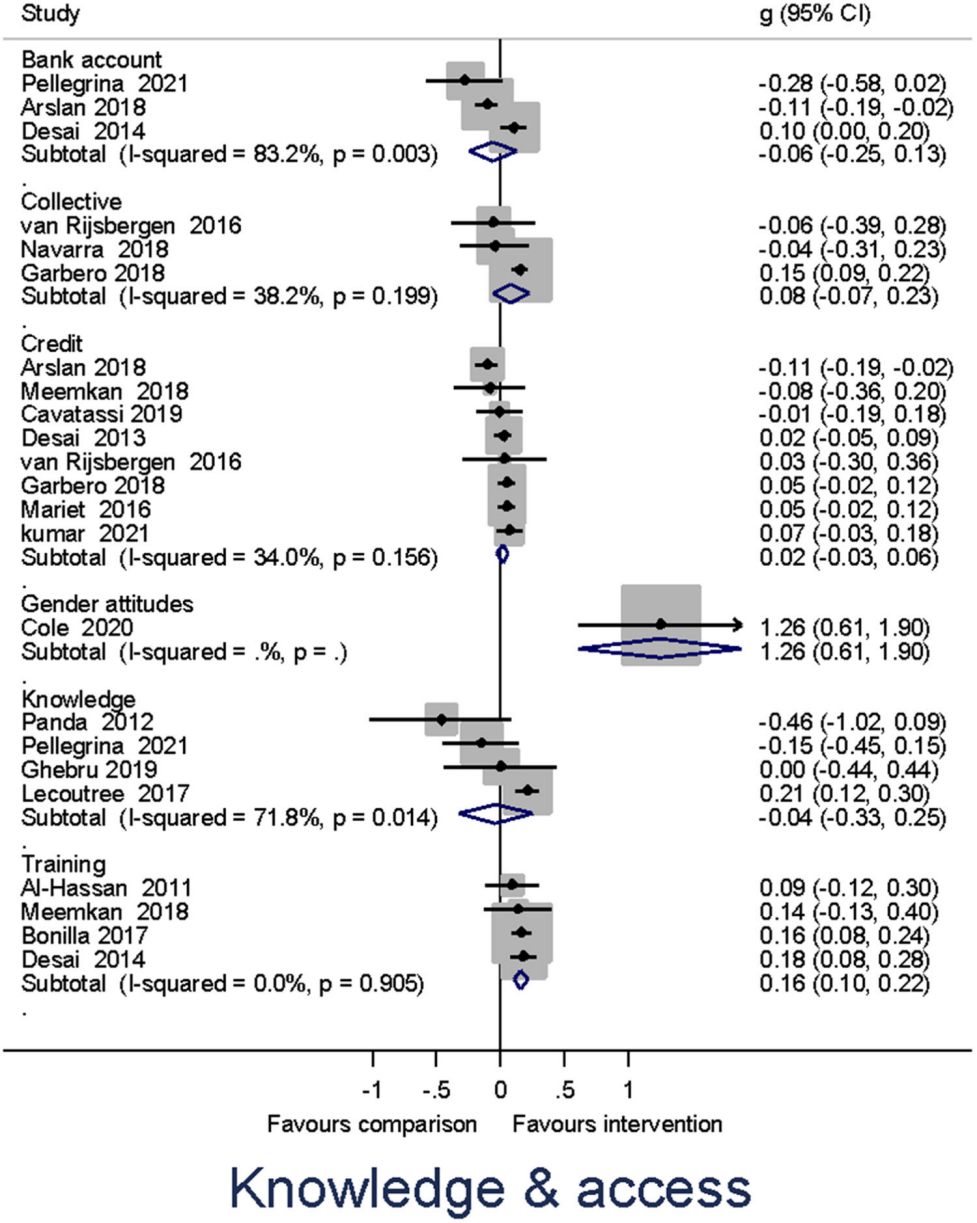
Meta‐analysis by knowledge and access outcome subdomains.

Further analysis revealed some potentially important differences in findings by outcome subdomains. Taking the knowledge and access outcome group (Figure 14), the meta‐analysis found a moderate and significant increase in study participants trained (SMD = 0.16, 95% CI = 0.10, 0.22; *I* ^2^ = 0%, Tau^2^ = 0.00; 4 estimates), but there were smaller or null effects for the other outcomes, such as knowledge (SMD = −0.036, 95% CI = −0.326, 0.253; *I* ^2^ = 72%, Tau^2^ = 0.06; 4 estimates), access to credit (SMD = 0.016, 95% CI = −0.029, 0.061; *I* ^2^ = 34%, Tau^2^ = 0.00; 8 estimates) or a bank account (SMD = −0.060, 95% CI = −0.247, 0.127; *I* ^2^ = 83%, Tau^2^ = 0.02; 3 estimates), or a farmer collective (SMD = 0.081, 95% CI = −0.066, 0.227; *I* ^2^ = 38%, Tau^2^ = 0.01; 3 estimates).

However, one study of gender transformative programming (Cole et al., 2020) found a large impact on gender attitudes. Overall, however, the studies are too few and the between‐study heterogeneity is too high to conclude with sufficient confidence about impacts on knowledge and access outcomes.

**Figure 15:**
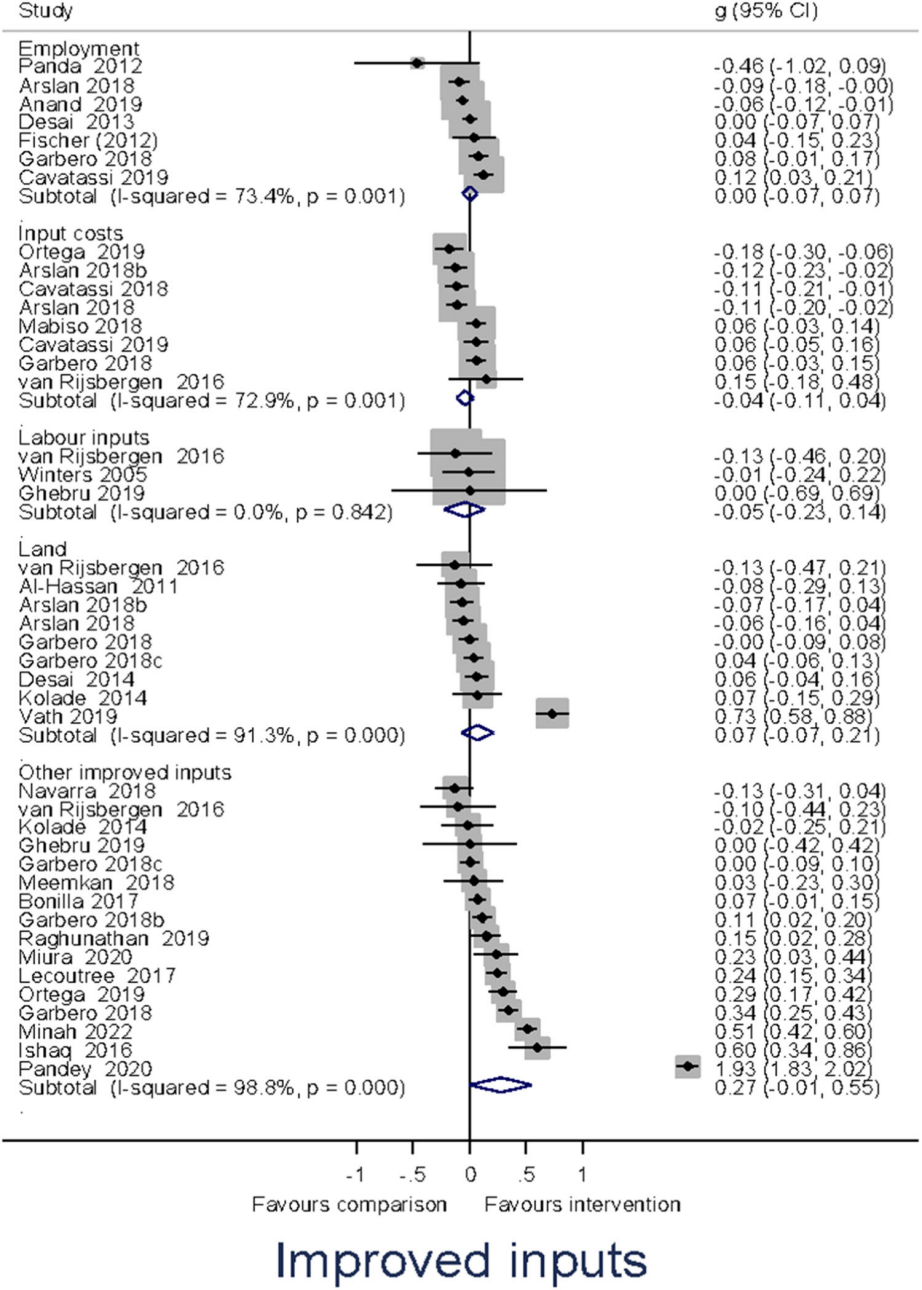
Meta‐analysis by input subdomains.

The meta‐analysis for input subdomains (Figure 15) suggested that the substantial heterogeneity in findings at the outcome domain level were indeed due to differences across the subdomains. For example, while other improved inputs (which include adoption of improved technologies) appear to have increased under the interventions (SMD = 0.271, 95% CI = −0.008, 0.550), the findings are not statistically significant at 5% confidence, and heterogeneity is very high (*I* ^2^ = 99%, Tau^2^ = 0.31; 16 estimates).

Similarly, effects on access to land were small and inconsistent (SMD = 0.068, 95% CI = −0.074, 0.210; *I* ^2^ = 91%, Tau^2^ = 0.04; 9 estimates), as were effects on reduction input costs (SMD = −0.039, 95% CI = −0.113, 0.035; *I* ^2^ = 73%, Tau^2^ = 0.01; 8 estimates). In the case of labour inputs (SMD = −0.045, 95% CI = −0.228, 0.138; *I* ^2^ = 0%, Tau^2^ = 0.00; 3 estimates) and employment (SMD = 0.003, 95% CI = −0.068, 0.074; *I* ^2^ = 73%, Tau^2^ = 0.01; 7 estimates), the findings suggested no effects, which mirrors the estimated null effect on time use. In other words, it does not appear that farm households typically draw on more labour inputs, either internally through increasing women's work or externally by hiring labour, to support the changes in practices being promoted.

**Figure 16:**
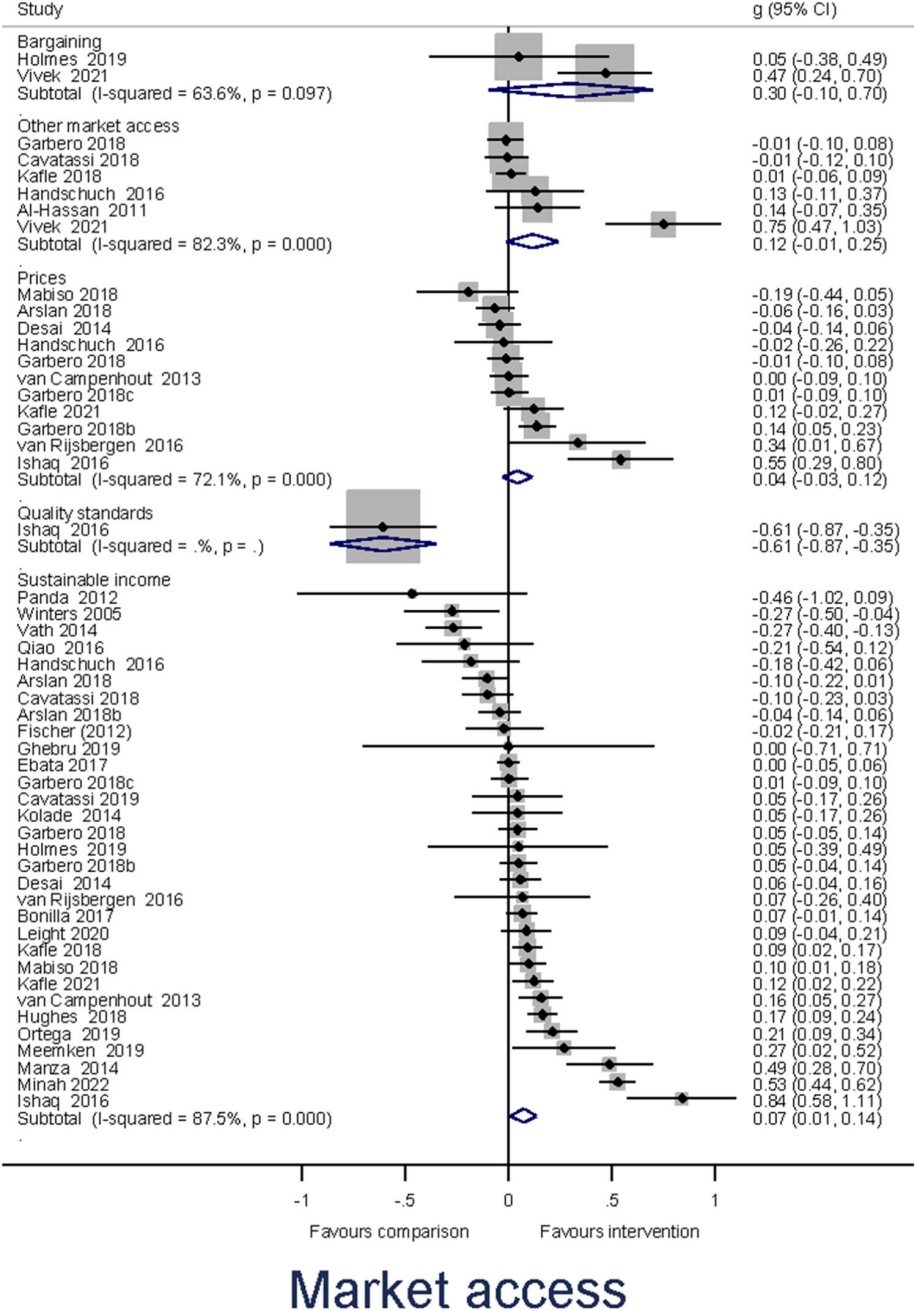
Meta‐analysis by market outcome subdomains.

The findings for market outcome subdomains (Figure 16) suggested improvements, on average, in market access (SMD = 0.118, 95% CI = −0.011, 0.247; *I* ^2^ = 82%, Tau^2^ = 0.02; 6 estimates) and access to more sustainable incomes (SMD = 0.073, 95% CI = 0.009, 0.137; *I* ^2^ = 88%, Tau^2^ = 0.02; 31 estimates). However, the effect of interventions on more favourable prices were small and inconsistent (SMD = 0.045, 95% CI = −0.029, 0.119; *I* ^2^ = 72%, Tau^2^ = 0.01; 11 estimates). Only three studies measured changes in bargaining or quality standards.

**Figure 17:**
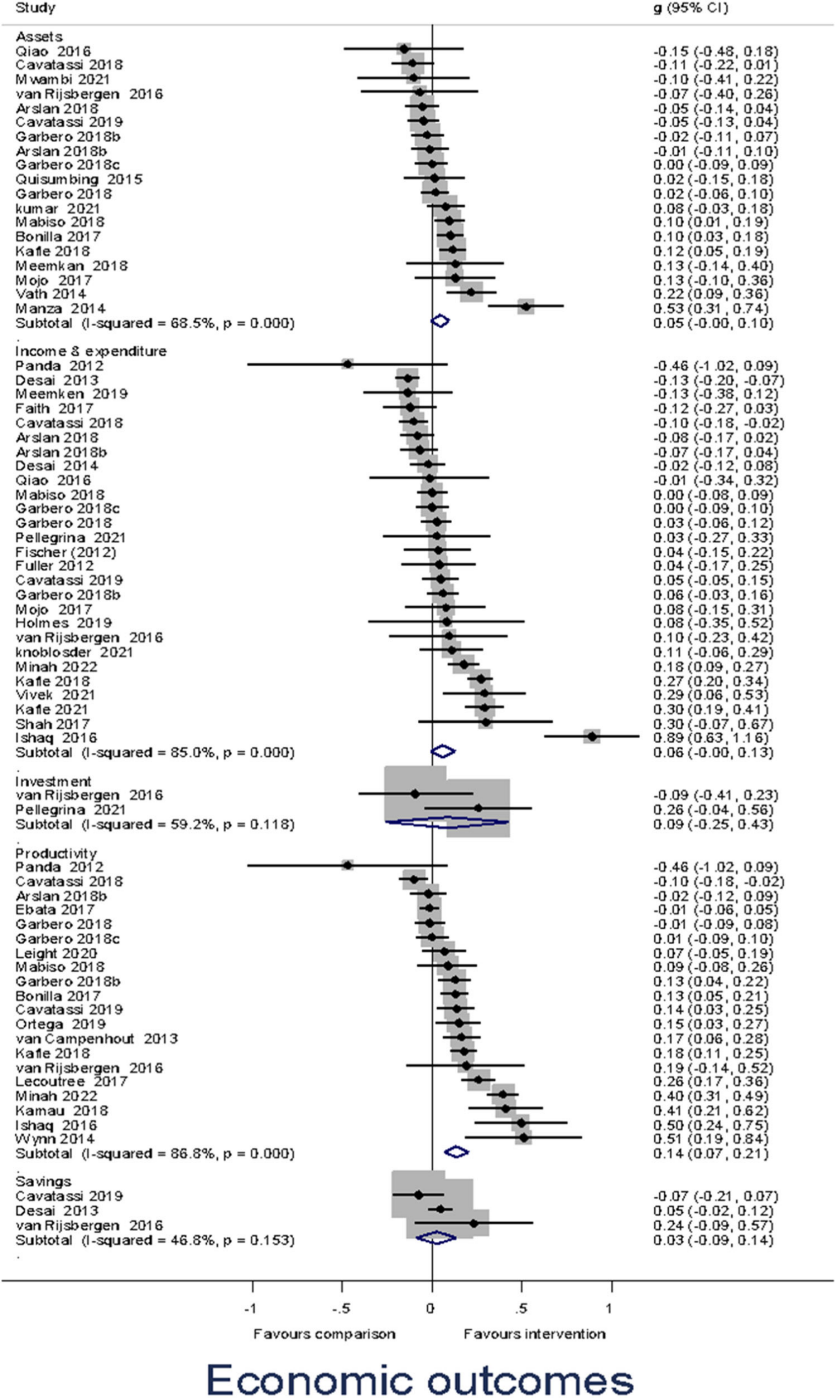
Meta‐analysis by economic outcome subdomains.

The pooled intervention effects for economic outcome subdomains were also estimated (Figure 17). The meta‐analysis indicated that the interventions improved productivity by a medium and highly significant effect (SMD = 0.137, 95% CI = 0.069, 0.205; *I* ^2^ = 87%, Tau^2^ = 0.02; 20 estimates). The interventions also tended to improve income and expenditure itself, albeit with high heterogeneity (SMD = 0.063, 95% CI = −0.002, 0.129; *I* ^2^ = 85%, Tau^2^ = 0.01; 27 estimates). The effects on asset holdings (SMD = 0.047, 95% CI = −0.002, 0.097; *I* ^2^ = 67%, Tau^2^ = 0.01; 19 estimates) and savings (SMD = 0.027, 95% CI = −0.029, 0.119; *I* ^2^ = 47%, Tau^2^ = 0.05; 3 estimates) were on average small and inconsistent.

**Figure 18:**
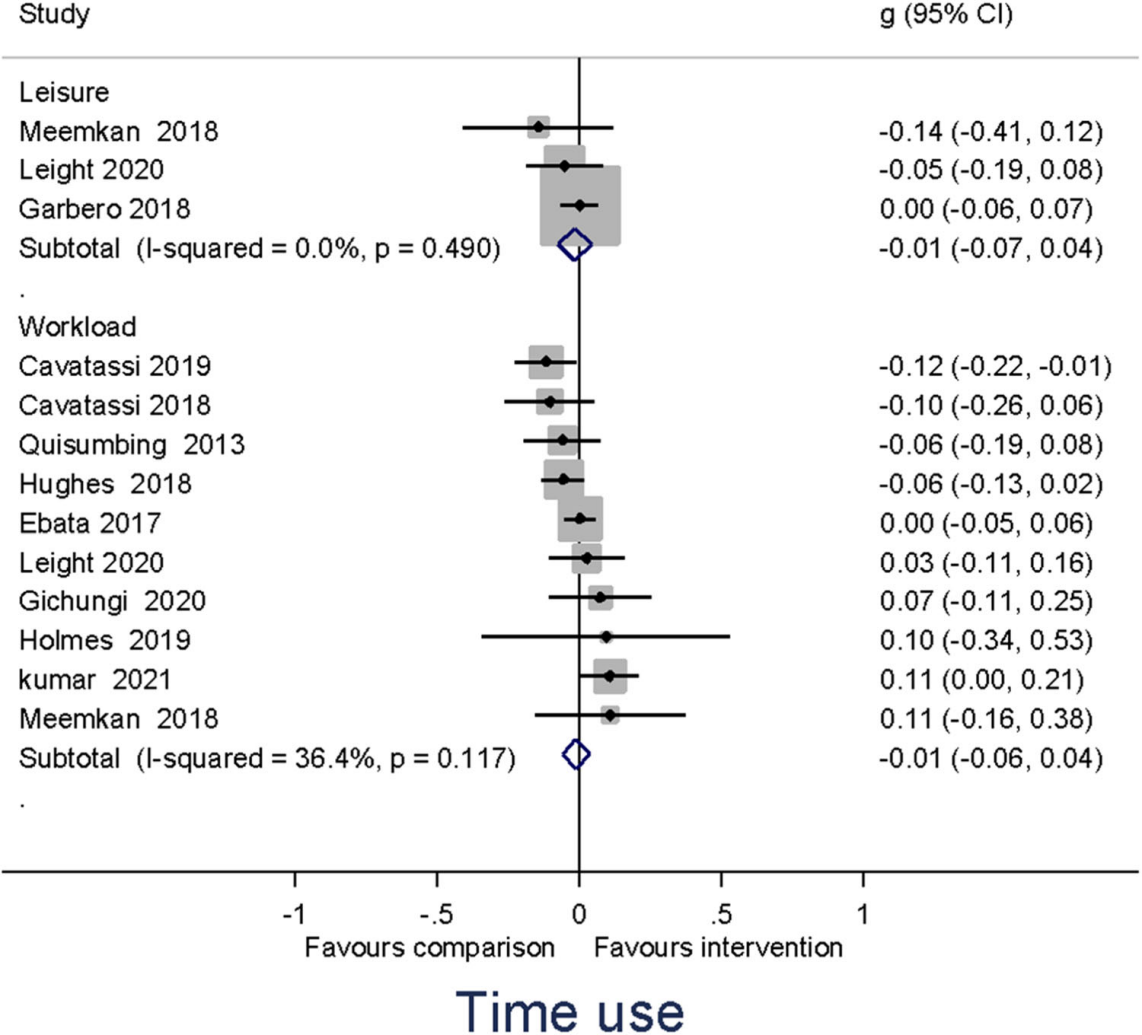
Meta‐analysis by time use subdomains.

Finally, we turn to the domains most closely related to women's empowerment. Figure 18 presents the time use subdomains, which indicate no effects, on average, on leisure (SMD = −0.014, 95% CI = −0.073, 0.044; *I* ^2^ = 0%, Tau^2^ = 0.00; 3 estimates) or workloads (SMD = −0.011, 95% CI = −0.059, 0.037, *I* ^2^ = 36%, Tau^2^ = 0.00; 10 estimates). There is small heterogeneity in effects on workloads, as a single study did find a moderate‐sized and significant increase in workload for women (Cavatassi et al., 2019) as a result of a fishery community development project.

**Figure 19:**
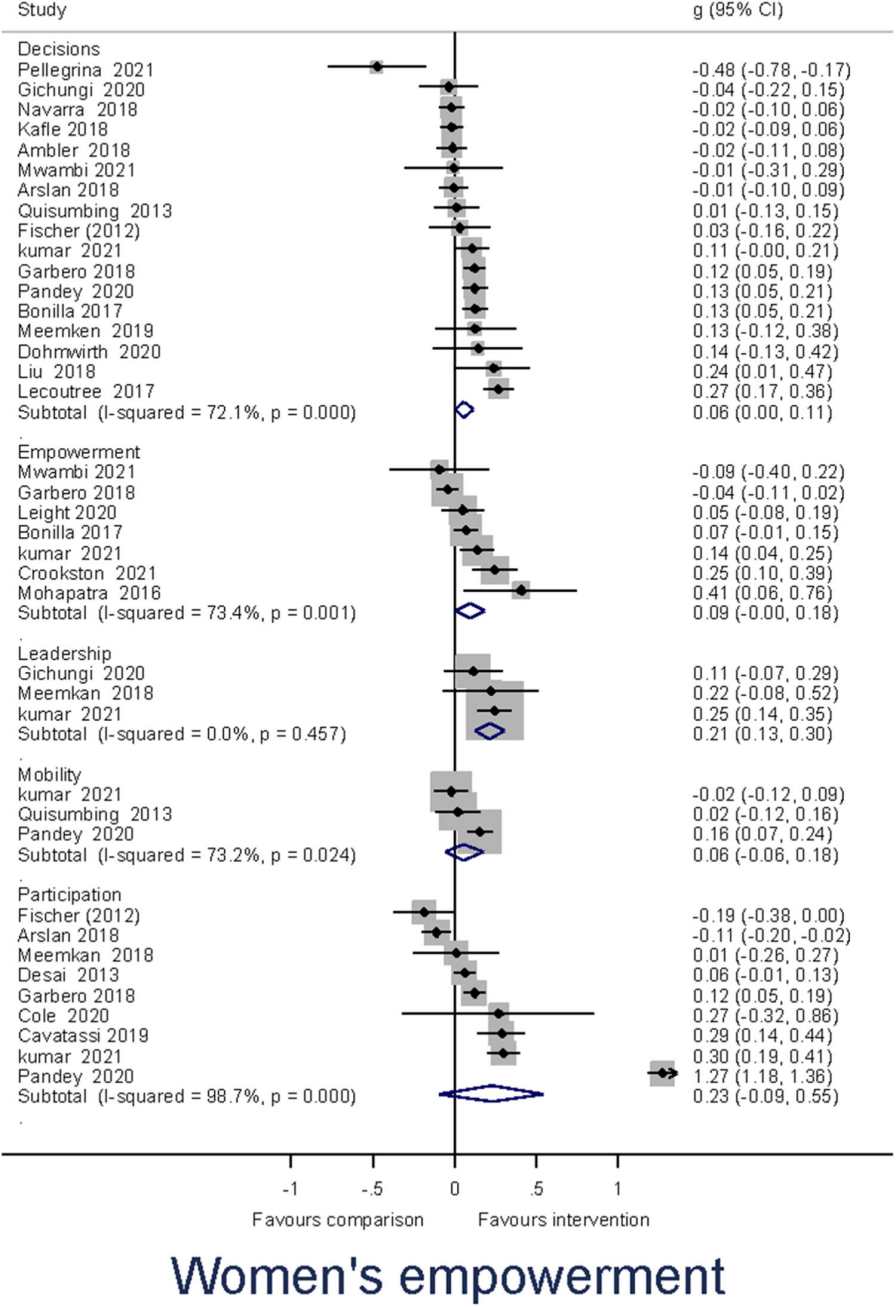
Impact of interventions on women's empowerment.

However, the intervention effects on other measures of women's empowerment were more positive, although they tended to be of small magnitude (Figure 19). Thus, there were positive intervention effects on women's decision‐making (SMD = 0.059, 95% CI = 0.005, 0.114, *I* ^2^ = 72%, Tau^2^ = 0.01; 17 estimates) and empowerment (e.g., money management) (SMD = 0.091, 95% CI = −0.001, 0.183, *I* ^2^ = 73%, Tau^2^ = 0.01; 7 estimates).

While the meta‐analysis of the few studies measuring women's leadership also suggested moderately positive impacts (SMD = 0.213, 95% CI = 0.126, 0.300, *I* ^2^ = 0%, Tau^2^ = 0.00; 3 estimates), the story was less clear for participation, where the average effect was of a similar magnitude but not statistically significant due to great heterogeneity in the estimates (SMD = 0.227, 95% CI = −0.094, 0.548, *I* ^2^ = 99%, Tau^2^ = 0.22; 9 estimates). The meta‐analysis suggested there may be small effects on mobility on average (SMD = 0.058, 95% CI = −0.060, 0.177, *I* ^2^ = 73%, Tau^2^ = 0.01; 3 estimates). These were due to the positive effect found in a single study of the Indian NDP‐I programme on needing permission to visit friends, doctors, attend village meetings or purchase household items, but the findings were not consistent across other studies.

#### 6.1.3 Publication bias assessment

**Figure 20:**
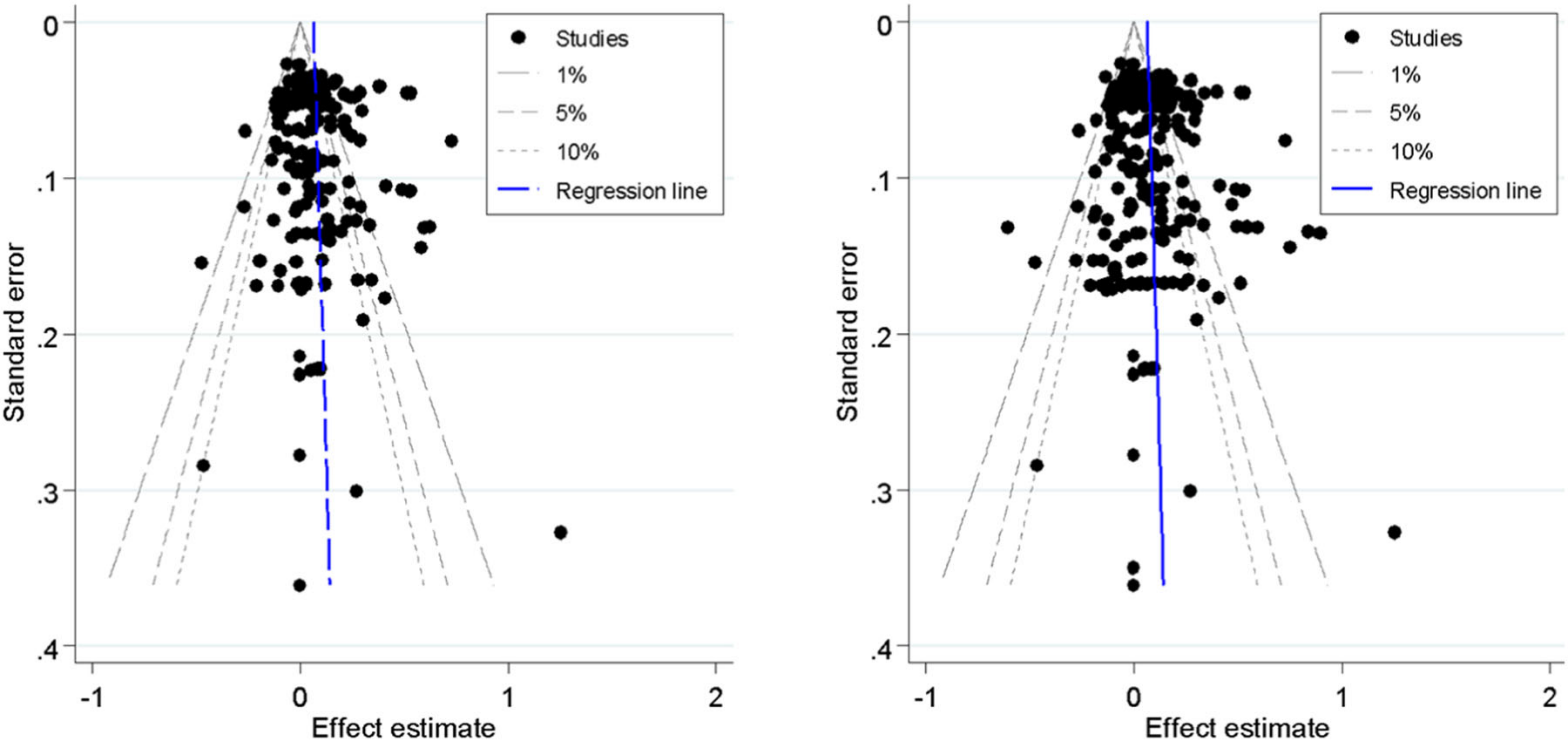
Funnel graphs by outcome domain and outcome subdomain.

We assessed whether there might be small study effects indicative of publication bias, which would inter alia, because the estimated pooled effects tend to be upwards‐biased. The publication bias assessments, carried out by outcome domain and subdomain, did not suggest that small study effects were present for the studies incorporated in this review (Figure 20). The funnel graphs were symmetrical at all values of the standard error, and the Egger regression lines were estimated to pass through, or near to, the origin for outcome domain (*p* > 0.66) and subdomain (*p* > 0.61).

#### 6.1.4 Moderator analysis

In the previous section we analysed the impacts of value chain interventions implemented in different countries and by different projects. Results, however, may differ in different contexts and by type of intervention. In this section we discuss the results of a moderator analysis (Supporting Information: Appendix F) in which we disaggregate the impact observed in the previous section by geographic area of implementation, by project type, and by whether the intervention was targeted at women.

In our first moderator analysis we consider five broad geographic areas: East Asia and the Pacific, Europe and Central Asia, Latin America and the Caribbean, South Asia, and Sub‐Saharan Africa. The majority of available studies come from South Asia and Sub‐Saharan Africa, while few report results from Europe and Latin America. The overall effects in South Asia and Sub‐Saharan Africa are therefore estimated more precisely than others, and the results of our cross‐area comparisons should be interpreted with some caution.

Only those projects implemented in South Asia and Sub‐Saharan Africa show consistently positive results across all outcome categories. Results appear to be higher on average in Sub‐Saharan Africa than in South Asia with respect to knowledge, input use, and market access. Overall impacts on empowerment outcomes and economic outcomes, however, are nearly identical in South Asia and Sub‐Saharan Africa.

In our second moderator analysis, we consider separately targeted and untargeted interventions. Some interventions were specifically designed to provide services to women and to address constraints preventing them from accessing profitable markets. We compare the results of these interventions to those obtained by value chain interventions that were not specifically targeted to women.

Targeted and untargeted interventions do not appear to produce different effects on knowledge, input use, market access, and economic benefits. However, the inspection of forest plots suggests that targeted interventions have a slightly larger impact on women's time use and empowerment indicators. Targeted interventions appear to improve women's non‐economic empowerment without creating larger economic benefits. Possibly, targeted interventions improve women's ability to make decisions, mobility, and participation through changes in gender attitudes, rather than through changes in economic power within the household. There are empowerment benefits of the interventions that are not directly brought about by an improvement in women's economic conditions.

Our third moderator analysis considered different types of value chain interventions operating under different mechanisms. Some interventions, such as contract farming, promote vertical integration and greater access to profitable markets. Other interventions, such as those promoting farmer‐based organisations, promote horizontal integration and more equitable access to markets. Finally, other interventions promote production or processing upgrading by creating access to products and services that are more profitable.

We considered the differential impacts of these types of interventions on all outcome categories, but we were not able to find any significant differences or meaningful patterns. The different intervention categories appear to produce very similar results on all outcome accounts. It is possible that a different classification of the interventions could produce different results, but there is an inherent difficulty in conducting this type of analysis, as the sample of available studies cannot be sliced into highly specific intervention types without losing precision and statistical power. Our current results suggest that the outcomes of value chain interventions are independent of the specific type of intervention implemented.

#### 6.1.5 Meta‐regression analysis

In the previous section we analysed the differences in study results by geographic area, targeting, and project type, one factor at a time. In this section we consider the differential impact of all factors at the same time using meta‐regression analysis (Table 8). We do not conduct a separate analysis for each outcome group, and we present the result of an omnibus analysis considering all outcomes at the same time. The factors included in the regression model fit the data reasonably well, and the model explains 36% of the observed data variance. Indicators of heterogeneity, however, point to a large variability in results.

**Table 8:** Meta regression.

Variables	exp (b)	SE	*t* Value	*p* Value
Bank account	0.892	0.114	−0.88	0.378
Bargaining	0.913	0.172	−0.48	0.632
Collective	0.994	0.132	−0.04	0.968
Credit	0.981	0.083	−0.22	0.829
Decisions	0.908	0.062	−1.41	0.161
Employment	1.011	0.093	0.13	0.898
Empowerment	1.03	0.092	0.33	0.742
Gender attitudes	**2.42**	**0.926**	**2.31**	**0.022**
Income and expenditure	1.007	0.061	0.12	0.906
Input costs	0.982	0.082	−0.21	0.838
Investment	1.137	0.204	0.71	0.477
Knowledge	0.796	0.107	−1.69	0.091
Labour inputs	0.947	0.145	−0.35	0.728
Land	1.04	0.084	0.52	0.606
Leadership	1.047	0.133	0.36	0.719
Leisure	0.857	0.106	−1.24	0.218
Mobility	0.792	0.097	−1.9	0.059
NA	1.005	0.071	0.08	0.936
Other improved inputs	**1.172**	**0.082**	**2.26**	**0.025**
Other market access	1.059	0.1	0.61	0.543
Participation	1.13	0.092	1.49	0.138
Prices	0.976	0.747	−0.31	0.755
Productivity	1.054	0.068	0.82	0.416
Quality standards	**0.44**	**0.101**	**−3.56**	**0**
Savings	1.085	0.137	0.65	0.516
Sustainable income	1.001	0.059	0.03	0.975
Training	1.082	0.121	0.7	0.484
Workload	0.917	0.0737	−1.07	0.287
East Asia and Pacific South Asia region	0.924	0.154	−0.47	0.64
Europe and Central Asia region	1.164	0.125	1.41	0.16
South Asia	**1.34**	**0.117**	**3.35**	**0.001**
South Asia and Sub‐Saharan Africa	1.214	0.2	1.18	0.24
Sub‐Saharan Africa	**1.18**	**0.081**	**2.42**	**0.017**
Latin America and the Caribbean	1.264	0.18	1.64	0.102
Targeted interventions	1.019	0.057	0.34	0.735
Enabling policies and institutional environment	1.118	0.067	1.84	0.067
Financial services	**0.728**	**0.039**	**−5.84**	**0**
Processing and storage facilities	**1.237**	**0.099**	**2.65**	**0.009**
Horizontal and vertical coordination	0.913	0.047	−1.75	0.082
Process, product and chain upgrading	0.938	0.043	−1.36	0.175
Promoting the production of new products	1.002	0.105	0.02	0.98
Improving market quality	0.959	0.068	−0.58	0.565
Supporting horizontal integration of producer groups	**1.355**	**0.076**	**5.4**	**0**
Improving processing techniques	**0.839**	**0.052**	**−2.79**	**0.006**
Contract farming	0.915	0.077	−1.05	0.294
Inclusive market systems development	**0.813**	**0.079**	**−2.1**	**0.037**
Access to markets	1.008	0.1	0.09	0.93
Market structures	**0.711**	**0.124**	**−1.94**	**0.053**
Number of observations	255			
Tau^2^	0.03128			
*I* ^2^ res	88.02%			
Adj *r* ^2^	35.91%			
*p* Value	0			

The results are in agreement with the analysis conducted in the previous section and do not provide additional relevant information. They confirm that interventions implemented in South Asia and Sub‐Saharan Africa are on average more successful than those in other areas of the world. They also confirm that interventions explicitly targeted to women do not seem to produce larger economic benefits than non‐targeted interventions. Targeted and non‐targeted interventions are likely to be very similar, and therefore tend to produce similar results. Many outcomes are reported at the household level, and it is difficult to say whether women benefit more from targeted interventions. It is also possible that targeted interventions occur in more challenging settings where improvements are more difficult to produce. Targeted interventions however appear to produce non‐economic empowerment impacts.

Finally, the results show that processing and storage facility interventions, and interventions supporting horizontal integration of producer groups, tend to achieve better results, while interventions promoting financial services, processing techniques, inclusive market system development, and market structure appear to perform worse than the average project. However, given the small number of studies in each category and the variety of mechanisms at play, it is difficult to discern any meaningful pattern in favour of any specific type of intervention from this analysis. We were able to identify a large number of studies because we adopted a broad definition of value chain interventions, but when we disaggregate the studies by intervention type, the number of studies becomes smaller and it is difficult to discern patterns from random variation.

### 6.2 Qualitative synthesis: Women in value chain processes

This report discusses qualitative synthesis of 55 process evaluations, 59 effectiveness studies and 4 mixed‐methods studies, the majority of which are from various regions of Africa, followed by India and Latin America.

A qualitative thematic synthesis of the studies was executed using a coding framework based on the conceptual elements that are the basis for a theory‐based approach. The qualitative data extracted from the studies were included using the ‘theory‐based systematic review matrix’. Key themes and common elements under each of these headings were identified and are summarised in this section. This section specifically reports on emergent themes with reference to the settings, recruitment/referral mechanisms, barriers and facilitators to participation, and the causal processes.

#### 6.2.1 Settings and recruitment/referral mechanisms

The interventions ranged from acquiring certifications, fair trade, co‐operatives and associations, self‐help groups, farmer research groups and contract farming. They took place primarily in community, institutional, and market settings where the participation and involvement of farmers (primarily women) was gauged through various indices. The majority of studies were set in rural areas, though some towns and urban areas also constituted part of the study.

In most cases, the study area was selected based on a representative sampling done in the area, for which data were provided by the co‐operatives, associations, NGOs and experts working on the ground with the intended population. In some studies, sampled organisations were selected from an available list of co‐operatives in the chosen geographical area (district, town, village) provided by a regional office (Dohmwirth & Hanisch, 2019; Kent, 2018).

While respondents were recruited based on their voluntary interest, various criteria and recruitment factors were taken into account to ensure a robust study, such as a single gender co‐operative or mixed‐gender co‐operative, number of members in a given group, minimum number of years constituting the members' experience, landowners, and so on (Dohmwirth & Hanisch, 2019; Holmes & Imai, 2019; Lukuyu et al., 2020; Papa et al., 2000). This enabled comparability among cases and in the identification of regions or groups that had higher and/or sustained involvement in certain agricultural activities or processes of the value chain (Othman et al., 2021).

Depending on the circumstances, key informants and stakeholders were identified through purposive sampling as well as snowball sampling. While in some cases, experts and officials on the ground helped to identify potential study participants (Altenbuchner et al., 2018; Asingwire, 2011; Oumer et al., 2014), in other situations, traditional social and religious leaders and elders in the community enabled recruitment (Porter et al., 2014).

Participatory rural appraisal survey results also enable participant recruitment into studies (Oumer et al., 2014). Focus group discussions in process evaluation reports are quite important for data analysis, and allowed authors to construct a sampling frame with good representation of actors from various backgrounds. For instance, in the case of a women's collective action group in Northern Kenya (Coppock, 2013), a focus group discussion made it possible to study 16 settlements in which all groups voluntarily participated in intensive interaction.

#### 6.2.2 Barriers and facilitators to participation

The data suggest that intervention participation was voluntary. In most cases, household and community settings facilitated and sustained the participation for respondents. The main barrier was identified as access to institutional provisions and resources.

The following indicators are argued to have facilitated participation:

1. Women's membership in groups such as associations (Cuellar‐Gomez, 2008; Islam, 2008; Keelson & Nanekum, 2021), co‐operatives (Bacon, 2010; Holmes & Imai, 2019; Papa et al., 2000; Pineda, 2019), self‐help groups (Arakal & Roy, 2015; Desai & Joshi, 2013; Kumar, 2021; Madheswaran & Dharmadhikary, 2001) facilitated their access to networks, market linkages, peer support, and in general, provided a more inclusive environment to motivate them.
2. Out‐migration of men empowered women and gave them greater decision‐making capacity. The absence of men enabled women's higher cognisance of and participation in market processes (Altenbuchner et al., 2021; Mitra, 2019; Pankaj, 2020).
3. Interestingly, as poverty increased, more women were enabled to move out of domestic spaces and seek work, since access to immediate income became important to support the family (Forsythe, 2016; Makita, 2009; Matenga, 2017).
4. Identification of gendered crops helped to acquire support for women's higher participation in certain processes related to the cultivation, marketing and wholesaling of those crops. For instance, the argument that cassava (Forsythe, 2016), coffee (Cuellar‐Gomez, 2008; Ya‐Bititi et al., 2015), groundnut (Okiror et al., 2021), or horticulture explicitly required women's dexterity (Dolan, 2001) in many ways helped to bring women farmers and entrepreneurs into the public sphere – although with many associated stereotypes and negative connotations.
5. Willingness of women to be associated and more involved with certain crops and in specific steps of the value chain processes in the name of tradition. In most societies in these papers, abiding to tradition, heritage and cultural identity was significant. Hence, many respondents would attribute their roles as ascribed to ‘that is how it was done by my mother’. Additionally, many women participants emphasised their desire to stay closer to their family, and work while taking care of their children. For instance, in Cuellar‐Gomez's (2008) study, some women explained that they decided to become coffee growers because it was easier for them to work at home, where they could also take care of their children and manage household chores.
6. Participation in intervention(s) offered better chances for women in combating their socioeconomic drudgery, accessing beneficiary services, and achieving a sense of agency. To this effect, workshops and gender sensitisation programmes aimed at gender equality facilitated participation in the interventions (Geleta et al., 2017; Lukuyu et al., 2020). In some cases, access to some kind of legal apparatus further augmented women's participation, such as in the case of co‐operatives offering legal remedies to their women members for familial disputes and land disputes to allow them to sustain themselves financially as well as socially (Ferguson & Kepe, 2011; Gallardo‐Fernández & Saunders, 2018; Geleta et al., 2017).
7. Success of other participants in the vicinity and community emerged as a trusted means of facilitating entry of women in any intervention and/or programme. It also enabled building of women‐only groups and women recruiting more women participants into the programme (Altenbuchner et al., 2018; Cuellar‐Gomez, 2008; Ferguson & Kepe, 2011; Oumer, 2014; Porter et al., 2014).

In qualitative synthesis, analysis of barriers and facilitators reveal that the same factor has the potential to be both a barrier as well as the facilitator. For instance, out‐migration of men was identified both as a barrier as well as a facilitator for women's emancipation in value chain processes. As the facilitator, the absence of men gave women a chance to take more decisions and have a larger role in market settings; however, it also increased the chore burdens of women, who reported juggling domestic chores and caring for the family. Similarly, although the gendered nature of cropping and cultivation practices empowered women to partake in specific steps of agricultural production, it also restricted their opportunities to go beyond the limited and stereotyped spatial scope of practices and engagement.

Some other identified barriers to women's participation in the value chain processes are discussed below:

1. Stereotypes conditioned by social norms and culture, which dictate that women ought to perform the primary role of caregiver first. As per the traditional norm, it was considered inappropriate for a woman to be present in the public sphere or hold membership of associations (Cuellar‐Gomez, 2008; Lyon, 2008).
2. Unavailability of women in the production process – at both the institutional and market level – due to paucity of time (time poverty) and household demands of labour limited women's chances of involvement. This was worsened by unavailability of labour that could otherwise be hired to ease the demands on women (Altenbuchner et al., 2018; Geleta et al., 2017).
3. High levels of illiteracy, and limited or no access to formal education and training, were recognised as major hindrances to women's participation at association level and in market spaces. The main drawback was reflected in the misplaced idea of what certifications, fair trade practices or organic cultivation had to offer farmers. Many participants who actively took part in the interventions were unaware of the idea behind the intervention and the benefits (monetary and otherwise) that they could draw from it. This further inhibited their access to formal organisations and capacity building resources (e.g., training and advisory services, demand for better market prices for their produce) (Altenbuchner et al., 2018; Asingwire, 2011; Mojo, 2017; Ortega et al., 2019; Perry, 2020; Utting‐Chamorro, 2005).
4. Hindered mobility was further exacerbated by the location of co‐operatives or markets. Greater distances made centres, co‐operatives, and markets inaccessible for women (Mohapatra, 2016; Mojo, 2017; Lyon et al., 2019).
5. Exclusion of women from the co‐operative level and public sphere, where most associations were led by men. Hierarchies and power play resulted in a lack of trust toward agents, co‐operative executives, and other middlemen, further limiting women's agency (Altenbuchner et al., 2018; Dohmwirth & Hanisch, 2019; Dolan, 2001).
6. Complex processes required to access ones' own land, in addition to inheritance rules and rights, limited women's access to land, social capital, and better prices in the market for their agricultural produce (Raynolds, 2002; Bacon, 2010; Quisumbing et al., 2013; Okiror et al., 2021).
7. State policies, political climates and religious ideologies also affected women's understanding of their roles in the family and in public life. Religious reforms in conjunction with state coercion in many regions influenced socioeconomic practices, along with the privatisation of resources – which, although they could benefit small groups of the population, usually resulted in loss of land, resources, indigenous practices and livelihoods for many (Badejo et al., 2017; Cuellar‐Gomez, 2008; Dolan, 2001; Mitra, 2019; Okiror et al., 2021; Ya‐Bititi et al., 2015).

#### 6.2.3 Barriers and facilitators to achieving outcomes

##### Facilitators to outcomes

The following factors were identified as facilitators in achieving the intended outcomes:

1. *Capacity building*. First and foremost, we identified that building women's capacities facilitates the achievement of intended outcomes. This includes increasing knowledge and skills and providing a platform in which to share this knowledge with family members and fellow farmers within and outside of their communities (Altenbuchner et al., 2021; Asingwire, 2011; Bacon, 2010; Oumer, 2014). It has also been identified that greater knowledge and skills increased women's abilities and desire to serve their communities by transferring skills to non‐members (Ferguson & Kepe, 2011).

Participating in the FRG is great for easy access to training and knowledge sharing about improved practices. I have benefited and my livelihood has changed tremendously … My decision‐making has also increased even in matters of resource use and other community issues. – Oumer (2014)

Working in a group has several advantages, particularly learning from each other and better access to experts, technologies and market. Through the FRG intervention, I was able to grow disease‐tolerant seed potatoes and earn more income. As a result, the number of my livestock increased. – Oumer (2014)

We are sharing in the group meeting and in village meeting and with other people, the neighbouring villages. They [conventional farmers] are also interested to join us … We exchange knowledge on organic agriculture with them. – Altenbuchner et al. (2021)

2. *Institutionalisation and network formation for women*. This includes women's co‐operatives, self‐help groups, and women's associations, and is the second most important facilitator to achieve the outcomes. It has helped women to increase their negotiating capacity, sense of ownership, and opportunities to network with other women from outside the community to share experiences, difficulties, and innovation. The organisation of women provides a conducive environment for them to actively participate (Cuellar‐Gomez, 2008; Gallardo‐Fernández & Saunders, 2018; Dohmwirth & Hanisch, 2019; Ferguson & Kepe, 2011).

My husband used to manage everything; he decided what he would buy and [he told me] that the wife should not get involved in these issues because she lacked the capacity. So for me this [group] is very important because we, as women, have learned that, yes, we have the capacity to manage ourselves and, yes, we can work – Bacon (2010, p. 61)

3. *Markets*. Better market access and infrastructure, support from the market, and better market linkages are also important to achieve economic empowerment (Arakal & Roy, 2015; Cuellar‐Gomez, 2008; Ferguson & Kepe, 2011).

Katrina Munda of Angara cluster left her job as an Anganwadi worker and is full time in lac production. She earns rupees three to five lakhs per year from lac and had started building a pukka house for herself. She plans to give up her below‐poverty line card! The highest earning lac producer is, however, Sugan Devi, who earns nearly rupees seven to eight lakhs per year. Cuellar‐Gomez (2008)

4. *Land ownership*. This includes women's control over assets; however, land ownership rights and women acquiring their own land helps them to gain control and decision‐making power (Bacon, 2010).

##### Barriers to outcomes

There are other factors that act as barriers to achieving these outcomes, such as the exploitation of women by ‘commissioning agents’ (Bacon, 2010; Oumer, 2014); lack of market information; decision‐making power (Altenbuchner et al., 2021) and land‐ownership rights (Altenbuchner, et al., 2021; Cuellar‐Gomez, 2008); male‐dominated leadership and management of fair trade businesses (Bacon, 2010); inability to acquire credit (Cuellar‐Gomez, 2008; Forsythe, 2016); lengthy and expensive processes for organic certification (Bacon, 2010); stereotypes attached to women's roles in farming, and limited opportunities and visibility (Cuellar‐Gomez, 2008), constraints on mobility, and cultural barriers (Arakal & Roy, 2015; Dohmwirth & Hanisch, 2019), and lack control over resources (Ferguson & Kepe, 2011).

There are also many external factors such as weather, natural disasters and climate change (Altenbuchner et al., 2021; Arakal & Roy, 2015; Cuellar‐Gomez, 2008), as well as resource constraints regarding electricity, ICT infrastructure, and communication networks (Asingwire, 2011).

Many studies have also identified women's increased workload as a barrier to achieving intended outcomes (Altenbuchner et al., 2021; Bacon, 2010; Cuellar‐Gomez, 2008; Dolan, 2001; Geleta et al., 2017).

#### 6.2.4 Causal processes

Three dominant causal pathways are identified in either facilitating or limiting intervention outcomes. These are related to lack of awareness of rights, illiteracy, out‐migration of male members, usage of religious ideology to rationalise gender roles, and the effect of formalisation/professionalisation of the trades in discussion.

Causal processes aimed at gender equity in these reports emphasise the role of capacity building for women's empowerment broadly. Diversified income sources and increased income for women resulted not only in better household living standards and financial agency but also the appreciation and support of husbands and men in general. Changing climatic conditions, loss of biodiversity, and loss of traditional practices due to intense commercialisation were also brought forth.

In addition, we highlight evidence of women's exclusion from the co‐operative level and the public sphere, where most associations are led by men – thereby negatively affecting women's freedom to participate or speak their minds:

- Lack of access to knowledge and illiteracy, in general, rendered access to services, resources and emancipatory avenues difficult for women. However, a lack of awareness in certain cases led to natural resource degradation – such as in the case of commercialisation of argan in Morocco (Perry, 2020) or organic cotton cultivation in India (wherein farmers resisted participating in the intervention due to the perceived threat of losing indigenous knowledge and practices, further resulting in loss of biodiversity) (Altenbuchner et al., 2018; Mitra, 2019).
- While the loss of traditional livelihoods and land to commercialisation forced men to migrate in search of better opportunities elsewhere, women took charge of their lands and families. Although this added to the existing demands on them, it also imparted a sense of ownership and agency to women who were now handling their domestic chores along with a source of income.
  Their decision‐making ability is higher, but the absence of risk‐taking capacity and other factors related to ownership of assets negatively affects the growth of entrepreneurship among women (Bacon, 2010; Namayengo, 2017; Pankaj, 2020; Utting‐Chamorro, 2005). In some situations, the absence of men exacerbates situations in which women need their assistance, such as in women‐only co‐operatives (Dohmwirth & Hanisch, 2019).
- Religious ideology in many cases is employed to rationalise the gendered roles both within and outside the domestic sphere. In addition to the local kinship and lineage patterns that shape inheritance and asset ownership in most communities, religious ideologies are often used to justify why women could not own any asset or land (Badejo et al., 2017; Dolan, 2001; Okiror et al., 2021).
- Professionalisation of trades also positively affected gender relations. Formal associations helped to sensitise the population, and contributed to gender equity measures through the gender programmes and workshops. This helped husbands to better understand and support their wives at household and farm levels. Fair trade practices and co‐operatives helped to establish a standardised verification process that helped women to gain access to external funding supporting gender programmes and other social programmes. However the informal nature of certain jobs and activities in value chain processes involved flexibility as well as risks, including uncertain chances in the of availability of credit and access to resources (Bacon, 2010; Cuellar‐Gomez, 2008).

### 6.3 Integrated synthesis: Women in value chain processes

Inequalities within a value chain can be based on gender, age, ethnicity, and other factors of social differentiation (Coles & Mitchell, 2011). One of the aims of this review is to identify the mechanisms through which value‐chains operate to create enabling as well as constrain participation, mobility and agency of women participants. Thus, it sought to answer how the value chain interventions contribute to women's economic empowerment.

In agriculture, women constitute 43% of the global agricultural workforce (Raney, 2011). They play a vital role in food production, processing, and distribution. As argued by Pyburn and Kruijssen (2020), research on gender dynamics in value chain development has grown more nuanced and sophisticated over time, but it still remains non‐linear and uneven. The evidence from the included studies in the review show that the outcomes are driven by the context‐mechanism framework. The context here refers to the socio‐economic, cultural, regional and traditional structures that shaped women's role in the operationalisation of the value chain.

#### 6.3.1 How do value chain interventions work?

As emphasised earlier, value chains are a result of the matrix of functions and channels that contribute toward the outcomes and overall impact for the stakeholders involved. In this review, we find that horizontal integration works in favour of the efficacy of any value chain. The qualitative synthesis found that interventions promoting horizontal integration through farmer‐based organisations, self‐help groups and co‐operatives tend to support in promotion of women's participation in, and access to economic benefits from, the interventions.

#### 6.3.2 Why do value chain interventions work or not work?

**Figure 21:**
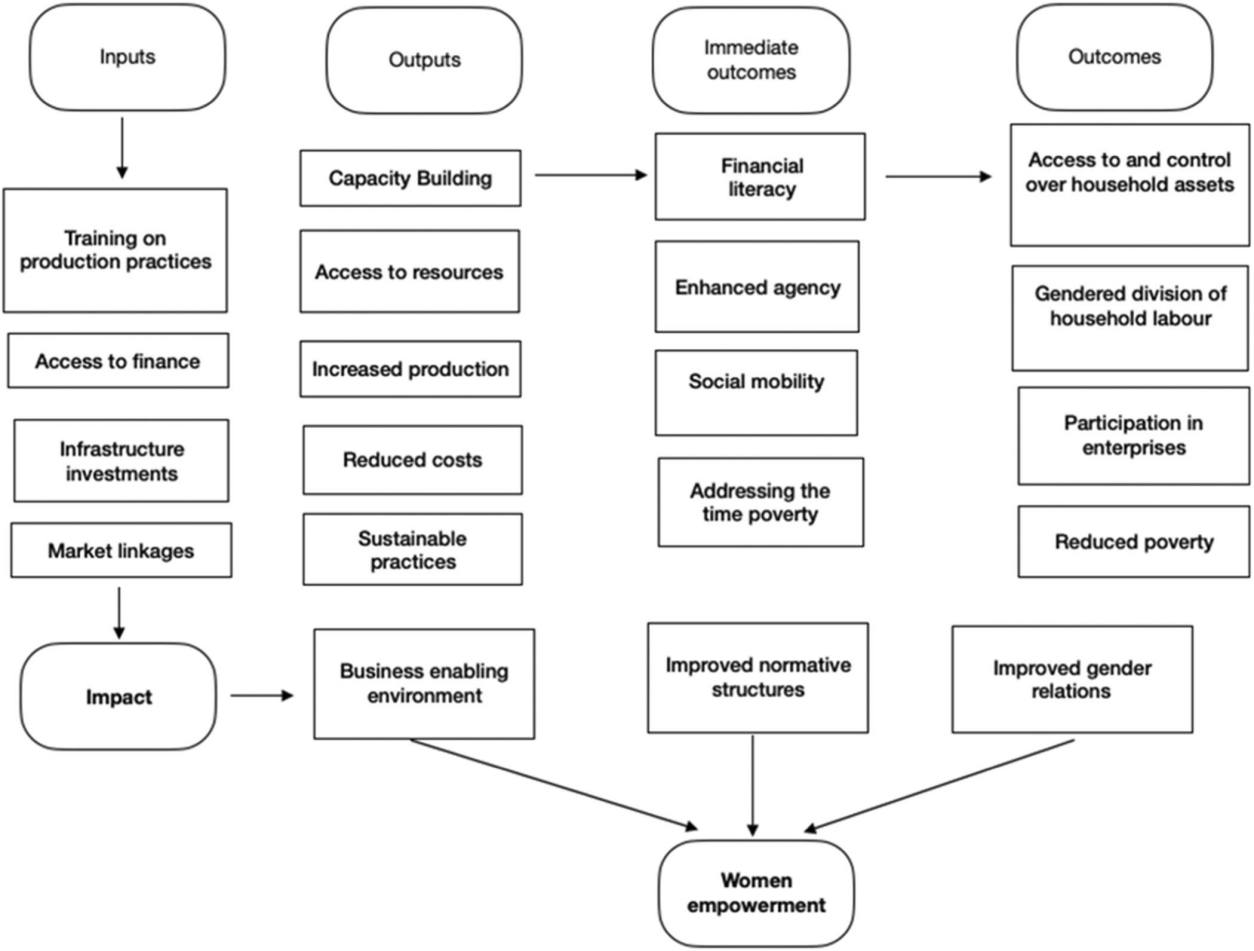
Revised theory of change.

Understanding the causal pathway is useful in responding to this question (Figure 21). These pathways show how women's engagement from production to distribution influence their economic empowerment. The concept of economic empowerment broadly encompasses access to resources, availability of finance, and agency of the participants. McKague and Siddiquee (2014) state that constraints in successful implementation as well as uptake of value chain interventions not only arise from business enabling environment but also physical infrastructure, human capabilities and social norms.

Three dominant causal pathways are identified in either facilitating or limiting the intervention outcomes. These are related to unawareness of ones' rights, illiteracy, out‐migration of male members and effect of formalisation/professionalisation of the trades in discussion (e.g., Elias, 2017). Causal processes aimed at gender equity in these reports emphasise upon the role of capacity building for women empowerment broadly. While diversified income sources and increased income for women resulted not only in better household living standards, financial agency, but it also garnered appreciation and support of women husbands and male folk in general. Changing climatic conditions, loss of biodiversity and loss of traditional practices caused due to intense commercialisation were also brought forth.

McKague and Siddiquee (2014, p. 47) argue that for value chain interventions to work, it is important that a ‘sufficient number of value chain actors find it in their self‐interest to implement and maintain them’, it has to be context driven. The authors in their work identify the unique challenges that emerge in a given place due to its specific market structure, prevalent social relations, business enabling environment, and customer demands.

The ToC in this review analyses the elements of value chain interventions at the micro and macro level to offer an overview the intermediate and final outcomes in shaping the economic empowerment of women participants in the value chain(s) as the desired impact. Macro level elements encompass the cultural, institutional, geographic, and economic characteristic, that is, the normative structures (norms, values, cultures) along with the prevalent economic and business ecosystem. The micro level elements include business capabilities, resources availability, financial literacy, cognitive capabilities, and so forth.

These are addressed by inputs such as training on improved production practices, access to finance, providing market linkages and capacity building measures including infrastructure investments. Activities such as providing training to farmers and other businesses, facilitate access to finance for farmers and other businesses, linking farmers and other businesses to high‐value markets and investment in infrastructure, facilitate in achieving the outputs of both increased productions well as improved efficiency of the value chain overall.

Intermediate outcomes such as income generation from the chain with enhanced mobility for women are of significance in sustained economic empowerment for women. But this is closely related to and shaped by women's access and ownership of assets. Her agency, finance, utilisation of and access to resources therefore are three main elements in understanding the gendered participation in value chain and collective enterprises. Economic empowerment for women can be best achieved when women have the power to make their own decisions and choices, based on their own knowledge and understanding, to improve their social status.

To this end, unpaid household labour, mostly done by women, limits women's time and energy to earn money through value chains (Stoian et al., 2018). The factor of time poverty is reported as one of the main reasons for hampering women's participation and empowerment in value chain(s). This further dampens their control over assets than men, reducing their decision‐making power and capacity to engage in more profitable nodes of value chains (Quisumbing et al., 2015).

Most of the evidence in this review is included from African regions. Those interventions implemented in Sub‐Saharan Africa and South Asia are consistently more successful for all outcomes considered.

## 7 AUTHOR'S CONCLUSIONS

### 7.1 Overview of findings

Our review finds that value chain interventions are successful in improving the economic conditions of their intended beneficiaries. We find that the interventions improve women's economic outcomes such as income, assets holdings, productivity, and savings. The meta‐analysis suggests that this occurs more through the acquisition of skills and improved inputs, rather than an improvement in access to profitable markets.

The quantitative analysis of the outcomes suggests that the interventions' success in improving women's economic outcomes does not always translate into an increase in non‐economic empowerment. We find a modest impact of the interventions on general empowerment indicators. The quantitative effects on women's mobility, decision‐making, and leadership of organisations are positive, but limited in size. This in turn suggests that although the interventions are able to change economic roles within value chains, these changes are not sufficiently strong to significantly alter gender roles within families and communities. Indeed, the qualitative evidence points to the persistence of cultural barriers and other constraints.

We find that interventions implemented in Sub‐Saharan Africa and South Asia are consistently more successful for all outcomes considered. However, it must be observed that few studies have been conducted in other areas of the world, meaning that estimates from this area lack precision and statistical power.

We find that value chain interventions specifically targeted to women do not produce significantly larger economic impacts than non‐targeted interventions. They seem, however, to produce slightly larger impacts on indicators of time use and non‐economic empowerment. Presumably, targeted interventions are not very different from targeted ones in terms of characteristics and implementation, and produce similar results. Targeted interventions, however, improve indicators of participation, leadership, mobility, and decision‐making, although this does not seem to further improve economic outcomes.

The qualitative findings identified facilitators to women's empowerment which included capacity building, network formation, market access and land ownership as well as barriers such as exploitation, lack of information, stereotypes and lack of control over resources.

The qualitative literature seems to suggest that interventions promoting horizontal integration through farmer‐based organisations, self‐help groups and co‐operatives tend to be important facilitators at promoting women's participation (including leadership and decision‐making, and access to economic benefits of the interventions).

This review also examines how interventions can improve women's participation and economic empowerment in agriculture. Value chain interventions improve women's incomes and skills, but cultural barriers limit broader social change and thus can disadvantage women due to social factors. Successful approaches include building women's capacity, addressing social barriers, and tailoring approaches to specific contexts. While targeted programmes see more participation, economic gains do not differ much. Unpaid domestic burdens remain a challenge. Regionally, Sub‐Saharan Africa and South Asia show the most success, but further research is needed elsewhere.

However, we were not able to find differences between interventions of different types. The impacts of the interventions appear to be independent of the specific project type and its operating mechanism.

### 7.2 Implications for research

Our review highlights a number of limitations in the existing evidence base. First, most available evidence is quasi‐experimental. Researchers have exploited secondary data in an efficient way and have analysed the data in imaginative ways. However, more experimental evidence is needed when implementation circumstances allow.

Second, the impacts of the interventions on male and female beneficiaries are not always separately reported and analysed. In addition, important empowerment outcomes such as effects of the interventions on time use are rarely reported. Researchers should pay more attention to gender‐disaggregated outcomes, and assess impacts over a wide range of empowerment outcomes.

Third, we cannot fail to observe how the available evidence is largely concentrated in Sub‐Saharan Africa and South Asia, and a handful of countries within these macro‐regions. This has implications on the generalisability of the results, as the available studies provide a limited range of contextual variations that are not representative of many other areas of the world in which value chain interventions are implemented. Researchers should devote more time to the study of geographically neglected regions.

### 7.3 Implications for policy and practice

The main conclusion of the review is that value chain interventions do empower women, but perhaps to a lesser extent than expected. Economic empowerment does not immediately translate into empowerment within families and communities. Interventions should either moderate their expectations on empowerment goals, or they should be implemented in a way that ensures higher rates of participation among women and the acquisition of greater decision‐making power.

Our review is unable to conclude whether some interventions are more successful than others or to point to intervention mechanisms that are more likely to improve empowerment indicators to a sufficient level of confidence. However, the qualitative and the quantitative evidence conducted so far seems to suggest that untargeted value chain interventions are unlikely to produce the same impact on women's empowerment as interventions that are specifically targeted to women. Targeted interventions not only promote women's access to profitable value chains; they also promote women's participation in the projects, their acquisition of leadership positions, and increased decision‐making power. As a result, they improve non‐economic empowerment more than non‐targeted interventions, although this does not seem to translate into larger economic benefits.

In addition, the qualitative evidence seems to suggest that interventions based on horizontal coordination, such as those promoting co‐operatives and other farmer‐based organisations, tend to support in increasing empowerment than interventions promoting vertical coordination (such as contract farming) or product upgrading (such as fair trade).

## ROLES AND RESPONSIBILITIES

- Content: Ranjitha Puskur and Edoardo Masset are responsible for content. Edoardo Masset is also the technical lead for the review.
- Systematic review methods: Edoardo Masset, Howard White, and Suchi Kapoor Malhotra are responsible for ensuring that satisfactory systematic review methods are used.
- Statistical analysis: Hugh Sharma Waddington leads on statistical analysis and Suchi Kapoor Malhotra and Neha Gupta assists.
- Qualitative data analysis: Suchi Kapoor Malhotra is responsible for performing qualitative data analysis and Swati Mantri assists.Information retrieval: Sarah Young, informational retrieval specialist, designed the searches based on the suggestion by Edoardo and Suchi. She did the information retrieval on the databases.

## POTENTIAL CONFLICT OF INTEREST STATEMENT

Howard White is CEO of the Campbell Collaboration. As CEO he has no role in the editorial process.

## SOURCES OF SUPPORT

This study is funded by the CGIAR Generating Evidence and New Directions for Equitable Results (GENDER) Platform.

## Supporting information

Supporting information.

## Footnotes

1 Product upgrading strategies comprise activities meant to improve a product's quality and increase its value for consumers or other intermediary use, whereas process upgrading strategies focus on enhancing production efficiency using improved production technology or better coordination of the production process. Further, functional upgrading strategies allow a value chain actor entry into a new and more enhanced value‐added function in the value chain.

2 See United Nations Children's Fund. (2017). *Gender equality: Glossary of terms and concepts*. UNICEF. https://www.unicef.org/rosa/sites/unicef.org.rosa/files/2018-06/Glossary%20of%20Gender%20Terms%20and%20Concepts%20UNICEF%20ROSA%20Nov%202017.pdf (Accessed 26 October 2020).

3 The framework was developed by KIT et al. (2006).

4 FAO (2016).

5 This notwithstanding, Ihalainen and colleagues (2021) stated instances where: (1) participation in value chains and value chain development can have disempowering effects; and (2) empowerment and disempowerment can run concurrently and produce contradicting effects within and beyond the value chain, as well as between different dimensions of empowerment.

6 As defined by the World Bank categorisation at the time the data were collected.

7 Available from https://www.campbellcollaboration.org/research-resources/effect-size-calculator.html.

8 Studies identified before the search to test the search. There were 16 of these, but 6 were duplicates of studies identified in the database search.

## References

[cl21428-bib-0001] Al‐Hassan, S. , Abdul‐Malik, A. , & Andani, A. (2011). The role of Grameen Ghana in improving income of women shea butter processors. Journal of Development and Agricultural Economics, 3(11), 537–544.

[cl21428-bib-0002] Ambler, K. , Jones, K. , & O'Sullivan, M. (2018). *What is the role of men in connecting women to cash crop markets? Evidence from Uganda* (Discussion Papers, No.1762). IFPRI.

[cl21428-bib-0003] Anand, P. , Saxena, S. , Gonzalez, R. , & H, D. H. A. (2019). *Can women's self‐help groups contribute to sustainable development? Evidence of capability changes from Northern India* (Policy Research Working Paper No.9011). World Bank.

[cl21428-bib-0004] Arslan, A. , Higgins, D. , Winters, P. , & Bresciani, F. (2018). Impact assessment: Irrigated rice production enhancement project (IRPEP). IFAD.

[cl21428-bib-0005] Arslan, A. , Higgins, D. , Egger, E.‐M. , & Zucchini, E. (2020). Impact assessment: Strengthening local development in the highlands and high rainforest areas project (PSSA). IFAD.

[cl21428-bib-0006] Bonilla, J. , McCarthy, N. , Mugatha, S. , Rai, N. , Coombes, A. , & Brubaker, J. (2017). *Impact Assessment: Smallholder Dairy Commercialization Programme (SDCP)*. 3ie Grantee Final Report. International Initiative for Impact Evaluation (3ie).

[cl21428-bib-0007] Cavatassi, R. , & Mallia, P. (2018). Impact assessment: Livestock and pasture development project. IFAD.

[cl21428-bib-0008] Cavatassi, R. , Mabiso, A. , & Brueckmann, P. (2019). Impact assessment: The Coastal Community Development (CCDP). IFAD.

[cl21428-bib-0009] Cole, S. M. , Kaminski, A. M. , McDougall, C. , Kefi, A. S. , Marinda, P. A. , Maliko, M. , & Mtonga, J. (2020). Gender accommodative versus transformative approaches: A comparative assessment within a post‐harvest fish loss reduction intervention. Gender, Technology and Development, 24(1), 48–65.

[cl21428-bib-0010] Crookston, B. T. , West, J. H. , Davis, S. F. , Hall, P. C. , Seymour, G. , & Gray, B. L. (2021). Understanding female and male empowerment in Burkina Faso using the project‐level Women's Empowerment in Agriculture Index (pro‐WEAI): A longitudinal study. BMC Women's Health, 21(230), 230. 10.1186/s12905-021-01371-9 34082722 PMC8173955

[cl21428-bib-0011] Desai, R. M. , & Joshi, S. (2013). *Collective action and community development: Evidence from self‐help groups in rural India* (Policy Research Working Paper No.6547). World Bank.

[cl21428-bib-0012] Desai, R. M. , & Joshi, S. (2014). Can producer associations improve rural livelihoods? Evidence from farmer centres in India. The Journal of Development Studies, 50(1), 64–80.

[cl21428-bib-0013] Dohmwirth, C. , & Liu, Z. (2020). Does cooperative membership matter for women's empowerment? Evidence from South Indian dairy producers. Journal of Development Effectiveness, 12(2), 133–150.

[cl21428-bib-0014] Ebata, A. , & Huettel, S. (2017). The effect of value chain interventions for staple crops: Evidence from small‐scale farmers in Nicaragua. The Journal of Development Studies, 55(4), 581–596. 10.1080/00220388.2017.1408794

[cl21428-bib-0402] Elias, M. , & Arora‐Jonsson, S. (2017). Negotiating across difference: Gendered exclusions and cooperation in the shea value chain. Environment and Planning D: Society and Space, 35(1), 107–125.

[cl21428-bib-0015] Fischer, E. , & Qaim, M. (2012). Gender, agricultural commercialization, and collective action in Kenya. Food Security, 4(3), 441–453. 10.1007/s12571-012-0199-7

[cl21428-bib-0016] Fuller, R. (2013). *Food security and livelihoods support among fishers and fish processors in Kasenyi and Tchomia project effectiveness review – Full technical report*. Oxfam GB.

[cl21428-bib-0017] Garbero, A. , & Cichaibelu, B. B. (2018). Impact assessment: Agricultural Sector Development Programme–Livestock (ASDP‐L) and Agriculture Service Support Programme (ASSP). IFAD.

[cl21428-bib-0018] Garbero, A. , Diatta, D. , & Olapade, M. (2018). Impact assessment: Agricultural Value Chains Support Project. IFAD.

[cl21428-bib-0019] Garbero, A. , & Songsermsawas, T. (2018). Impact assessment: Guangxi Integrated Agricultural Development Project. IFAD.

[cl21428-bib-0020] Ghebru, H. , Smart, J. , & Mogues, T. (2019). *Access to markets for smallholder farmers in Alto Molócue and Molumbo, Mozambique: Mid‐term impact evaluation of InovAgro II* (Discussion papers, No.1877). IFPRI.

[cl21428-bib-0021] Gichungi, H. , Muriithi, B. , Irungu, P. , Diiro, G. , & Busienei, J. (2020). Effect of technological innovation on gender roles: The case of fruit fly IPM adoption on women's decision‐making in mango production and marketing in Kenya. The European Journal of Development Research, 33(3), 407–426. 10.1057/s41287-020-00282-z

[cl21428-bib-0022] Handschuch, C. , & Wollni, M. (2016). Traditional food crop marketing in sub‐Saharan Africa: Does gender matter? The Journal of Development Studies, 52(3), 343–359. 10.1080/00220388.2015.1068289

[cl21428-bib-0023] Hughes, K. , Morgan, S. , Baylis, K. , Oduol, J. , Smith‐Dumont, E. , Vågen, T. G. , & Kegode, H. (2018). Assessing the downstream socioeconomic impacts of agroforestry in Kenya (Working Paper No.291). World Agroforestry Centre.

[cl21428-bib-0024] Ishaq, M. N. , Xia, L. C. , Rasheed, R. , Ahmad, Z. , & Abdullah, M. (2016). Alternative milk marketing channels and dairy performance of smallholders in Pakistan: A case of south region of Punjab province. Sarhad Journal of Agriculture, 32(4), 304–315. 10.17582/journal.sja/2016.32.4.304.315

[cl21428-bib-0025] Kafle, K. , Krah, K. , & Songsermsawas, T. (2018). Impact assessment: High‐value agriculture project in hill and mountain areas. IFAD.

[cl21428-bib-0026] Kafle, K. , Songsermsawas, T. , & Winters, P. (2021). Impacts of agricultural value chain development in a mountainous region: Evidence from Nepal. IFAD.

[cl21428-bib-0027] Kamau, C. N. , Kabuage, L. W. , & Bett, E. K. (2018). Impact of improved indigenous chicken breeds on productivity. The case of smallholder farmers in Makueni and Kakamega counties, Kenya. Cogent Food & Agriculture, 4, 1477231. 10.1080/23311932.2018.1477231

[cl21428-bib-0028] Kinati, W. , Bekele, A. , & Chinnan, K. P. M. (2014). Impact of farmer research group interventions on maize farmers in Central Rift Valley of Oromia: An empirical study. Journal of Agricultural Extension and Rural Development, 6(3), 94–107.

[cl21428-bib-0029] Knößlsdorfer, I. , Sellare, J. , & Qaim, M. (2021). Effects of fairtrade on farm household food security and living standards: Insights from Côte d'Ivoire. Global Food Security, 29, 100535. 10.1016/j.gfs.2021.100535

[cl21428-bib-0030] Kolade, O. , & Harpham, T. (2014). Impact of cooperative membership on farmers' uptake of technological innovations in Southwest Nigeria. Development Studies Research, 1(1), 340–353. 10.1080/21665095.2014.978981

[cl21428-bib-0031] Kumar, N. , Raghunathan, K. , Arrieta, A. , Jilani, A. , & Pandey, S. (2021). The power of the collective empowers women: Evidence from self‐help groups in India. World Development, 146, 105579. 34602708 10.1016/j.worlddev.2021.105579 PMC8350313

[cl21428-bib-0032] Lecoutere, E. (2017). The impact of agricultural co‐operatives on women's empowerment: Evidence from Uganda. Journal of Co‐operative Organization and Management, 5(1), 14–27. 10.1016/j.jcom.2017.03.001

[cl21428-bib-0033] Leight, J. , Awonon, J. , Pedehombga, A. , Ganaba, R. , Martinez, E. , Heckert, J. , & Gelli, A. (2021). The impact of an integrated value chain intervention on household poultry production in Burkina Faso: Evidence from a randomized controlled trial. Journal of Development Effectiveness, 14(2), 108–124. 10.1080/19439342.2021.1968932

[cl21428-bib-0034] Liu, Z. , Rommel, J. , & Feng, S. (2018). Does it pay to participate in decision‐making? Survey evidence on land co‐management in Jiangsu Province, China. Ecological Economics, 143, 199–209.

[cl21428-bib-0035] Mabiso, A. , Abouaziza, M. , Wood, B. D. K. , & Balint, T. (2018). Impact assessment: Project for Rural Income through Exports in Rwanda. IFAD.

[cl21428-bib-0036] Manza, E. A. G. , & Banta, A. L. (2014). Impact of promoting sustainable agriculture project on livelihood sources in southern Borno state, Nigeria (PROSAB): A quantitative and qualitative analysis. Nigerian Agricultural Journal, 45(1), 1–12.

[cl21428-bib-0037] Meemken, E. M. , & Qaim, M. (2018). Can private food standards promote gender equality in the small farm sector? Journal of Rural Studies, 58, 39–51. 10.1016/j.jrurstud.2017.12.030

[cl21428-bib-0038] Meemken, E.‐M. , Sellare, J. , Kouame, C. N. , & Qaim, M. (2019). Effects of fairtrade on the livelihoods of poor rural workers. Nature Sustainability, 2(7), 635–642. 10.1038/s41893-019-0311-5

[cl21428-bib-0039] Minah, M. (2022). What is the influence of government programs on farmer organizations and their impacts? Evidence from Zambia. Annals of Public and Cooperative Economics, 93(1), 29–53.

[cl21428-bib-0040] Miura, K. , Kijima, Y. , & Sakurai, T. (2020). *Intrahousehold bargaining and agricultural technology adoption: Experimental evidence from Zambia* (GRIPS Discussion Paper 20/01). National Graduate Institute for Policy Studies.

[cl21428-bib-0041] Mohapatra, S. , & Sahoo, B. K. (2016). Determinants of participation in self‐help‐groups (SHG) and its impact on women empowerment. *Indian Growth and Development Review*.

[cl21428-bib-0042] Mwambi, M. , Bijman, J. , & Galie, A. (2021). The effect of membership in producer organizations on women's empowerment: Evidence from Kenya. Women's Studies International Forum, 87, 102492. 10.1016/j.wsif.2021.102492

[cl21428-bib-0043] Navarra, C. (2018). *Contract farming in Mozambique: Implications on gender inequalities within and across rural households* (Working Papers, No.26). WIDER.

[cl21428-bib-0044] Ocasio, V. M. (2016). Financing village enterprises in rural Bangladesh: What determines non‐farm revenue growth? International Journal of Development Issues, 15(1), 76–94. 10.1108/IJDI-09-2015-0057

[cl21428-bib-0045] Ortega, D. L. , Bro, A. S. , Clay, D. C. , Lopez, M. C. , Tuyisenge, E. , Church, R. A. , & Bizoza, A. R. (2019). Cooperative membership and coffee productivity in Rwanda's specialty coffee sector. Food Security, 11(4), 967–979. 10.1007/S12571-019-00952-9

[cl21428-bib-0046] Panda, N. , Mahapatra, A. S. , & Samal, R. (2012). Impact evaluation of SGSY on socio‐economic development of women in aquaculture in Eastern Hills of Orissa. Aquaculture International, 20(2), 233–247. 10.1007/s10499-011-9452-x

[cl21428-bib-0047] Pandey, V. , Nagarajan, H. K. , & Kumar, D. (2021). Impact of gendered participation in market‐linked value‐chains on economic outcomes: Evidence from India. Food Policy, 104, 102142. 10.1016/j.foodpol.2021.102142

[cl21428-bib-0048] Pandey, V. , Singh, S. , & Unni, J. (2020). Markets and spillover effects of political institutions in promoting women's empowerment: Evidence from India. Feminist Economics, 26(4), 1–30.

[cl21428-bib-0049] Pellegrina, L. D. , Di Maio, G. , Landoni, P. , & Rusinà, E. (2021). Money management and entrepreneurial training in microfinance: Impact on beneficiaries and institutions. Economia Politica, 38(3), 1049–1085.

[cl21428-bib-0050] Qiao, Y. , Halberg, N. , Vaheesan, S. , & Scott, S. (2016). Assessing the social and economic benefits of organic and fair trade tea production for small‐scale farmers in Asia: A comparative case study of China and Sri Lanka. Renewable Agriculture and Food Systems, 31(3), 246–257.

[cl21428-bib-0051] Quisumbing, A. R. , Rubin, D. , Manfre, C. , Waithanji, E. , van den Bold, M. , Olney, D. , Johnson, N. , & Meinzen‐Dick, R. (2015). Gender, assets, and market‐oriented agriculture: Learning from high‐value crop and livestock projects in Africa and Asia. Agriculture and Human Values, 32(4), 705–725. 10.1007/s10460-015-9587-x

[cl21428-bib-0052] Raghunathan, K. , Kannan, S. , & Quisumbing, A. R. (2019). Can women's self‐help groups improve access to information, decision‐making, and agricultural practices? The Indian case. Agricultural Economics, 50(5), 567–580. 10.1111/agec.12510 31762523 PMC6853198

[cl21428-bib-0053] Shah, G.‐M. , Khadka, M. S. , Ahmad, F. , Budhathoki, N. , & Shrestha, A. J. (2017). Assessment of Himalayan nettle (*Girardinia diversifolia*) value chain development interventions: Evidences from rural households in the far western Nepal. Journal of Agricultural Science, 9(5), 19. 10.5539/jas.v9n5p19

[cl21428-bib-0054] Van Campenhout, B. (2013). Is there an app for that? The impact of community knowledge workers in Uganda (Social Science Research Network, IFPRI Discussion Paper 01316). 10.2139/SSRN.2405695

[cl21428-bib-0055] Van Rijsbergen, B. , Elbers, W. , Ruben, R. , & Njuguna, S. N. (2016). The ambivalent impact of coffee certification on farmers' welfare: A matched panel approach for cooperatives in Central Kenya. World development, 77, 277–292.

[cl21428-bib-0056] Väth, S. J. , Gobien, S. , & Kirk, M. (2019). Socio‐economic well‐being, contract farming and property rights: Evidence from Ghana. Land Use Policy, 81(81), 878–888. 10.1016/J.LANDUSEPOL.2017.04.023

[cl21428-bib-0057] Väth, S. , & Kirk, M. (2014). *Do property rights and contract farming matter for rural development? Evidence from a large‐scale investment in Ghana* (MAGKS Joint Discussion Paper Series in Economics, No. 16‐2014). Philipps‐University Marburg, Faculty of Business Administration and Economics. https://explore.openalex.org/works/W2247100515

[cl21428-bib-0058] Warinda, E. , Nyariki, D. M. , Wambua, S. , Muasya, R. M. , & Hanjra, M. A. (2020). Sustainable development in East Africa: Impact evaluation of regional agricultural development projects in Burundi, Kenya, Rwanda, Tanzania, and Uganda. Natural Resources Forum, 44(1), 3–39. 10.1111/1477-8947.12191

[cl21428-bib-0059] Winters, P. , Simmons, P. , & Patrick, I. (2005). Evaluation of a hybrid seed contract between smallholders and a multinational company in East Java, Indonesia. Journal of Development Studies, 41(1), 62–89. http://www.tandfonline.com/loi/fjds20

[cl21428-bib-0401] Zulu, L. C. , Djenontin, I. N. S. , Darkwah, A. , Kamoto, J. , Kampanje‐Phiri, J. , Fischer, G. , Grabowski, P. , & Egyir, I. (2020). Realizing inclusive SAI: Contextualizing indicators to better evaluate gender and intergenerational inequity in SAI processes and outcomes ‐ cases from Southern and Western Africa. International Journal of Agricultural Sustainability, 19(5–6), 376–402. 10.1080/14735903.2020.1737356

[cl21428-bib-0060] Altenbuchner, C. , Vogel, S. , & Larcher, M. (2018). Social, economic and environmental impacts of organic cotton production on the livelihood of smallholder farmers in Odisha, India. Renewable Agriculture and Food Systems, 33(4), 373–385. 10.1017/s174217051700014x

[cl21428-bib-0061] Altenbuchner, C. , Vogel, S. , & Larcher, M. (2021). Community transformation through certified organic cotton initiatives – An analysis of case studies in Peru, Tanzania and India. Renewable Agriculture and Food Systems, 36(1), 38–53. 10.1017/S1742170519000462

[cl21428-bib-0062] Arakal, J. J. , & Roy, R. (2015). Udyogini and lac producers in Jharkhand: Catalyzing inclusive value chains at the base of the pyramid. South Asian Journal of Business & Management Cases, 4(1), 100–110. 10.1177/2277977915574043

[cl21428-bib-0063] Asingwire, N. , & Okello, J. J. (2011). Challenges facing smallholder farmers' ICT‐based Market Information Service (MIS) Projects: The case of Brosdi and Wougnet in Uganda.

[cl21428-bib-0064] Bacon, C. M. (2010). A spot of coffee in crisis: Nicaraguan smallholder cooperatives, fair trade networks, and gendered empowerment. Latin American Perspectives, 37(2), 50–71. 10.1177/0094582X09356958

[cl21428-bib-0065] Badejo, A. F. , Majekodunmi, A. O. , Kingsley, P. , Smith, J. , & Welburn, S. C. (2017). The impact of self‐help groups on pastoral women's empowerment and agency: A study in Nigeria. Pastoralism: Research, Policy and Practice, 7, 28. 10.1186/s13570-017-0101-5 32010440 PMC6961475

[cl21428-bib-0066] Coppock, D. L. , & Desta, S. (2013). Collective action, innovation, and wealth generation among settled pastoral women in northern Kenya. Rangeland Ecology & Management, 66(1), 95–105.

[cl21428-bib-0067] Cuellar‐Gomez, O. L. (2008). *Coffee produced by women in Cauca, Colombia: Where has Juanita Valdez been*? [Masters degree dissertation, Center for Latin American Studies]. Masters Abstracts International. https://www.proquest.com/dissertations-theses/coffee-produced-women-cauca-colombia-where-has/docview/60361862/se-2?accountid=9902

[cl21428-bib-0068] Dohmwirth, C. , & Hanisch, M. (2019). Women's active participation and gender homogeneity: Evidence from the South Indian dairy cooperative sector. Journal of Rural Studies, 72, 125–135.

[cl21428-bib-0069] Dolan, C. (2001). The “good wife”: Struggles over resources in the Kenyan horticultural sector. Journal of Development Studies, 37(3), 39–70. 10.1080/00220380412331321961

[cl21428-bib-0071] Eissler, S. , Sanou, A. , Heckert, J. , Myers, E. C. , Nignan, S. , Thio, E. , Pitropia, L. A. , Ganaba, R. , Pedehombga, A. , & Gelli, A. (2020). *Gender dynamics, women's empowerment, and diets: Qualitative findings from an impact evaluation of a nutrition‐sensitive poultry value chain intervention in Burkina Faso* (Discussion Paper 01913). IFPRI.

[cl21428-bib-0072] Ferguson, H. , & Kepe, T. (2011). Agricultural cooperatives and social empowerment of women: A Ugandan case study. Development in Practice, 21(3), 421–429. 10.1080/09614524.2011.558069

[cl21428-bib-0073] Forsythe, L. , Posthumus, H. , & Martin, A. (2016). A crop of one's own? Women's experiences of cassava commercialization in Nigeria and Malawi. Journal of Gender, Agriculture and Food Security, 1(2), 110–128.

[cl21428-bib-0074] Galiè, A. , Jiggins, J. , Struik, P. C. , Grando, S. , & Ceccarelli, S. (2017). Women's empowerment through seed improvement and seed governance: Evidence from participatory barley breeding in pre‐war Syria. NJAS: Wageningen Journal of Life Sciences, 81, 1–8. 10.1016/j.njas.2017.01.002

[cl21428-bib-0075] Gallardo‐Fernández, G. L. , & Saunders, F. (2018). “Before we asked for permission, now we only give notice”: Women's entrance into artisanal fisheries in Chile. Maritime Studies, 17(2), 177–188. 10.1007/s40152-018-0110-z

[cl21428-bib-0076] Geleta, E. B. , Elabor‐Idemudia, P. , Henry, C. , & Reggassa, N. (2017). The challenges of empowering women: The experience of pulse innovation project in Southern Ethiopia. SAGE Open, 7(4), 215824401773680.

[cl21428-bib-0077] Gurung, M. B. , Partap, U. , & Choudhary, D. (2015). Empowering mountain women through community‐based high value product value chain promotion in Nepal. International Journal of Agricultural Resources, Governance and Ecology, 11(3–4), 330–345. 10.1504/IJARGE.2015.074101

[cl21428-bib-0078] Hebo, M. (2014). Evolving markets, rural livelihoods, and gender relations: The view from a milk‐selling cooperative in the Kofale District of West Arsii, Ethiopia. African Study Monographs, 48, 5–29.

[cl21428-bib-0079] Hutchens, A. (2010). Empowering women through fair trade? Lessons from Asia. Third World Quarterly, 31(3), 449–467. 10.1080/01436597.2010.488477

[cl21428-bib-0080] Iradukunda, F. , Bullock, R. , Rietveld, A. , & van Schagen, B. (2019). Understanding gender roles and practices in the household and on the farm: Implications for banana disease management innovation processes in Burundi. Outlook on Agriculture, 48(1), 37–47. 10.1177/0030727019831704

[cl21428-bib-0081] Islam, M. S. (2008). *Environmental governance in the global agro‐food system: A study of shrimp aquaculture in Bangladesh* [Dissertation, York University]. Dissertation Abstracts International. https://www.proquest.com/dissertations-theses/environmental-governance-global-agro-food-system/docview/61746438/se-2?accountid=9902

[cl21428-bib-0082] Johnson, N. , Njuki, J. , Waithanji, E. , Nhambeto, M. , Rogers, M. , & Kruger, E. H. (2015). The gendered impacts of agricultural asset transfer projects: Lessons from the Manica Smallholder Dairy Development Program. Gender, Technology and Development, 19(2), 145–180.

[cl21428-bib-0083] Jones, E. , Smith, S. , & Wills, C. (2012). Women producers and the benefits of collective forms of enterprise. Gender & Development, 20(1), 13–32. 10.1080/13552074.2012.663640

[cl21428-bib-0084] Kasente, D. (2012). Fair trade and organic certification in value chains: Lessons from a gender analysis from coffee exporting in Uganda. Gender & Development, 20(1), 111–127.

[cl21428-bib-0085] Keelson, S. A. , & Nanekum, I. (2021). The role of informal commodity traders' association in the marketing of farm products in selected rural markets in Ghana. Journal of Development and Agricultural Economics, 13(4), 314–324. 10.5897/JDAE2021.1277

[cl21428-bib-0086] Kent, R. (2018). “Helping” or “appropriating”? Gender relations in shea nut production in northern Ghana. Society & Natural Resources, 31(3), 367–381. 10.1080/08941920.2017.1382626

[cl21428-bib-0087] Khatun, K. , Maguire‐Rajpaul, V. A. , Asante, E. A. , & McDermott, C. L. (2020). From agroforestry to agroindustry: Smallholder access to benefits from oil palm in Ghana and the implications for sustainability certification. Frontiers in Sustainable Food Systems, 4, 29. 10.3389/fsufs.2020.00029

[cl21428-bib-0088] Kwaramba, H. M. , Chigumira, E. , & Zimori, L. (2020). Women empowerment, agriculture commercialisation and gender relations: A value chain analysis, Mvurwi, Zimbabwe (Working Paper 42). Future Agricultures Consortium.

[cl21428-bib-0089] LeBaron, G. , & Gore, E. (2020). Gender and forced labour: Understanding the links in global cocoa supply chains. The Journal of Development Studies, 56(6), 1095–1117.

[cl21428-bib-0090] Loconto, A. M. (2011). *SustainabiliTea: Shaping sustainability in Tanzanian tea production* [Doctoral dissertation, Michigan State University]. Dissertation Abstracts International.

[cl21428-bib-0091] Lukuyu, B. , Mutambo, I. , & Ouma, E. (2020). Gender dynamics and social implications of improved planted forages in smallholder piggery systems in Uganda. CGIAR.

[cl21428-bib-0092] Lyon, S. (2008). We want to be equal to them: Fair‐trade coffee certification and gender equity within organizations. Human Organization, 67(3), 258–268.

[cl21428-bib-0093] Lyon, S. , Mutersbaugh, T. , & Worthen, H. (2019). Constructing the female coffee farmer: Do corporate smart‐economic initiatives promote gender equity within agricultural value chains? Economic Anthropology. 6(1), 34–47.

[cl21428-bib-0094] Makita, R. (2009). The visibility of women's work for poverty reduction: Implications from non‐crop agricultural income‐generating programs in Bangladesh. Agriculture and Human Values, 26(4), 379–390. 10.1007/s10460-008-9167-4

[cl21428-bib-0095] Manchón, B. G. , & Macleod, M. (2010). Challenging gender inequality in farmers' organisations in Nicaragua. Gender & Development, 18(3), 373–386. 10.1080/13552074.2010.521984

[cl21428-bib-0096] Le Mare, A. (2012). ‘Show the world to women and they can do it’: Southern fair trade enterprises as agents of empowerment. Gender & Development, 20(1), 95–109.

[cl21428-bib-0097] Matenga, C. R. (2017). Outgrowers and livelihoods: The case of Magobbo smallholder block farming in Mazabuka district in Zambia. Journal of Southern African Studies, 43(3), 551–566. 10.1080/03057070.2016.1211402

[cl21428-bib-0098] Mitra, A. , & Rao, N. (2019). Contract farming, ecological change and the transformations of reciprocal gendered social relations in Eastern India. The Journal of Peasant Studies, 48(2), 436–457. 10.1080/03066150.2019.1683000

[cl21428-bib-0099] Okiror, J. J. , Twanza, B. , Orum, B. , Ebanyat, P. , Kule, E. B. , Tegbaru, A. , & Ayesiga, C. (2021). For whom will the crop be promoted? A search for gender equity along the grain‐legume value chains in Uganda. Journal of Agricultural Extension and Rural Development, 13(4), 252–264. 10.5897/JAERD2017.0872

[cl21428-bib-0100] Ortiz‐Miranda, D. , & Moragues‐Faus, A. M. (2015). Governing fair trade coffee supply: Dynamics and challenges in small farmers' organizations. Sustainable Development, 23(1), 41–54. 10.1002/SD.1570

[cl21428-bib-0101] Othman, M. S. , Garrod, G. , & Oughton, E. (2021). Farming groups and empowerment of women smallholder farmers. Development in Practice, 31(5), 676–689. 10.1080/09614524.2021.1911947

[cl21428-bib-0102] Oumer, A. M. , Tiruneh, W. G. , & Tizale, C. Y. (2014). Empowering smallholder women farmers through participatory seed potato management: Lessons from Welmera district, Ethiopia. Journal of Sustainable Development, 7(5), 93–110. 10.5539/jsd.v7n5p93

[cl21428-bib-0103] Pankaj, A. (2020). Jeevika, women and rural Bihar: Cultural impact of a development intervention. Sociological Bulletin, 69(2), 158–173. 10.1177/0038022920923205

[cl21428-bib-0104] Papa, M. J. , Singhal, A. , Ghanekar, D. V. , & Papa, W. H. (2000). Organizing for social change through cooperative action: The [dis]empowering dimensions of women's communication. Communication Theory, 10(1), 90–123. https://www.proquest.com/scholarly-journals/organizing-social-change-through-cooperative/docview/214708114/se-2?accountid=9902

[cl21428-bib-0105] Perry, W. (2020). Social sustainability and the argan boom as green development in Morocco. World Development Perspectives, 20, 100238. 10.1016/j.wdp.2020.100238

[cl21428-bib-0106] Pineda, J. A. , Piniero, M. , & Ramírez, A. (2019). Coffee production and women's empowerment in Colombia. Human Organization, 78(1), 64–74.

[cl21428-bib-0107] Porter, R. , & Zovighian, D. (2014). Unpacking performance and empowerment in female farmers' groups: The case of the Fadama project in Nigeria. World Bank Group.

[cl21428-bib-0108] Ragasa, C. , Malapit, H. J. , Rubin, D. , Myers, E. , Pereira, A. , Martinez, E. M. , Heckert, J. , Seymour, G. , Mzungu, D. , Kalagho, K. , Kazembe, C. , Thunde, J. , & Mswelo, G. (2021). *‘It takes two’: Women's empowerment in agricultural value chains in Malawi* (Discussion Papers, 2006). IFPRI.

[cl21428-bib-0109] Raynolds, L. T. (2002). Wages for wives: Renegotiating gender and production relations in contract farming in the Dominican Republic. World Development, 30(5), 783–798. 10.1016/S0305-750X(02)00008-6

[cl21428-bib-0110] Rice, M. J. , Apgar, J. M. , Schwarz, A. M. , Saeni, E. , & Teioli, H. (2019). Can agricultural research and extension be used to challenge the processes of exclusion and marginalisation? The Journal of Agricultural Education and Extension, 25(1), 79–94. 10.1080/1389224X.2018.1529606

[cl21428-bib-0111] Utting‐Chamorro, K. (2005). Does fair trade make a difference? The case of small coffee producers in Nicaragua. Development in Practice, 15(3/4), 584–599. 10.1080/09614520500075706

[cl21428-bib-0112] Venema, B. , & van Eijk, J. (2004). Livelihood strategies compared: Private initiatives and collective efforts of Wolof women in Senegal. African Studies, 63(1), 51–71. 10.1080/0002018042000226157

[cl21428-bib-0113] Ya‐Bititi, G. M. , Lebailly, P. , & Mbonyinkebe, D. (2015). *“Coffee has given us voice”: Coffee cooperatives and women empowerment in Rwanda's rural areas*. Paper presented at the Co‐operatives and the World of Work International Research Conference, Antalya, 9 November. https://www.academia.edu/25395795/_Coffee_has_given_us_voice_Coffee_Cooperatives_and_Women_empowerment_in_Rwandas_rural_areas

[cl21428-bib-0114] Holmes, H. , & Imai, K. (2019). *Fair trade and wellbeing improvements: Evidence from Sri Lanka* (Working Paper, 2019–040). Global Development Institute (GDI).

[cl21428-bib-0115] Mojo, D. , Fischer, C. , & Degefa, T. (2017). The determinants and economic impacts of membership in coffee farmer cooperatives: Recent evidence from rural Ethiopia. Journal of Rural studies, 50, 84–94.

[cl21428-bib-0116] Namayengo, M. M. F. (2017). *Microcredit to women and its contribution to production and household food security* [Doctoral dissertation, Wageningen University].

[cl21428-bib-0117] Quisumbing, A. R. , Roy, S. , Njuki, J. , Tanvin, K. , & Waithanji, E. (2013). *Can dairy value‐chain projects change gender norms in rural Bangladesh? Impacts on assets, gender norms, and time use* (Discussion Papers, No.1311). IFPRI.

[cl21428-bib-0118] Abtew, A. , Pretzsch, J. , Secco, L. , & Mohamod, T. (2014). Contribution of small‐scale gum and resin commercialization to local livelihood and rural economic development in the drylands of eastern Africa. Forests, 5(5), 952–977.

[cl21428-bib-0119] Bachmann, F. (2012). Potential and limitations of organic and fair trade cotton for improving livelihoods of smallholders: Evidence from Central Asia. Renewable Agriculture and Food Systems, 27(2), 138–147.

[cl21428-bib-0120] Kikulwe, E. M. , Kyanjo, J. L. , Kato, E. , Ssali, R. T. , Erima, R. , Mpiira, S. , Ocimati, W. , Tinzaara, W. , Kubiriba, J. , Gotor, E. , Stoian, D. , & Karamura, E. (2019). Management of Banana Xanthomonas Wilt: Evidence from impact of adoption of cultural control practices in Uganda. Sustainability, 11(9), 2610.

[cl21428-bib-0121] Kolapo, A. , Kolapo, A. J. , & Yildiz, F. (2021). Welfare and productivity impact of adoption of biofortified cassava by smallholder farmers in Nigeria. Cogent Food & and Agriculture, 7, 1886662.

[cl21428-bib-0122] Larson, D. F. , Savastano, S. , Murray, S. , & Palacios‐López, A. (2015). *Are women less productive farmers? How markets and risk affect fertilizer use, productivity, and measured gender effects in Uganda* (Policy Research Working Paper No.7241). World Bank.

[cl21428-bib-0123] Madheswaran, S. , & Dharmadhikary, A. (2001). Empowering rural women through self‐help groups: Lessons from Maharashtra Rural Credit Project. Indian Journal of Agricultural Economics, 56(3), 427–443.

[cl21428-bib-0124] Nikiema, R. A. , & Sakurai, T. (2021). Intrahousehold distribution of sales revenue and household nutritional outcomes: What if the wives controlled the farm revenue? Agricultural Economics, 52(6), 1029–1040.

[cl21428-bib-0125] Ojo, T. O. , Baiyegunhi, L. J. S. , Adetoro, A. A. , & Ogundeji, A. A. (2021). Adoption of soil and water conservation technology and its effect on the productivity of smallholder rice farmers in Southwest Nigeria. Heliyon, 7(3), e06433. 33763609 10.1016/j.heliyon.2021.e06433 PMC7973869

[cl21428-bib-0126] Resende Filho, M. A. , Andow, D. A. , Carneiro, R. G. , Lorena, D. R. , Sujii, E. R. , & Alves, R. T. (2019). Economic and productivity incentives to produce organically in Brazil: Evidence from strawberry production in the Federal District. Renewable Agriculture and Food Systems, 34(2), 155–168.

[cl21428-bib-0127] Thapa, G. , Kumar, A. , & Joshi, P. K. (2017). *Agricultural diversification in Nepal: Status, determinants, and its impact on rural poverty* (Discussion Papers, No.1634). IFPRI.

[cl21428-bib-0128] Van Rijn, F. C. (2014). *Social capital, agricultural innovation and the evaluation of agricultural development initiatives* [Doctoral thesis, Wageningen University].

[cl21428-bib-0129] Wang, H. , Wang, X. , Sarkar, A. , & Qian, L. (2021a). Evaluating the impacts of smallholder farmer's participation in modern agricultural value chain tactics for facilitating poverty alleviation – A case study of kiwifruit industry in Shaanxi, China. Agriculture, 11(5), 462.

[cl21428-bib-0130] Wang, X. , Sarkar, A. , Wang, H. , & Zhang, F. (2021b). Does participation in agricultural value chain activities influence smallholder fruit grower production performance? A cross‐sectional study of apple farmers in Shandong, China. Horticulturae, 7(6), 153.

[cl21428-bib-0131] Zhao, R. , Qiu, X. , & Chen, S. (2021). Empirical study on the effects of technology training on the forest‐related income of rural poverty‐stricken households – based on the PSM method. Sustainability, 13(13), 7143.

[cl21428-bib-0132] Agarwal, B. (2018). Gender equality, food security and the sustainable development goals. Current Opinion in Environmental Sustainability, 34, 26–32.

[cl21428-bib-0133] Barrett, C. B. , Reardon, T. , Swinnen, J. , & Zilberman, D. (2022). Agri‐food value chain revolutions in low‐ and middle‐income countries. Journal of Economic Literature, 60(4), 1316–1377.

[cl21428-bib-0134] De Brauw, A. , & Bulte, E. (2021). African *farmers, value chains, and agricultural development* . Palgrave Macmillan.

[cl21428-bib-0135] Carvalho, S. , & White, H. , 1997. *Combining the quantitative and qualitative approaches to poverty measurement and analysis: The practice and the potential* (Technical Paper 366). World Bank.

[cl21428-bib-0136] Coles, C. , & Mitchell, J. (2011). Gender and agricultural value chains: A review of current knowledge and practice and their policy implications.

[cl21428-bib-0137] Doss, C. , & SOFA Team . (2011). *The role of women in agriculture*. Food and Agriculture Organization of the United Nations (FAO).

[cl21428-bib-0138] Eyben, R. , Kabeer, N. , & Cornwall, A. (2008). Conceptualising empowerment and the implications for pro poor growth. DAC Poverty Network by the Institute of Development Studies.

[cl21428-bib-0139] Food and Agriculture Organization of the United Nations (FAO) . (2016). Developing gender‐sensitive value chains: A guiding framework. FAO.

[cl21428-bib-0140] Gibbs, A. , Willan, S. , Misselhorn, A. , & Mangoma, J. (2012). Combined structural interventions for gender equality and livelihood security: A critical review of the evidence from southern and eastern Africa and the implications for young people. Journal of the International AIDS Society, 15(S1), 1–10. 10.7448/IAS.15.3.17362 PMC3499786 22713350

[cl21428-bib-0141] Golla, A. M. , Malhotra, A. , Nanda, P. , & Mehra, R. (2011). Definition, framework and indicators. International Center for Research on Women (ICRW).

[cl21428-bib-0142] Haggblade, S. , Theriault, V. , Staatz, J. , Dembele, N. , & Diallo, B. (2012). A conceptual framework for promoting inclusive agricultural value chains improving the inclusiveness of agricultural value chains in West Africa. International Fund for Agricultural Development (IFAD).

[cl21428-bib-0143] Higgins, J. P. T. , Thomas, J. , Chandler, J. , Cumpston, M. , Li, T. , Page, M. , & Welch, A. V. (2011). *Cochrane handbook for systematic reviews of interventions. Version 5.1.0*. The Cochrane Collaboration. http://handbook-5-1.cochrane.org

[cl21428-bib-0145] Ihalainen, M. , Shaikh, S. , Mujawamariya, G. , Mayanja, S. , Adetonah, S. , Tavenner, K. , & Elias, M. (2021). Promise and contradiction: Value chain participation and women's empowerment. In R. Pyburn , & A. van Eerdewijk (Eds.), Advancing gender equality through agricultural and environmental research: Past, present and future (pp. 147–188). International Food Policy Research Institute (IFPRI). 10.2499/9780896293915_04

[cl21428-bib-0146] Interagency Gender Working Group . (2017). The gender integration continuum: Training session user's guide. Population Reference Bureau, United States Agency for International Development (USAID).

[cl21428-bib-0147] International Finance Corporation (IFC) . (2016). Investing in women along agribusiness value chains. IFC.

[cl21428-bib-0148] Kabeer, N. , & Natali, L. (2013). Gender equality and economic growth: Is there a win‐win? IDS Working Papers, 2013(417), 1–58.

[cl21428-bib-0149] Kaplinsky, R. , & Morris, M. (2000). A handbook for value chain research (Vol. 113). University of Sussex, Institute of Development Studies.

[cl21428-bib-0150] Kidder, T. , Romana, S. , Canepa, C. , Chettleborough, J. , & Molina, C. (2017). Oxfam's conceptual framework on women's economic empowerment. Oxfam.

[cl21428-bib-0151] KIT, Faida MaLi, & IIRR . (2006). *Chain empowerment: Supporting African farmers to develop markets*. Amsterdam, Netherlands: Royal Tropical Institute, Arusha, Tanzania: Faida Market Link and Nairobi, Kenya: International Institute of Rural Reconstruction.

[cl21428-bib-0152] Mayoux, L. , & Mackie, G. (2008). A practical guide to mainstreaming gender analysis in value chain development. ILO.

[cl21428-bib-0153] McKague, K. , & Siddiquee, M. (2014). Value chain intervention strategies. In Making markets more inclusive: Lessons from CARE and the future of sustainability in agricultural value chain development (pp. 47–55). Palgrave Macmillan US.

[cl21428-bib-0154] Meinzen‐Dick, R. S. , Johnson, N. L. , Quisumbing, A. R. , Njuki, J. , Behrman, J. , Rubin, D. , & Waithanji, E. M. (2011). Gender, assets, and agricultural development programs: A conceptual framework. CAPRi Working Paper.

[cl21428-bib-0155] Morioka, K. , & Nicholas, G. (2014). Women's empowerment and value chains: Experiences of women in Cambodia, Palestine and Uganda. Action Aid.

[cl21428-bib-0156] Mutua, E. , Njuki, J. , & Waithanji, E. (2014). Review of gender and value chain analysis, development and evaluation toolkits. International Livestock Research Institute (ILRI).

[cl21428-bib-0157] Neven, D. (2014). *Developing sustainable food value chains: Guiding principles*. Food and Agriculture Organization of the United Nations (FAO).

[cl21428-bib-0158] Okesina, M. (2020). A framework for assessing women's economic empowerment. *Global Journal of Arts* . Humanities and Social Sciences, 8(6), 1–17.

[cl21428-bib-0159] Porter, M. (1985). The value chain and competitive advantage. In Competitive advantage: Creating and sustaining superior performance (pp. 33–61). Free Press.

[cl21428-bib-0160] Powercube . (2013). *Understanding power for social change*. http://www.powercube.net

[cl21428-bib-0161] Pyburn, R. , & van Eerdewijk, A. (2021). Advancing gender equality through agricultural and environmental research: Past, present, and future. International Food Policy Research Institute (IFPRI).

[cl21428-bib-0162] Pyburn, R. , & Kruijssen, F. (2020). Gender dynamics in agricultural value chain development: Foundations and gaps. In Routledge handbook of gender and agriculture (pp. 32–45). Routledge.

[cl21428-bib-0400] Raney, T. , Croppenstedt, A. , Anriquez, G. , & Lowder, S. (2011). The state of food and agriculture 2010‐11: Women in agriculture: Closing the gender gap for development. Food and Agriculture Organization.

[cl21428-bib-0163] Riisgaard, L. , Fibla, A. M. , & Ponte, S. (2010). Evaluation study: Gender and value chain development. The Evaluation Department of the Danish Foreign Ministry.

[cl21428-bib-0164] Rubin, D. , Manfre, C. , & Barett, K. N. (2010). Promoting gender equitable opportunities in agriculture value chains: A handbook. USAID Office of Women in Development.

[cl21428-bib-0165] Senders, A. , Lentink, A. , Vanderschaeghe, M. , & Terrillion, J. (2012). Gender in value chains: Practical toolkit to integrate a gender perspective in agricultural value chain development. Agri‐ProFocus Learning Network.

[cl21428-bib-0166] Stamm, A. , & Von Drachenfels, C. (2011). Value chain development: Approaches and activities by seven UN agencies and opportunities for interagency cooperation. International Labour Organisation (ILO).

[cl21428-bib-0167] Stewart, R. , Van Rooyen, C. , Korth, M. , Chereni, A. , Da Silva, N. R. , & De Wet, T. (2012). Do micro‐credit, micro‐savings and micro‐leasing serve as effective financial inclusion interventions enabling poor people, and especially women, to engage in meaningful economic opportunities in low‐ and middle‐income countries? A systematic review of the evidence. EPPI‐Centre.

[cl21428-bib-0168] Stoian, D. , Donovan, J. , Elias, M. , & Blare, T. (2018). Fit for purpose? A review of guides for gender‐equitable value chain development. Development in Practice, 28(4), 494–509.

[cl21428-bib-0170] Ugwu, P. (2019). *Women in agriculture: Challenges facing women in African farming. Project report of African Women in Agriculture* (Research report: Postgraduate School of Agricultural and Food Economics, Catholic University of the Sacred Heart, Italy).

[cl21428-bib-0171] Webber, C. M. , & Labaste, P. (2010). Building competitiveness in Africa's agriculture: A guide to value chain concepts and applications. World Bank.

[cl21428-bib-0172] White, H. (2018). Theory‐based systematic reviews. Journal of Development Effectiveness, 10(1), 17–38.

