# Supplementary material for: Value chain interventions for improving women's economic empowerment: A mixed‐methods systematic review and meta‐analysis: A systematic review
Source: Campbell Syst Rev. 2024 Aug 12;20(3):e1428. doi: 10.1002/cl2.1428 (PMC11317815; doi:10.1002/cl2.1428)
Supplement: Supplementary file 1 — Supporting information. [file CL2-20-e1428-s001.docx]

**Abbreviations and acronyms**

**CI** Confidence interval

**FAO** Food and Agriculture Organisation

**SMD** Standardised mean difference

SSCI Social Sciences Citation Index

CPCI-S Conference Proceedings Citation Index – Science

CPCI-SSH Conference Proceedings Citation Index - Social Science & Humanities

ESCI Emerging Sources Citation Index

CGIAR Consultative Group on International Agricultural Research

IFAD International Fund for Agricultural Development

BMGF Bill & Melinda Gates Foundation

FAO UN Food and Agriculture Organisation

ILO International Labour Organisation

BLDS British Library for Development Studies

IPA Innovations for Poverty Action

CSAE Centre for the Study of African Economies

NEUDC [North East Universities Development Consortium](https://www.neudc.org/)

ToC Theory of change

MLT Middle- level theory

# Online supplements

**Appendix A: Search terms**

| 1  Agriculture | TITLE-ABS-KEY("smallhold*" OR "small hold*" OR "microfarm*" OR "micro-farm*" OR "pastoral*" OR "agropastoral" OR "agro-pastoral" OR "ejido" OR "silvopastoral" OR "farm*" OR "agricultur*" OR "producer*" OR "grower*" OR "agronomy" OR "husbandry" OR "aquacultur*" OR "floricultur*" OR "horticultur*" OR "cultivat*" OR "dairy" OR "livestock" OR "crop*") | 2,070,995 |
| --- | --- | --- |
| 2  Value chain | TITLE-ABS-KEY(fairtrade OR "fair trade" OR "contract farming" OR "post-harvest management" OR "making markets work" OR "market system*" OR "Participatory Market Chain Approach" OR "market development*" OR "market intervention*" OR "agricultural product market*" OR "supply chain*" OR "production to consumption system*" OR "farmers’ based organisations" OR "farmers' based organization*" OR "farmer based organization*" OR "farmer based organization" OR "farmers' group*" OR "farmers group*" OR "cooperative*" OR "value-add*" OR "value-chain*" OR "market modernization" OR "market modernisation" OR "modern market*" OR " marketing channel*" OR "commercialization channel*" OR "commercialisation channel*" OR "high-value chain*" OR "high-value market*" OR "agrifood transformation*" OR "agri-food transformation*" OR "agrifood system*" OR "agri-food system*" OR "agrifood chain*" OR "agri-food chain*" OR "food industry" OR "food sector" OR "food system" OR "e-commerce" OR "cold chain" OR "wholesale market" OR "market reform*" OR "market linkage*" OR "commodity chain*" OR "commodities chain*" OR "handicraft*" OR "artisan*" OR "self-help group*" OR "public-private partnership*" OR "small and medium-size* enterprise*" OR SMEs OR "small enterprise*" OR "rural enterprise*" OR "micro-enterprise*" OR "microenterprise*") OR TITLE-ABS-KEY((vertical OR horizontal) W/2 ("integration" OR "coordination" OR "linkage*")) | 763,607 |
| 3  Value chain | TITLE-ABS-KEY(("crop" OR "crops" OR "food" OR "produce" OR "agri-product*" OR "agro-food" OR "tuber*" OR "root crop*" OR "barley" OR "oat*" OR "rye" OR "wheat" OR "arrowroot" OR "artichoke*" OR "banana*" OR "yam*" OR "breadfruit" OR "chickpea*" OR "lentil*" OR "pea*" OR "bean*" OR "potato*" OR "cassava*" OR "millet" OR "rice" OR "amaranth" OR "paddy" OR "maize" OR "sorghum" OR "corn" OR "cashew*" OR "meat" OR "fish" OR "vegetable*" OR "chicken" OR "turkey" OR "duck" OR "fruit" OR "staple crop*" OR "cash crop*" OR "rubber" OR "plantain*" OR "sugarcane" OR "timber" OR "cotton" OR "coffee" OR "tea" OR "bean*" OR "legume*" OR "spice*" OR "livestock" OR "pork" OR "poultry" OR "shrimp" OR "cattle" OR "cow*" OR "beef" OR "pig*" OR "goat*" OR "sheep" OR "milk" OR "dairy" OR "tomato*" OR "carrot*" OR "onion*" OR "cauliflower" OR "grain*" OR "cereal" OR "soybean*" OR "peanut*" OR "oilseed*" OR "citrus" OR "fodder" OR "hay" OR "silage" OR "forage" OR "palm" OR "melon*" OR "avocado*" OR "flower*" OR "pulse*" OR "ground nut*" OR "egg*" OR "strawberr*" OR "currant*" OR "*berry" OR "*berries" OR "mango*" OR "guava*" OR "papaya*" OR "pawpaw" OR "paw-paw" OR "orange*" OR "lemon*" OR "spinach" OR "lettuce" OR "mushroom*" OR "pepper*" OR "microgreen*") W/3 ("processing" OR "packaging" OR "trade" OR "trading" OR "retail*" OR "transport*" OR "distribution" OR "storage" OR "storing" OR "branding" OR organic OR certification)) | 394,316 |
| 4 | #2 OR #3 | 1,127,405 |
| 5  Empowerment | TITLE-ABS-KEY(empower* OR disempower* OR autonomy OR (decision* W/2 (make OR made OR making) )) OR TITLE-ABS-KEY(self-determin* OR bargain* OR negotiat* OR equal* OR agency OR transformati* OR particip* OR engag* OR inclus* OR represen* OR access* OR equit* OR inequit* OR inequalit* OR disadvantage* OR marginali* OR discriminat* OR vulnerab* OR "self help" OR ownership* OR power* OR norm OR norms OR poverty OR economic*) | 18,532,952 |
| 6  Women | TITLE-ABS-KEY(gender* or woman* or women* or mother* or maternal or female* or wife* or wives) | 11,866,371 |
| 7  Low- and middle-income country | TITLE-ABS-KEY(afghanistan or albania or algeria or "american samoa" or angola or "antigua and barbuda" or antigua or barbuda or argentina or armenia or armenian or aruba or azerbaijan or bahrain or bangladesh or barbados or belarus or byelarus or belorussia or byelorussian or belize or "british honduras" or benin or dahomey or bhutan or bolivia or bosnia or herzegovina or botswana or bechuanaland or brazil or brasil or bulgaria or "burkina faso" or "burkina fasso" or "upper volta" or burundi or urundi or "cabo verde" or "cape verde" or cambodia or kampuchea or "khmer republic" or cameroon or cameron or cameroun or "central african republic" or "ubangi shari" or chad or chile or china or colombia or comoros or "comoro islands" or "iles comores" or mayotte or congo or zaire or "costa rica" or "cote d ivoire" or "cote divoire" or "cote d ivoire" or "ivory coast" or croatia or cuba or cyprus or "czech republic" or czechoslovakia or djibouti or "french somaliland" or dominica or "dominican republic" or ecuador or egypt or "united arab republic" or "el salvador" or eritrea or estonia or eswatini or swaziland or ethiopia or fiji or gabon or "gabonese republic" or gambia or "georgia (republic)" or georgian or ghana or "gold coast" or gibraltar or greece or grenada or guam or guatemala or guinea or guyana or "british guiana" or haiti or hispaniola or honduras or hungary or india or indonesia or timor or iran or iraq or "isle of man" or jamaica or jordan or kazakhstan or kazakh or kenya or korea or kosovo or kyrgyzstan or kirghizia or kirgizstan or "kyrgyz republic" or kirghiz or laos or "lao pdr" or "lao people's democratic republic" or latvia or lebanon or "lebanese republic" or lesotho or basutoland or liberia or libya or "libyan arab jamahiriya" or lithuania or macau or macao or macedonia or madagascar or "malagasy republic" or malawi or nyasaland or malaysia or "malay federation" or "malaya federation" or maldives or "indian ocean" or mali or malta or micronesia or kiribati or "marshall islands" or nauru or "northern mariana islands" or palau or tuvalu or mauritania or mauritius or mexico or moldova or moldovian or mongolia or montenegro or morocco or ifni or mozambique or "portuguese east africa" or myanmar or burma or namibia or nepal or "netherlands antilles" or nicaragua or niger or nigeria or oman or muscat or pakistan or panama or "papua new guinea" or "new guinea" or paraguay or peru or philippines or philipines or phillipines or phillippines or poland or "polish people's republic" or portugal or "portuguese republic" or "puerto rico" or romania or russia or "russian federation" or ussr or "soviet union" or "union of soviet socialist republics" or rwanda or ruanda or samoa or "pacific islands" or polynesia or "samoan islands" or "navigator island" or "navigator islands" or "sao tome and principe" or "saudi arabia" or senegal or serbia or seychelles or "sierra leone" or slovakia or "slovak republic" or slovenia or melanesia or "solomon island" or "solomon islands" or "norfolk island" or "norfolk islands" or somalia or "south africa" or "south sudan" or "sri lanka" or ceylon or "saint kitts and nevis" or "st. kitts and nevis" or "saint lucia" or "st. lucia" or "saint vincent and the grenadines" or "saint vincent" or "st. vincent" or grenadines or sudan or suriname or surinam or "dutch guiana" or "netherlands guiana" or syria or "syrian arab republic" or tajikistan or tadjikistan or tadzhikistan or tadzhik or tanzania or tanganyika or thailand or siam or "timor leste" or "east timor" or togo or "togolese republic" or tonga or "trinidad and tobago" or trinidad or tobago or tunisia or turkey or turkmenistan or turkmen or uganda or ukraine or uruguay or uzbekistan or uzbek or vanuatu or "new hebrides" or venezuela or vietnam or "viet nam" or "middle east" or "west bank" or gaza or palestine or yemen or yugoslavia or zambia or zimbabwe or "northern rhodesia" or "global south" or "africa south of the sahara" or "sub-saharan africa" or "subsaharan africa" or "africa, central" or "central africa" or "africa, northern" or "north africa" or "northern africa" or magreb or maghrib or sahara or "africa, southern" or "southern africa" or "africa, eastern" or "east africa" or "eastern africa" or "africa, western" or "west africa" or "western africa" or "west indies" or "indian ocean islands" or caribbean or "central america" or "latin america" or "south and central america" or "south america" or "asia, central" or "central asia" or "asia, northern" or "north asia" or "northern asia" or "asia, southeastern" or "southeastern asia" or "south eastern asia" or "southeast asia" or "south east asia" or "asia, western" or "western asia" or "europe, eastern" or "east europe" or "eastern europe") | 6,163,381 |
| 8  Low- and middle-income country | TITLE-ABS-KEY ("developing country" or "developing countries" or "developing nation*" or "developing population*" or "developing world" or "less developed countr*" or "less developed nation*" or "less developed population*" or "less developed world" or "lesser developed countr*" or "lesser developed nation*" or "lesser developed population*" or "lesser developed world" or "under developed countr*" or "under developed nation*" or "under developed population*" or "under developed world" or "underdeveloped countr*" or "underdeveloped nation*" or "underdeveloped population*" or "underdeveloped world" or "middle income countr*" or "middle income nation*" or "middle income population*" or "low income countr*" or "low income nation*" or "low income population*" or "lower income countr*" or "lower income nation*" or "lower income population*" or "underserved countr*" or "underserved nation*" or "underserved population*" or "underserved world" or "under served countr*" or "under served nation*" or "under served population*" or "under served world" or "deprived countr*" or "deprived nation*" or "deprived population*" or "deprived world" or "poor countr*" or "poor nation*" or "poor population*" or "poor world" or "poorer countr*" or "poorer nation*" or "poorer population*" or "poorer world" or "developing econom*" or "less developed econom*" or "lesser developed econom*" or "under developed econom*" or "underdeveloped econom*" or "middle income econom*" or "low income econom*" or "lower income econom*" or "low gdp" or "low gnp" or "low gross domestic" or "low gross national" or "lower gdp" or "lower gnp" or "lower gross domestic" or "lower gross national" or lmic or lmics or "third world" or "lami countr*" or "transitional countr*" or "emerging economies" or "emerging nation*") | 412,090 |
| 9  Low- and middle-income country | #7 OR #8 | 6,349,458 |
| 10  All combined concepts | #1 AND #4 AND #5 AND #6 AND #9 | 2,657 |
| 11  Optional study design hedge | TITLE-ABS-KEY("random* control* trial*" OR "random* trial*" OR rct* OR (random* W/3 allocat*) OR "quasi experiment*" OR quasi-experiment* OR experiment* OR evaluat* OR impact* OR assess* OR multi-level OR multilevel OR "multi level" OR dif-dif OR psm OR "double difference" OR difference-in-difference OR rdd OR "difference in difference" OR "double difference" OR "statistical matching*" OR "propensity score matching" OR "covariate matching" OR "coarsened-exact matching" OR propensity-weighted OR "regression" OR "cohort analysis" OR "cross-section*" OR "panel" OR group* OR compar* OR (quantitative W/2 (method* OR study OR design OR analysis)) OR "interrupted time series" OR (("fixed effect*" OR "random effect*") W/3 (model OR estimation)) OR "instrumental variable" OR ((cost* OR economic) W/2 (benefit or effective* or analy*))) | 38,234,488 |
| 12  With study design hedge | #10 AND #11 | 1,967 |

**Appendix B: Screening tool**

**Screening tool for Systematic Review 3 (VCIA and women’s empowerment)**

1. Is the study conducted in low- and middle-income countries, as per the latest World Bank classification?

- Yes, include and see Question 2
- No, exclude on **country**

1. Does the study target women or other stakeholders such as smallholder farmers, value chain entrepreneurs, or employees engaged in agricultural and food systems, particularly in value chain development and market engagement interventions?

- Yes, include and see Question 3
- No, exclude on **population**

1. Does the study evaluate an intervention (policy, programme, project or practice), or a review of evaluations of an intervention aimed at empowering women through value chain development and market engagement interventions?

- Yes, include and see Question 4
- No, exclude on **intervention**

1. Does the study analyse the effect of the intervention on the empowerment of women (this may include measures such as encouraging or enabling women to participate, lead and become members of farmers’ organisations)?

- Yes, include and see Question 5
- No, exclude on **outcome**

1. Does the study have an experimental or non-experimental design with comparison group, instrumental variables and interrupted time series?

- Yes, include for effectiveness and Stop!
- No, see Question 6

1. Does the study evaluate a programme/policy for the empowerment of women in agricultural value chains and discuss implementation issues?

- Yes, include for process evaluation
- No, exclude on **design**

**Appendix C: Coding tools**

**CODING TOOL**

| **Category** | **Subcategory** |
| --- | --- |
| **Publication status** | - Ongoing - Completed |
| **Region** | - East Asia & the Pacific - Europe & Central Asia - Latin America & the Caribbean - Middle East & North Africa - South Asia - Sub-Saharan Africa |
| **Country name** |  |
| **Settings** | - Rural - Urban - Rural and urban (both) - Not clear |
| **Project/ Intervention name** |  |
| **Year** |  |
| **Funding agency** |  |
| **Implementing agency** |  |
| **Duration of intervention** |  |
| **Unit of delivery** | - Individual: one to one - Group |
| **Gender** | - Male - Female - Non-binary - All sexes - Not reported |
| **BAME** | - Mainly/exclusively (80%) - Partly - None - Not clear |
| **Study design** | - Experimental design - Non-experimental design - Process evaluation - Cost analysis |
| **Study method** | - Randomised controlled trial - Difference in difference - Instrumental variable estimation - Regression discontinuity design - Statistical matching (PSM) - Interrupted time series - Fixed effects estimation |
| **Mixed _method** | - Yes - No |
| **Intervention category** | **Intervention subcategory** |
| **Value chain development** | - Enabling policies and institutional environment - Financial services (including both grants and subsidies; and micro-credit, savings and insurance), - Processing and storage facilities - Horizontal and vertical coordination - Process, product, and chain upgrading - Enterprise development and impact investing - Promoting the production of a new profitable product - Improving product market quality (fair trade, organic farming, and quality standards) - Supporting horizontal integration of producers group to access better prices - Improving processing techniques - Contract farming |
| **Market engagement** | - Inclusive market systems development - Gender-friendly markets (including lighting, washroom facilities, provision for childcare) - Access to markets (through farm-to-market roads, transport facilities) - Market structures |
| **Outcome domain** | **Outcome subdomain** |
| **Economic empowerment** | - Decision-making on value chain activities - Decision-making over the use of income - Increased bargaining power - Leadership positions in groups |
| **Economic benefits** | - Farm productivity - Income - Time use - Assets ownership |
| **Participation** | - Access to information on production/markets - Enhanced social and institutional networks - Increased participation in paid labour opportunities - Access to new markets - Knowledge and skills - Gender roles and norms |
| **Effect sizes calculation** |  |
| **Economic empowerment** |  |
| **Economic benefits** |  |
| **Participation** |  |
| **Attrition** |  |
| **Differential attrition** |  |
| **Barriers and facilitators to participation** |  |
| **Barriers and facilitators to outcome** |  |
| **Design issues** |  |
| **Implementation issues** |  |
| **What target populations say** |  |
| **Moderators and confounders** |  |

**Appendix D: Critical appraisal tool**

The critical appraisal tool helped reviewers to indicate the quality of the confidence in findings included in the review. All studies were rated against how clear the intervention and evaluation questions described in the study were, and overall scores were also calculated in the same way.

For more a more detailed look at study quality, separate questions were considered for impact and process evaluations because they have different purposes and therefore different elements that can affect their quality.

The tool can be found below and was developed in conjunction with the Campbell Collaboration as well as our partners at the Early Intervention Foundation. It was created by consulting other quality tools available (namely the Critical Appraisal Skills Programme Checklist25) and seeking further input from partners at the Foundation, as well as experts in the field.

| *Critical appraisal tool for primary studies: effectiveness* | | | |
| --- | --- | --- | --- |
| Item | **Description** | **Key** | **Notes** |
| **Intervention** | Is the intervention clearly named and described, including all relevant components? | High: full and clear description, so that the main components and how they are delivered are clear  Medium: Partial description  Low: Little or no description |  |
| **Evaluation questions** | Are the evaluation questions clearly stated? | High: full and clear description, so that the main components and how they are delivered are clear  Medium: Partial description  Low: Little or no description |  |
| **Study design** | Use the study design coding | High: Experimental  Medium: Non-experimental  Low: Before versus after |  |
| **Outcomes** | Are the outcomes clearly defined? Where appropriate, do they use an existing, validated measurement tool? | High: full and clear definition using validated instruments where available (a researcher wishing to use these outcomes would have sufficient information to do so)  Medium: Partial definition. May use validated instruments but without sufficient references to source.  Low: Little or no definition |  |
| **Sample size (power calculation)** | Do the authors report a power calculation as the basis for sample size? | High: Power calculation report and sample size meets necessary sample size  Medium: Power calculation mentioned, and sample size meets necessary sample size  Low: No mention of power calculation |  |
| **Attrition** | Reported for endline and longest follow up  Calculate overall attrition and differential attrition  It is often necessary to calculate from the table of results. If sample size varies by outcome, calculate for highest attrition. | High: Attrition within IES conservative standard  Medium: Attrition within IES liberal standard  Low: Attrition outside IES liberal standard |  |
| **Overall (including questions for all studies)** | The overall score uses the weakest link in the chain principle (i.e., is the lowest score on any item) | High: High on all items  Medium: No lower than medium on any item  Low: At least one low |  |

**Critical appraisal tool – Process evaluation**

**Questions for process evaluations (apply to implementation sections) (used for any study coded as having implementation evidence)**

|  |  | High | Medium | Low |  | Low |
| --- | --- | --- | --- | --- | --- | --- |
| 1 | Is the qualitative methodology described? | Yes |  | No | >> 3 |  |
| 2 | Is the qualitative methodology appropriate to address the evaluation questions? | Yes | Partially | No |  | Insufficient detail |
| 3 | Is the recruitment or sampling strategy described? | Yes |  | No | >> 5 |  |
| 4 | Is the recruitment or sampling strategy appropriate to address the evaluation questions? | Yes | Partially | No |  | Insufficient detail |
| 5 | Are the researcher’s own position, assumptions and possible biases outlined? | Yes | Partially | No |  |  |
| 6 | Have ethical considerations been sufficiently considered? | Yes | Partially | No |  | Insufficient detail |
| 7 | Is the data analysis approach adequately described? | Yes |  | No | >>9 |  |
| 8 | Is the data analysis sufficiently rigorous? | Yes | Partially | No |  |  |
| 9 | Are the implications or recommendations clearly based on the evidence from the study? | Yes | Partially | No |  |  |
| 10 | Overall (including questions for all studies)  The overall score uses the weakest link in the chain principle (i.e., is the lowest score on any item) | High: High on all items  Medium: No lower than medium on any item  Low: At least one low |  |  |  |  |

**Appendix E: Definitions of outcomes**

| **Categories** | **Subcategories** | **Definition** |
| --- | --- | --- |
| Economic Empowerment | Decision-making on value chain activities | Women’s decision-making power (e.g., over agricultural production, income, or household food consumption); reduction of outcomes associated with disempowerment (e.g., gender-based violence, time burden) |
|  | Decision-making over the use of income |  |
|  | Increased bargaining power | Women’s bargaining power in negotiating and managing vertical and horizontal relationships |
|  | Leadership positions in groups | Membership in economic or social groups and comfort with speaking in public |
|  | Mobility | Increase in women’s mobility to spaces considered as male. Spaces to be gender neutral where information is exchanged.  The environment that restricts women’s mobility reduces their ability to engage in networking opportunities and limits the information available to them. |
| Economic benefits | Farm productivity | Increase in farm productivity |
|  | Income | Increased in the income of the household/Individual |
|  | Assets ownership | Ownership, access to, and decision-making power over productive resources such as land, livestock, agricultural equipment, consumer durables, and credit |
| Participation | Access to information on production/markets | Access to information about new markets, production processes |
|  | Enhanced social and institutional networks | Increased ability to develop social capital and challenge social constraints  Reduced social pressure for women to conform to stereotypes  Reduced violence in the household  Reduced pressure for conformity to social norms  Financial Institution |
|  | Increased participation in paid labour opportunities/ labour participation | Women are considered for all skilled positions in production processes, as well as in firms engaged in export and marketing. |
|  | Access to new markets | Access to new markets by women and men |
|  | Knowledge and skills | Increase in knowledge and skills |
|  | Gender roles and norms | Influence gender roles and norms  Change in gender roles and norms |
| Time use | Workload | Allocation of time to productive and domestic tasks |
|  | Leisure | Satisfaction with available time for leisure activities |

**Appendix F: Moderator analysis – forest plots**

- - - 1. **Geographical, by region**


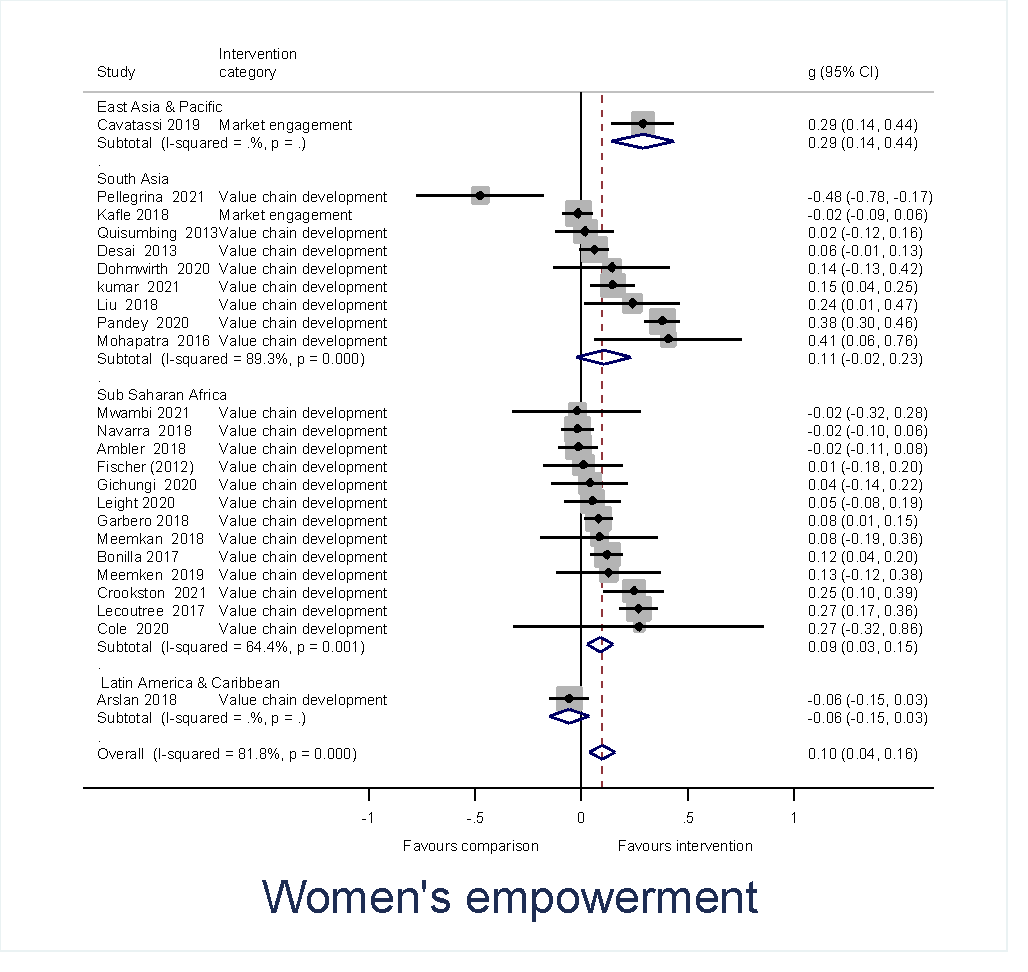


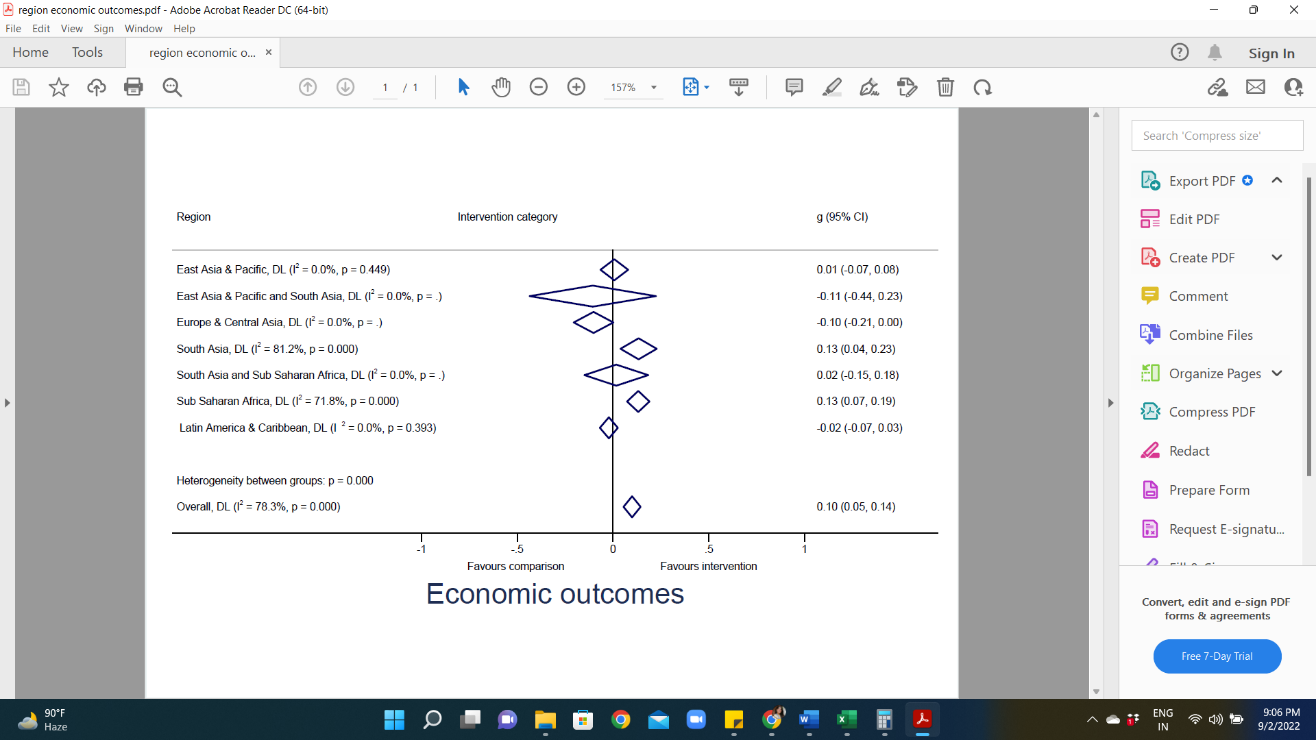


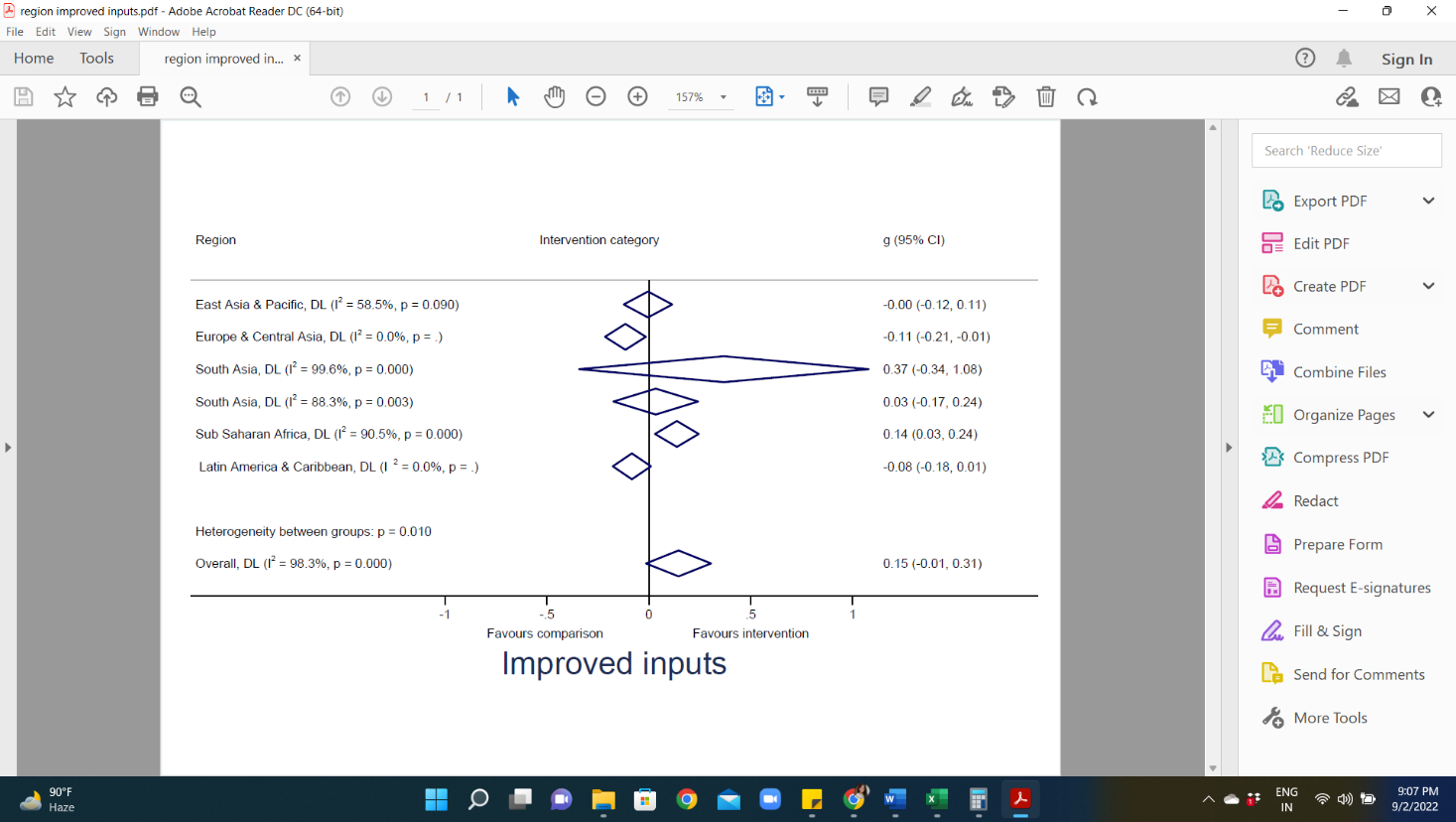

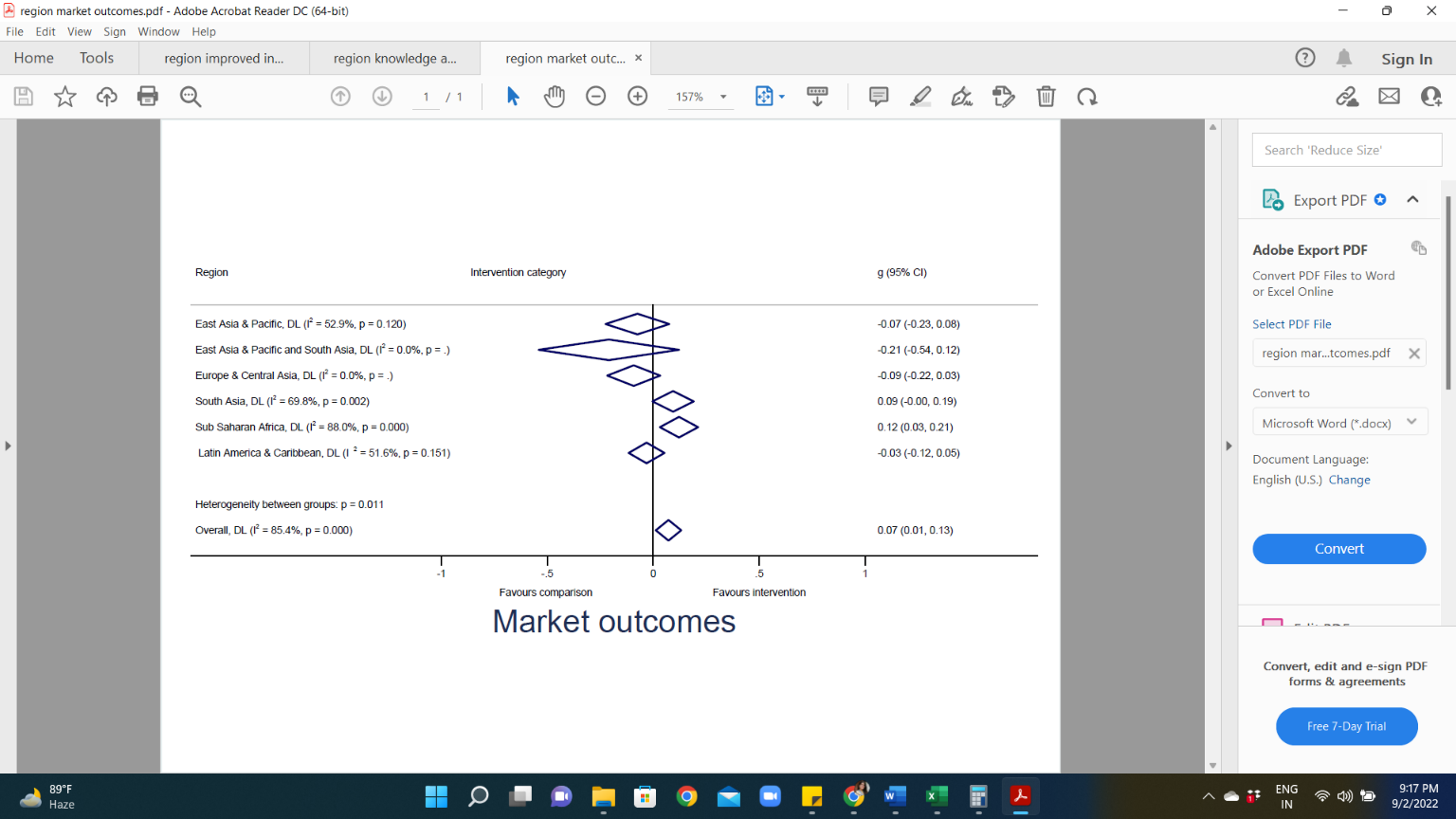

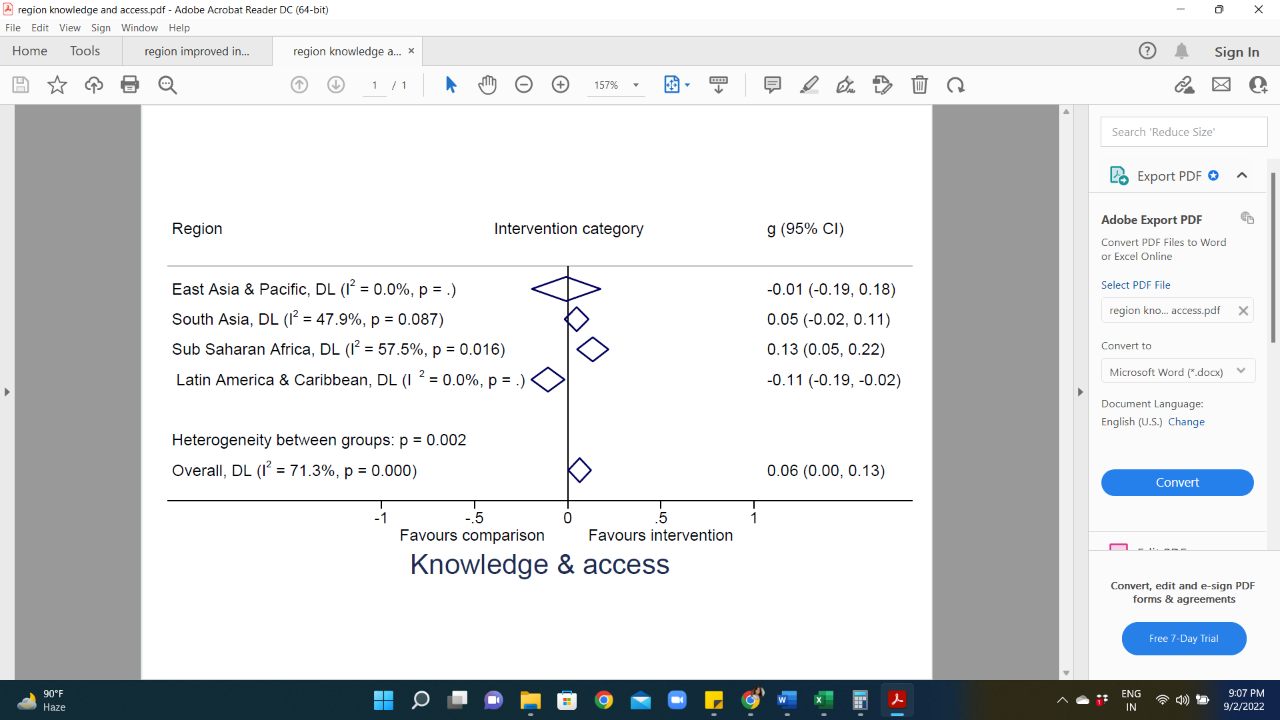


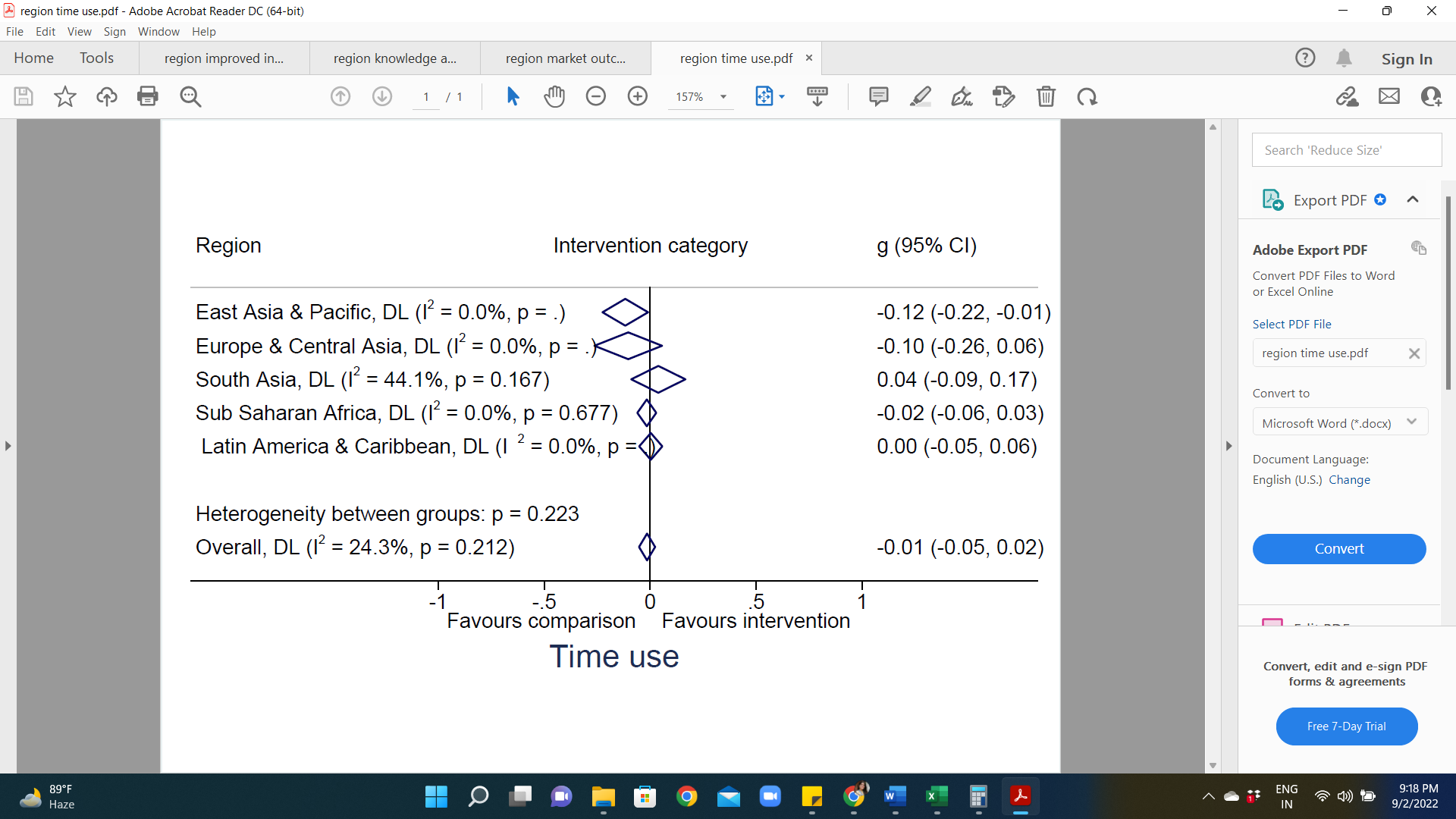


- - - 1. **Targeted /non-targeted interventions**


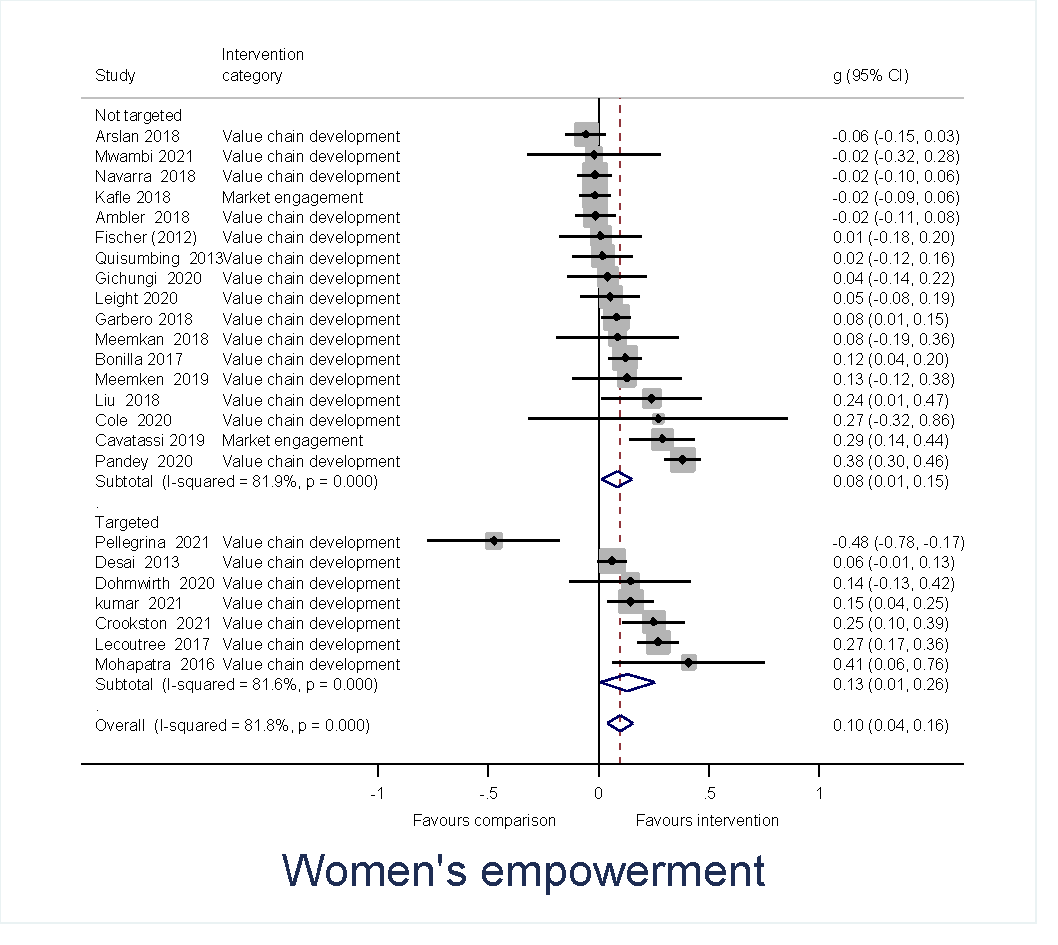


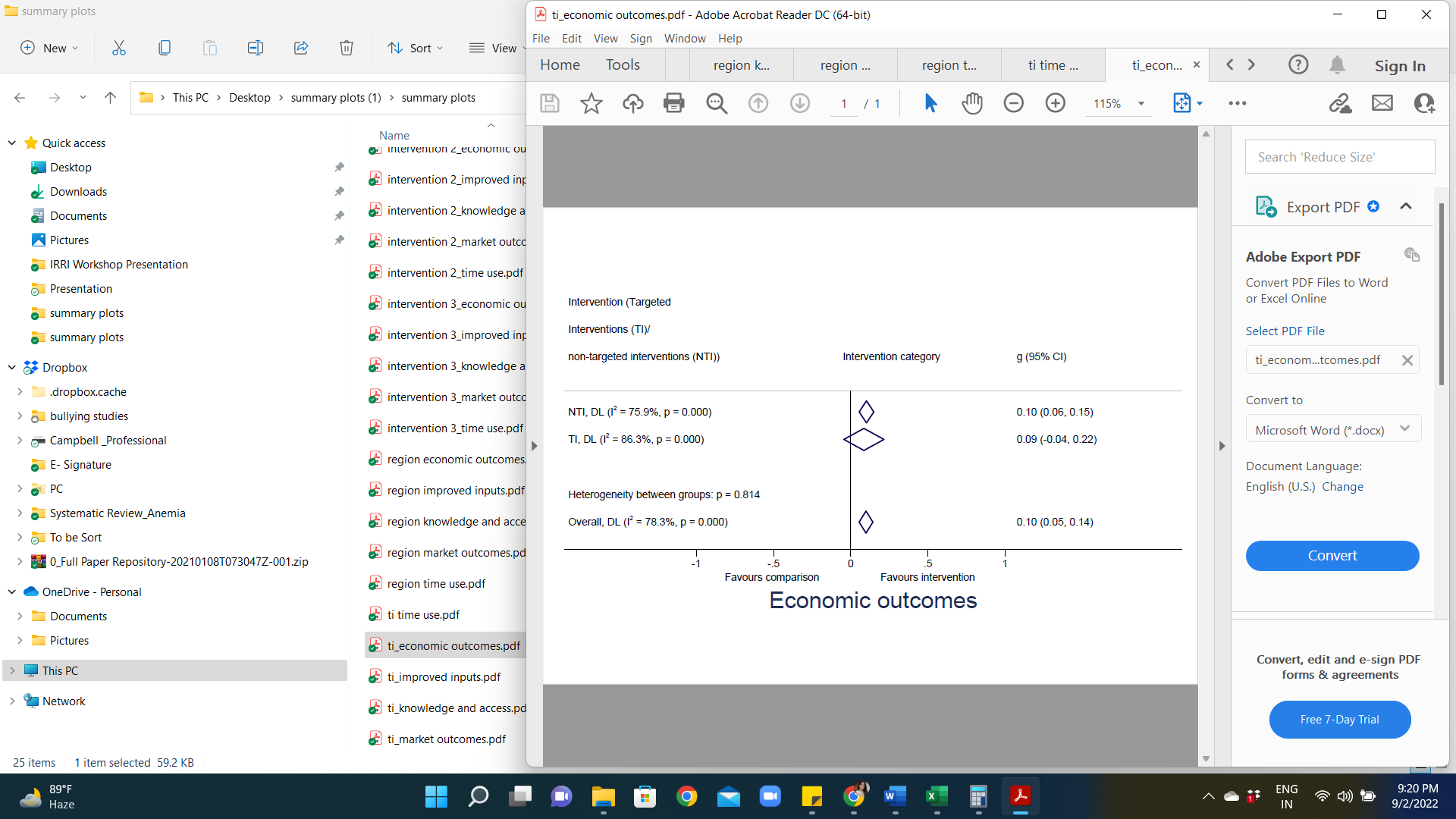


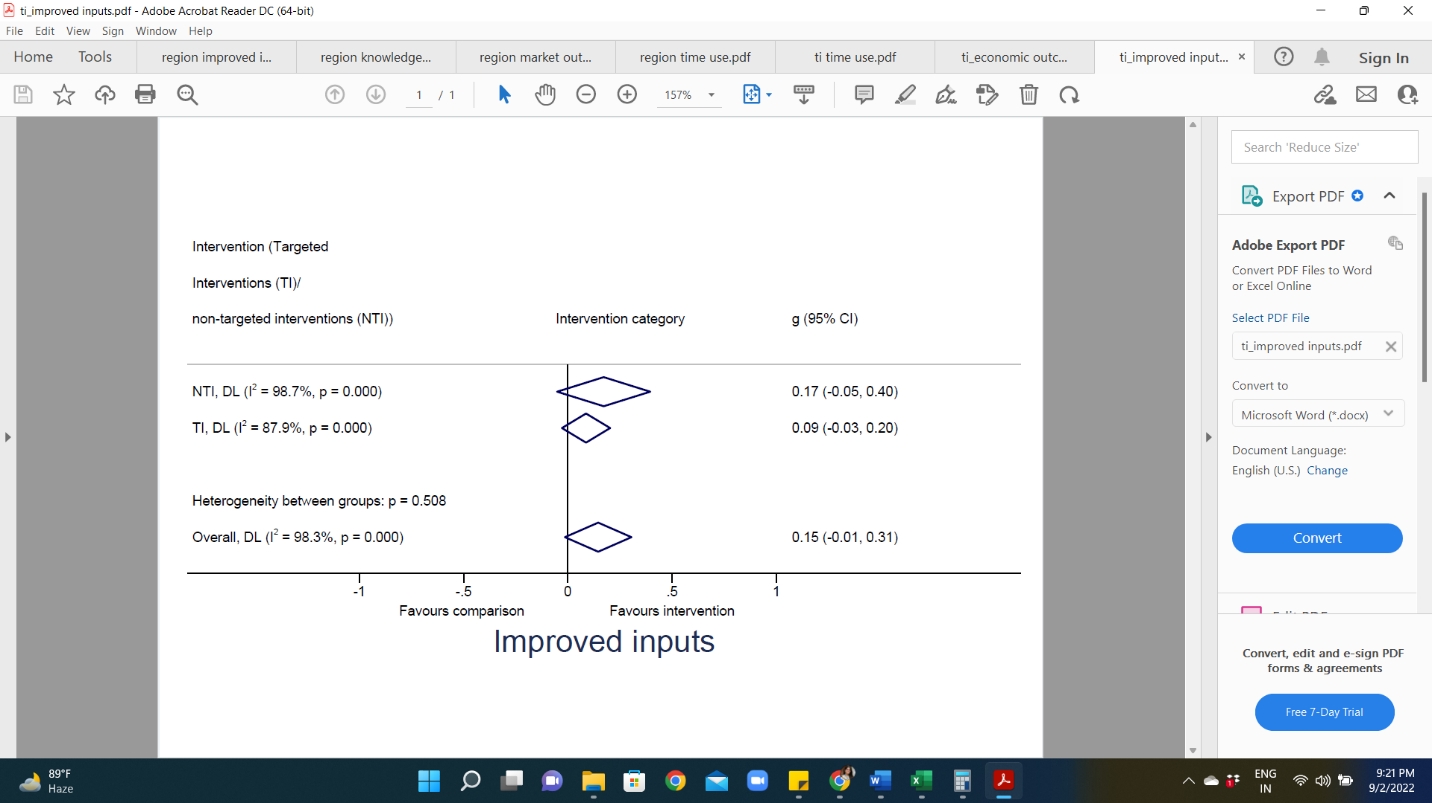


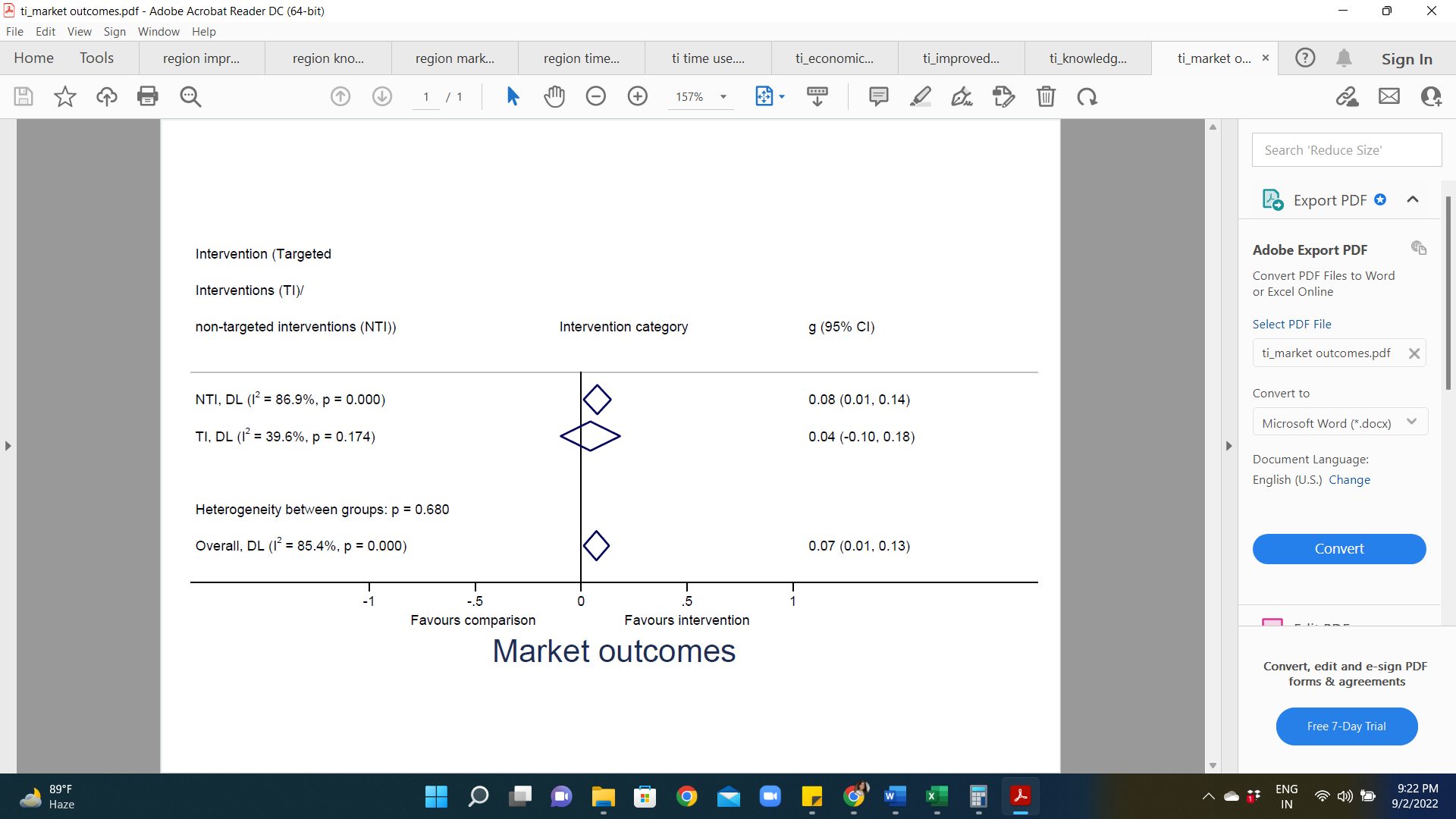


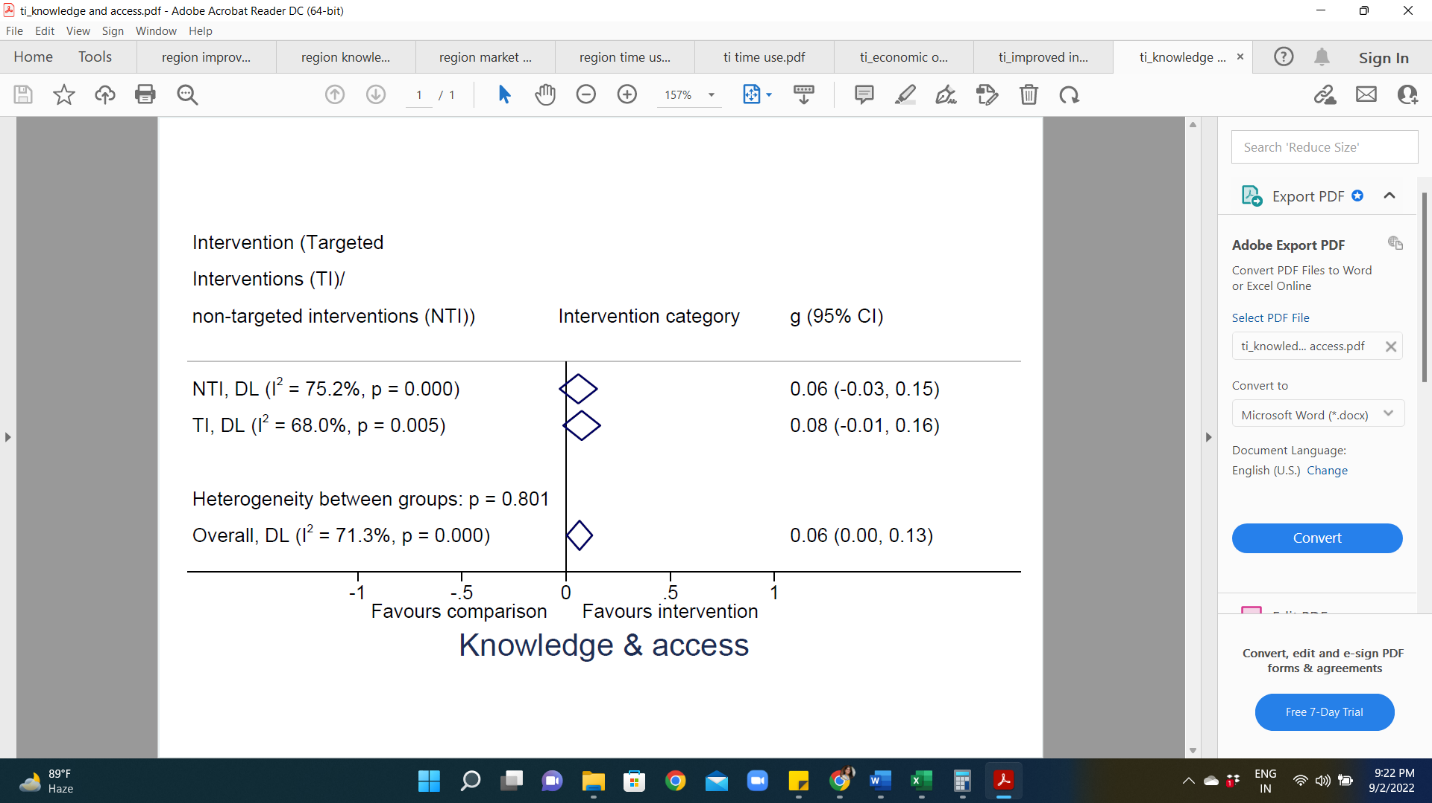


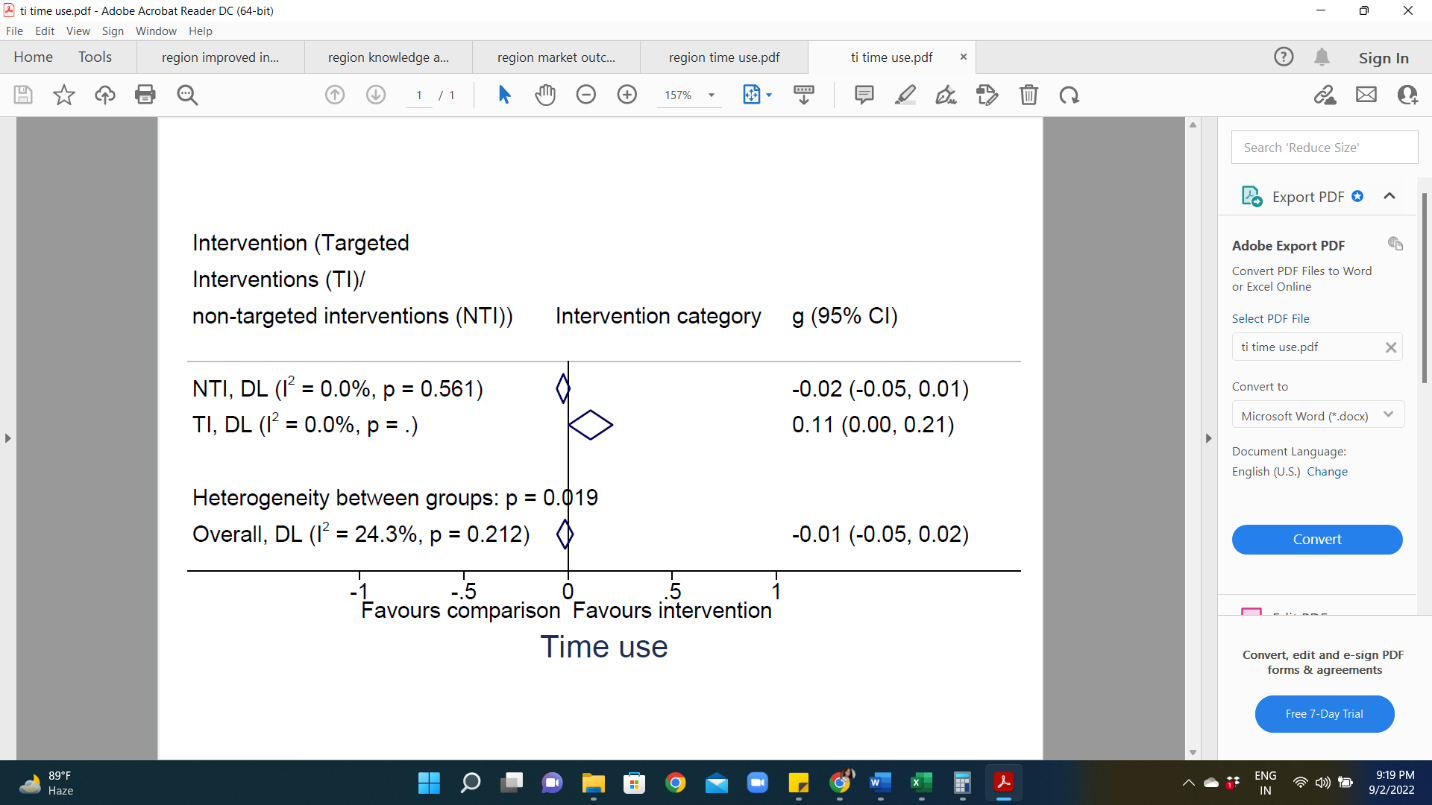


**3. Intervention categories**


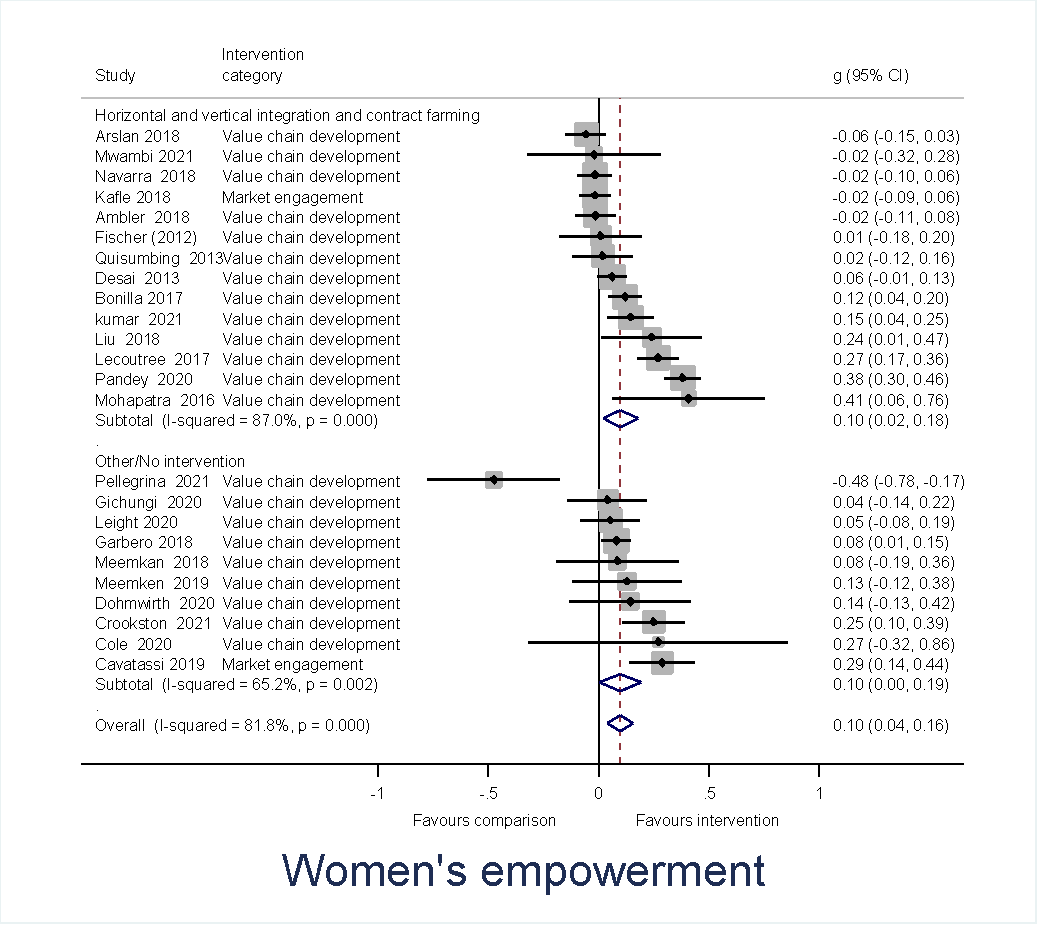


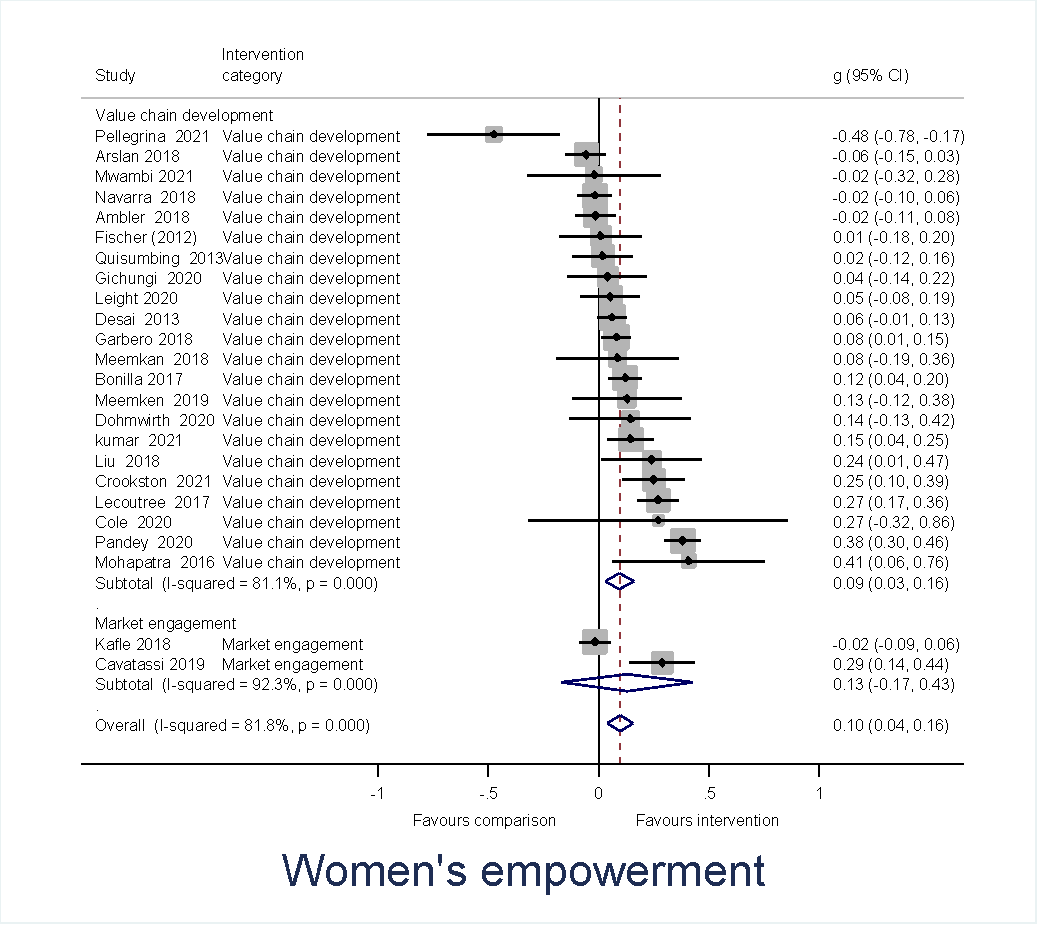

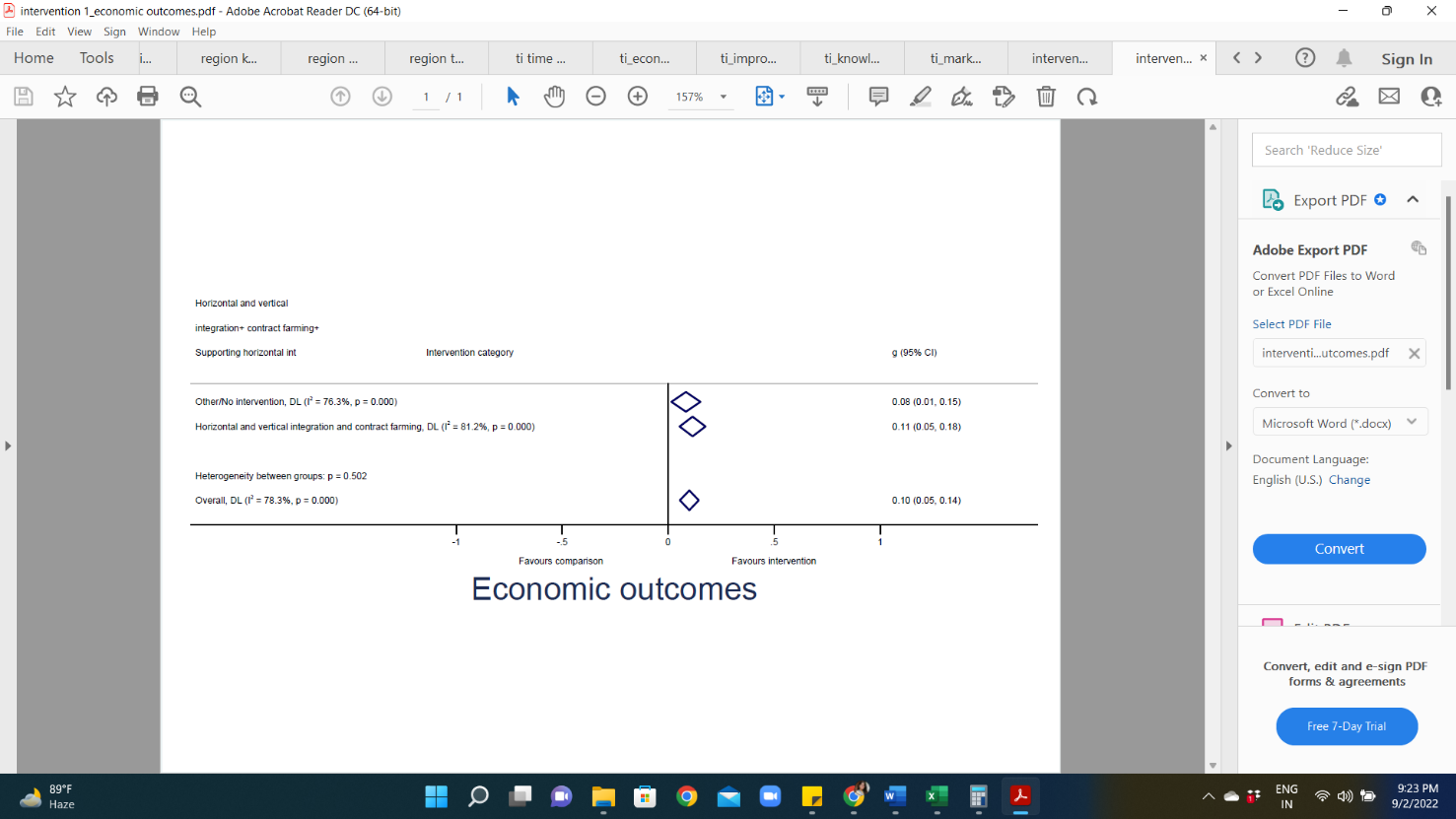


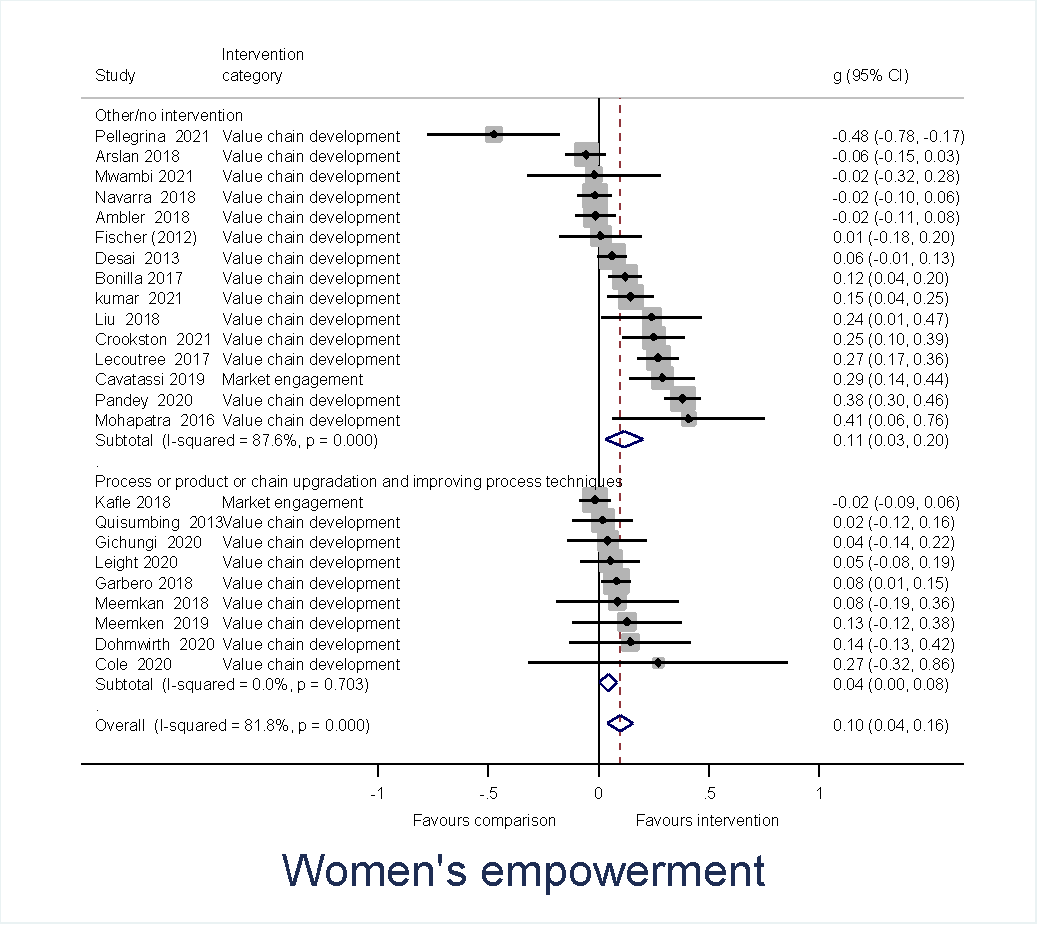

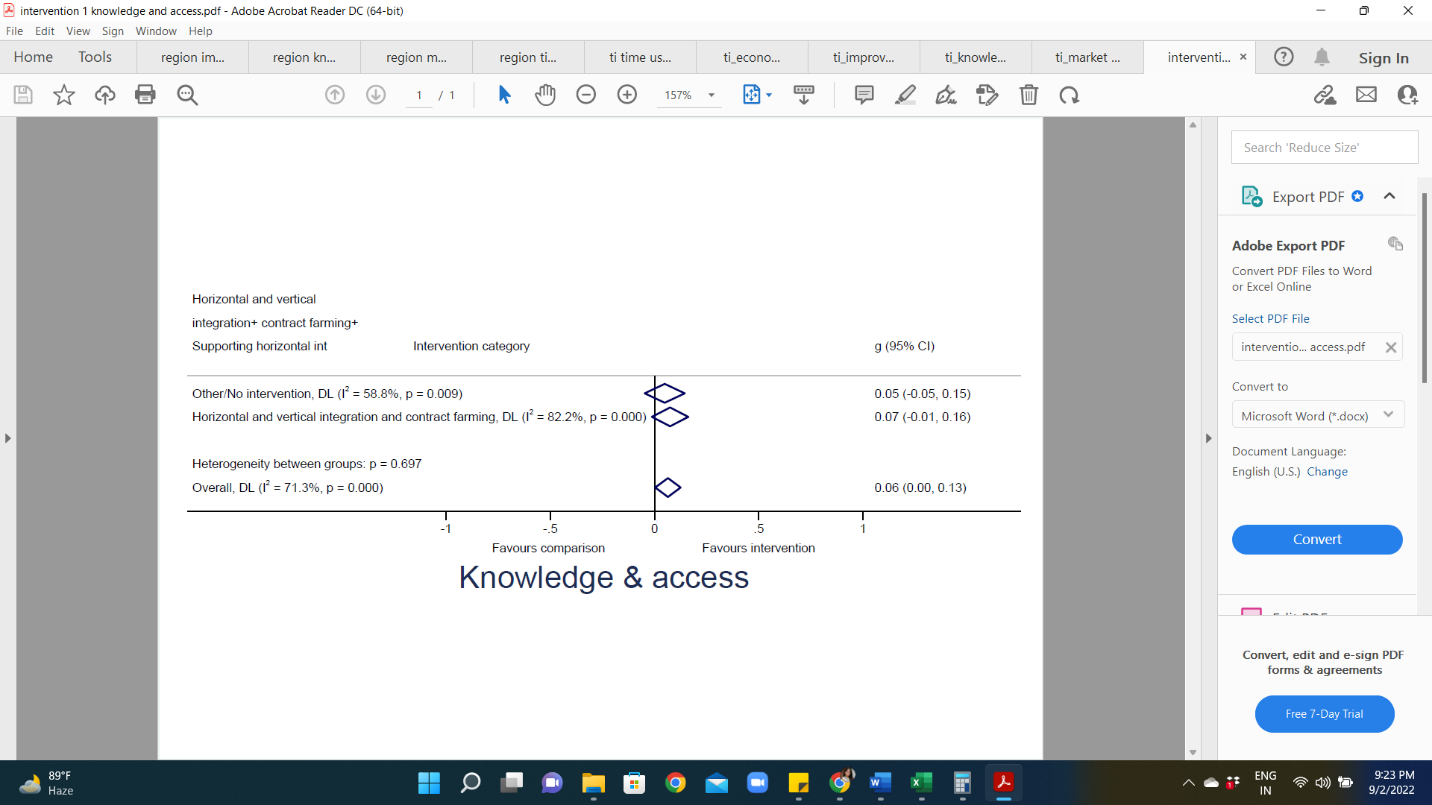


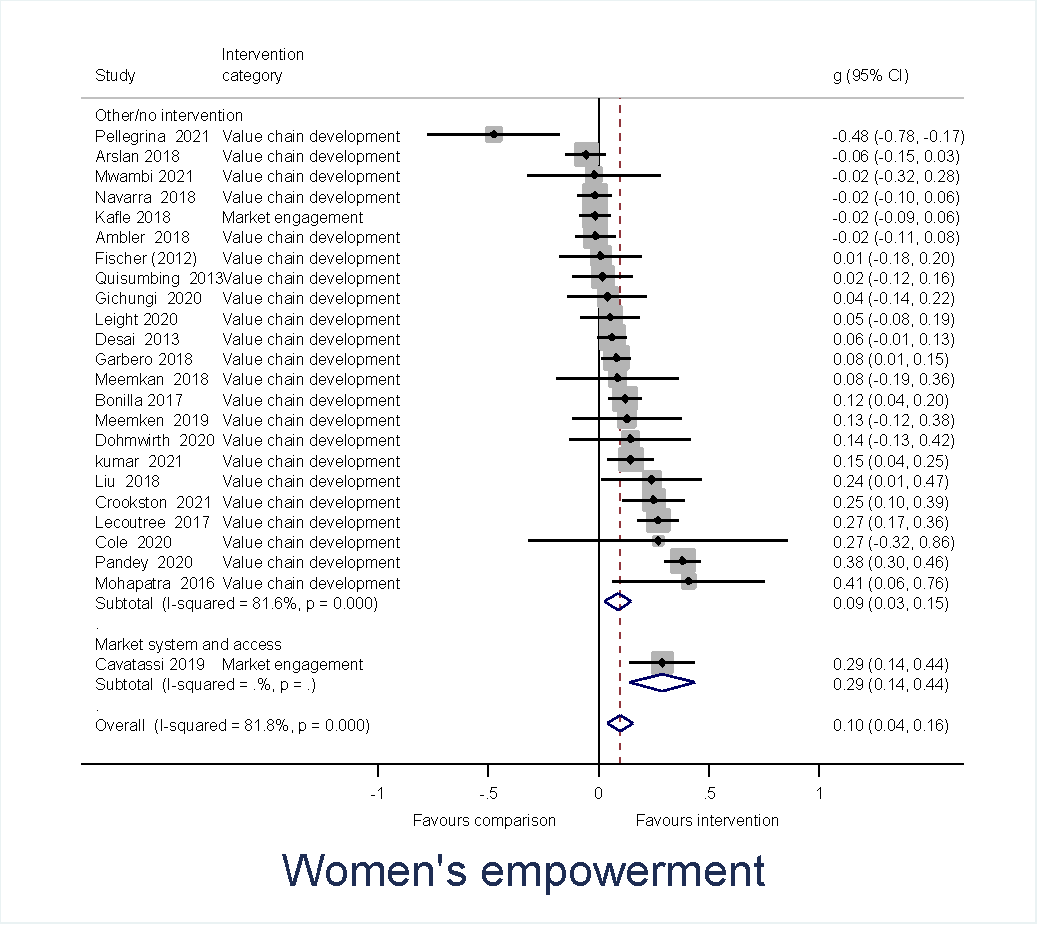


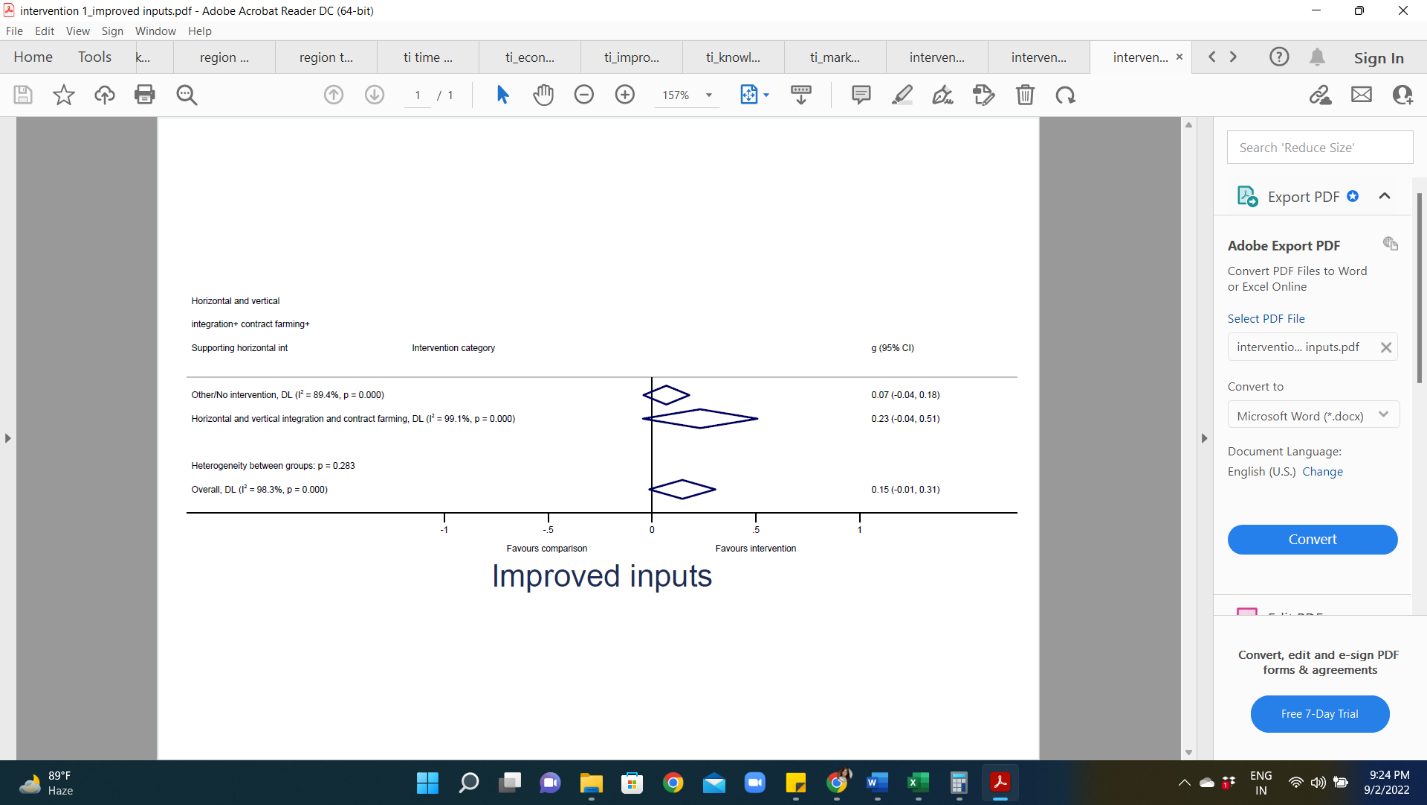


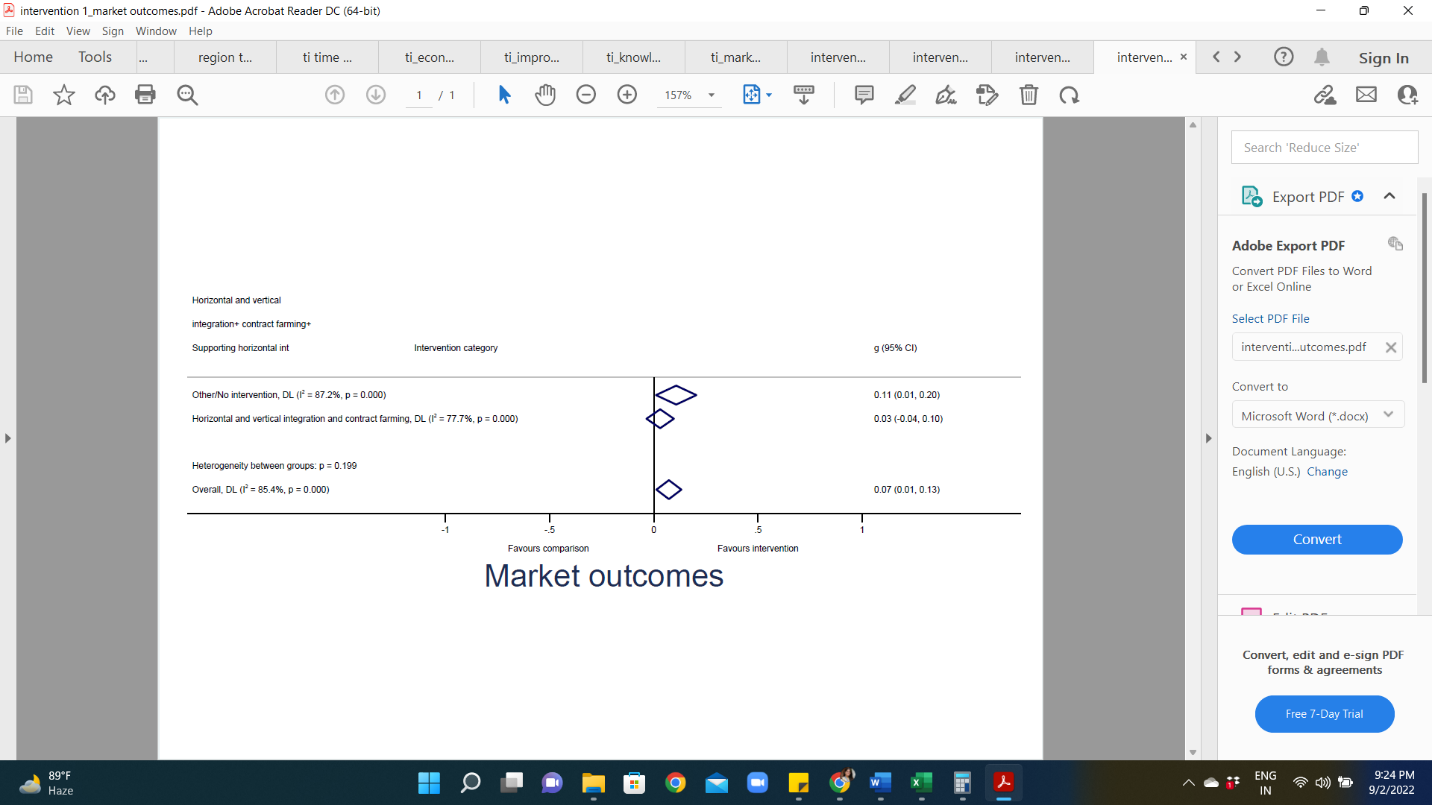

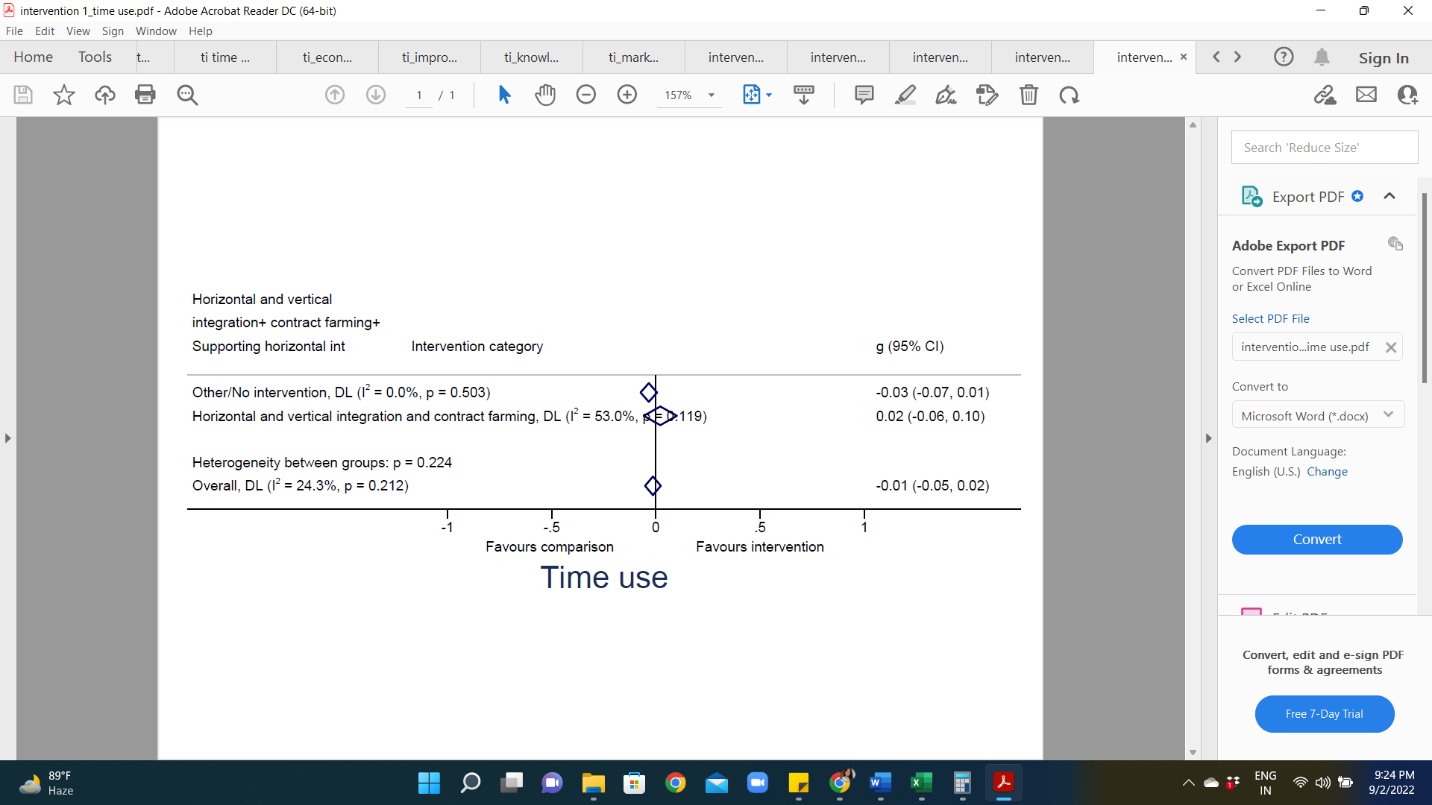

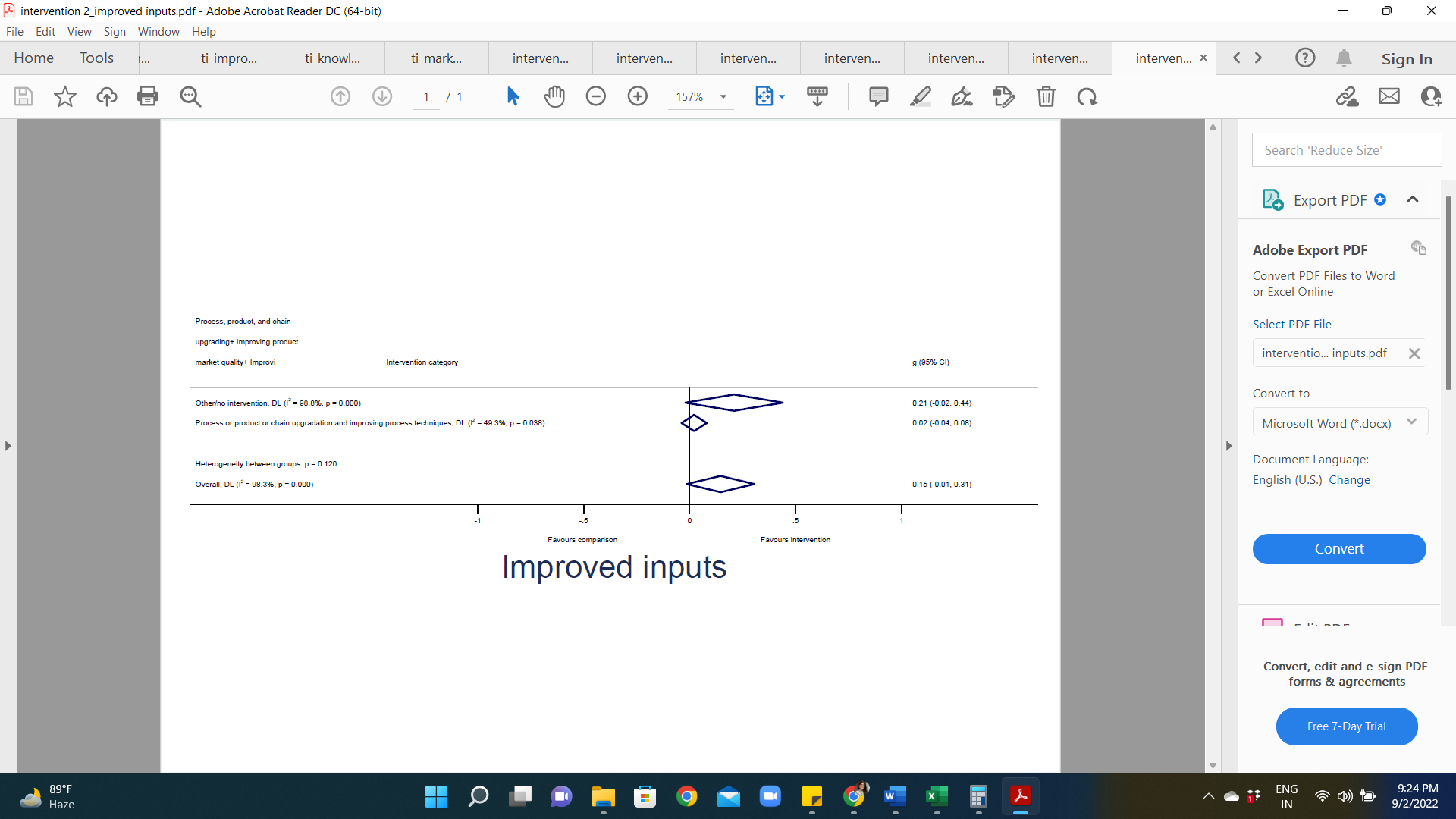


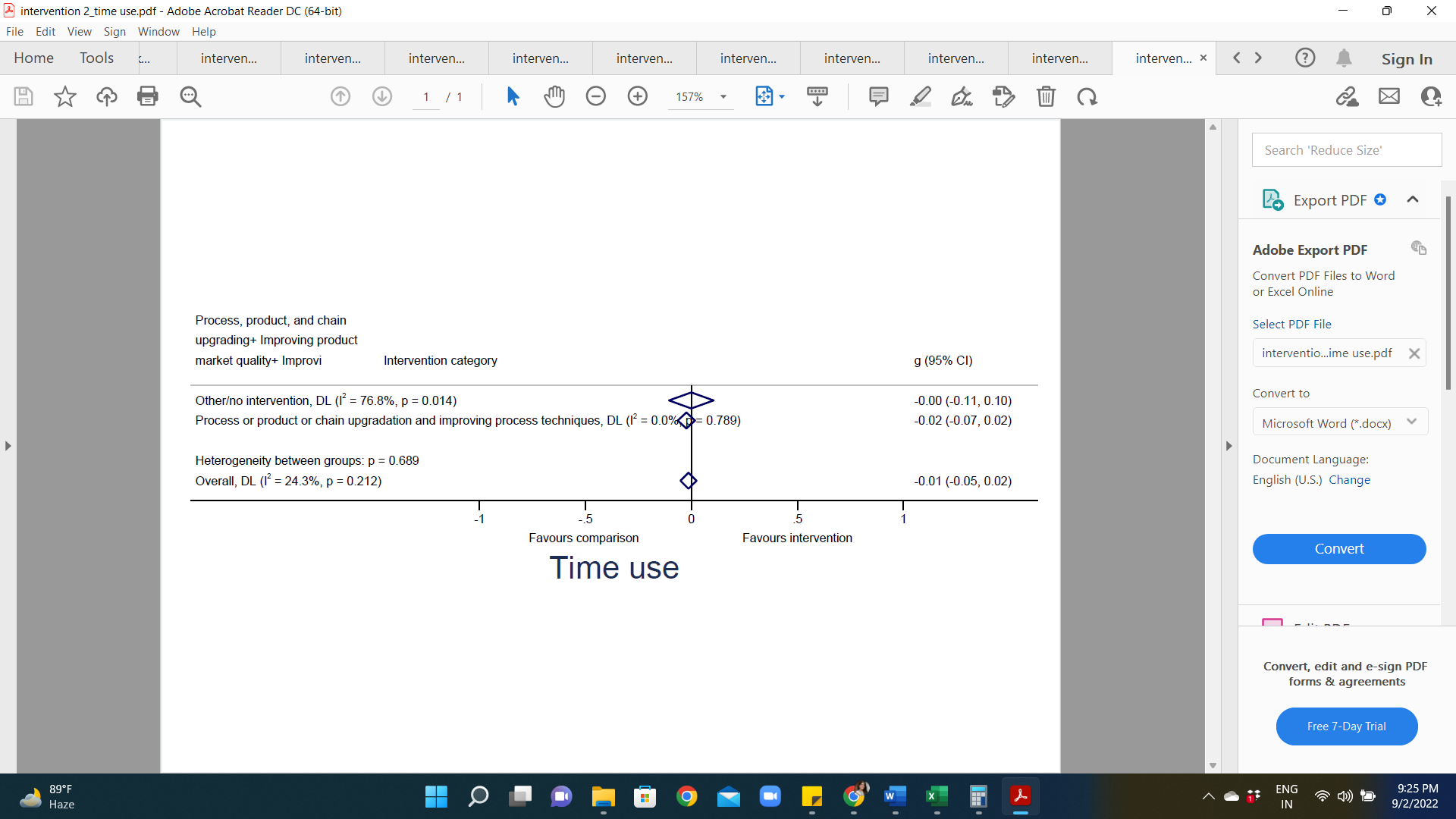

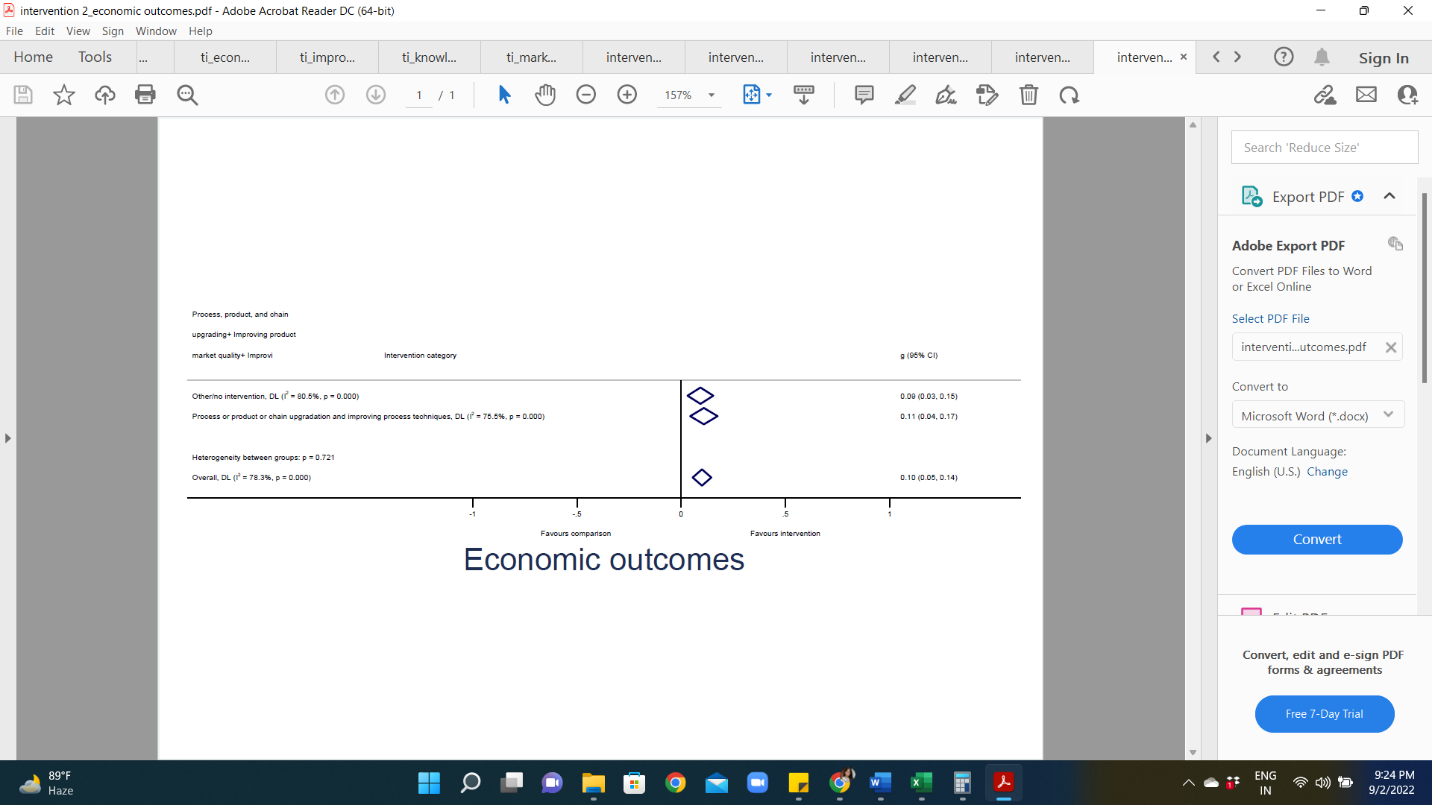

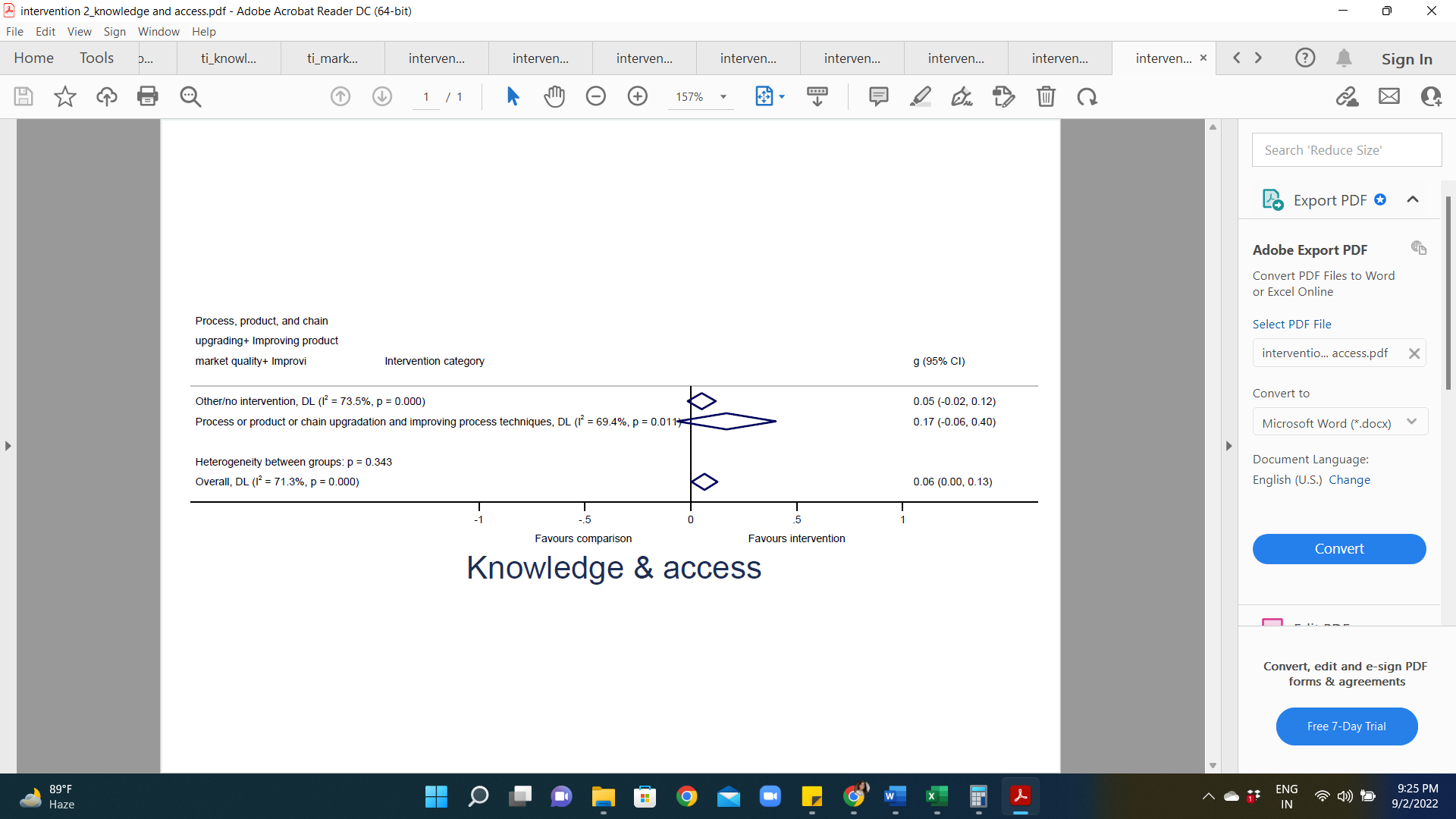

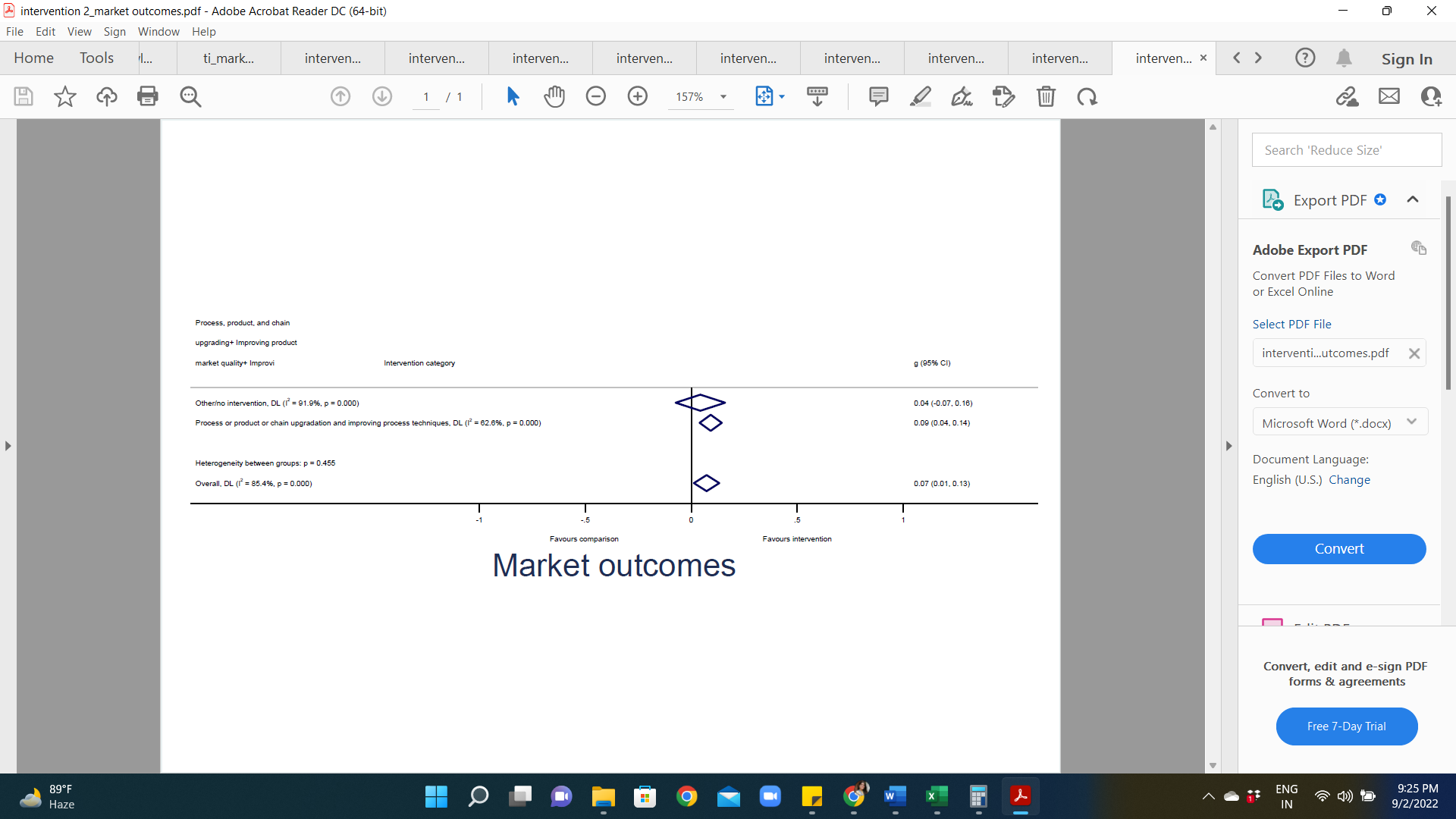


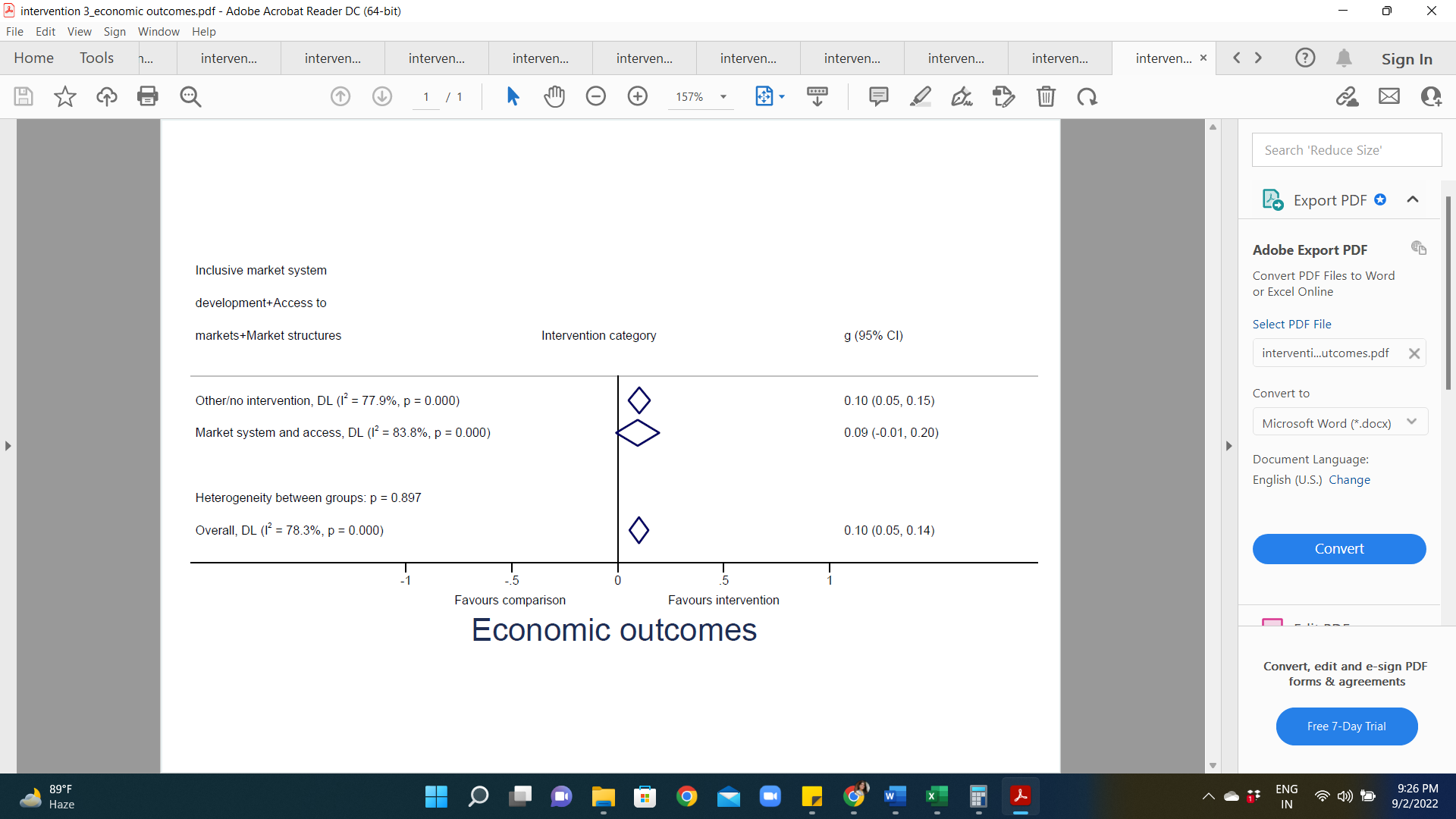

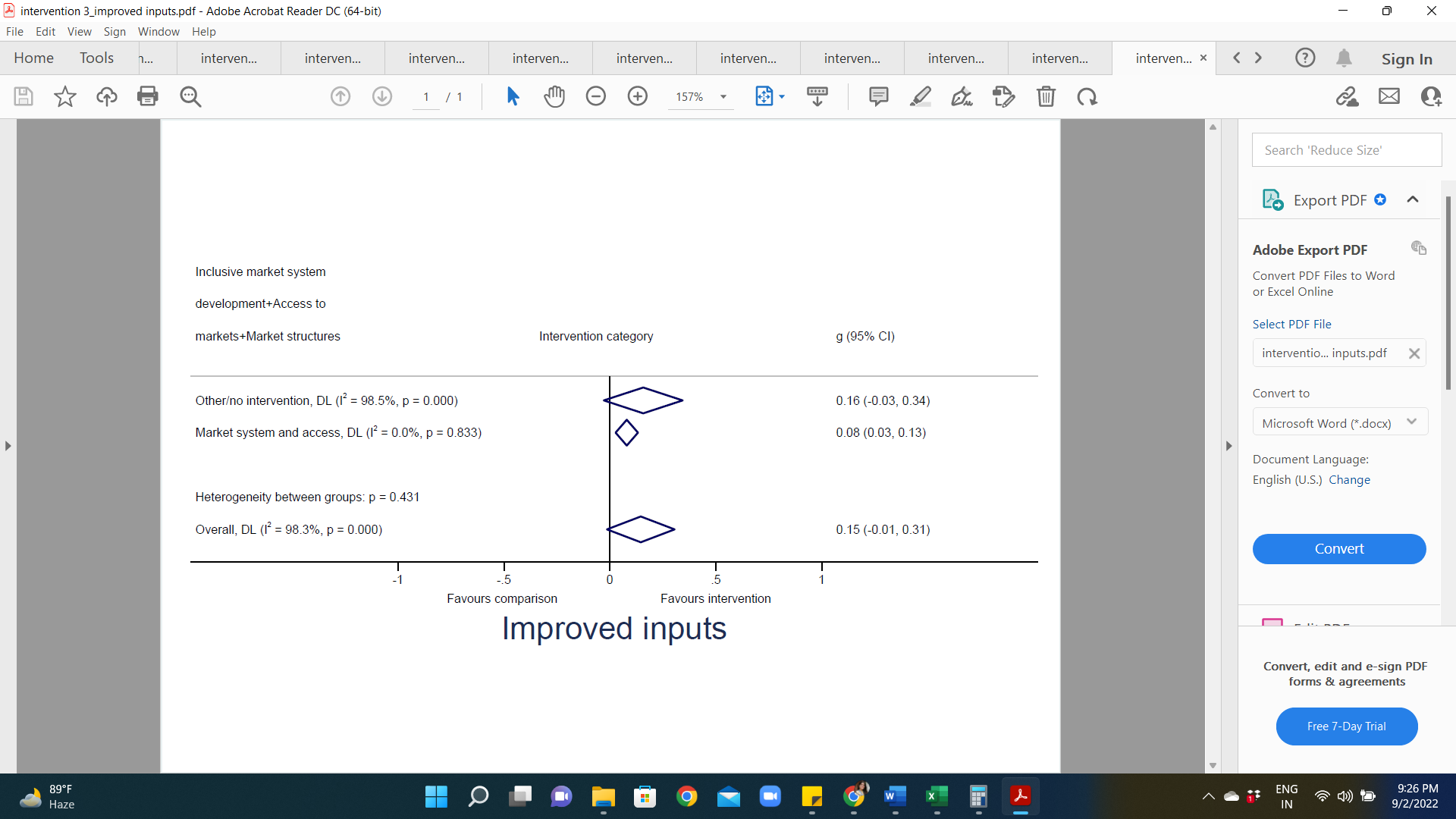

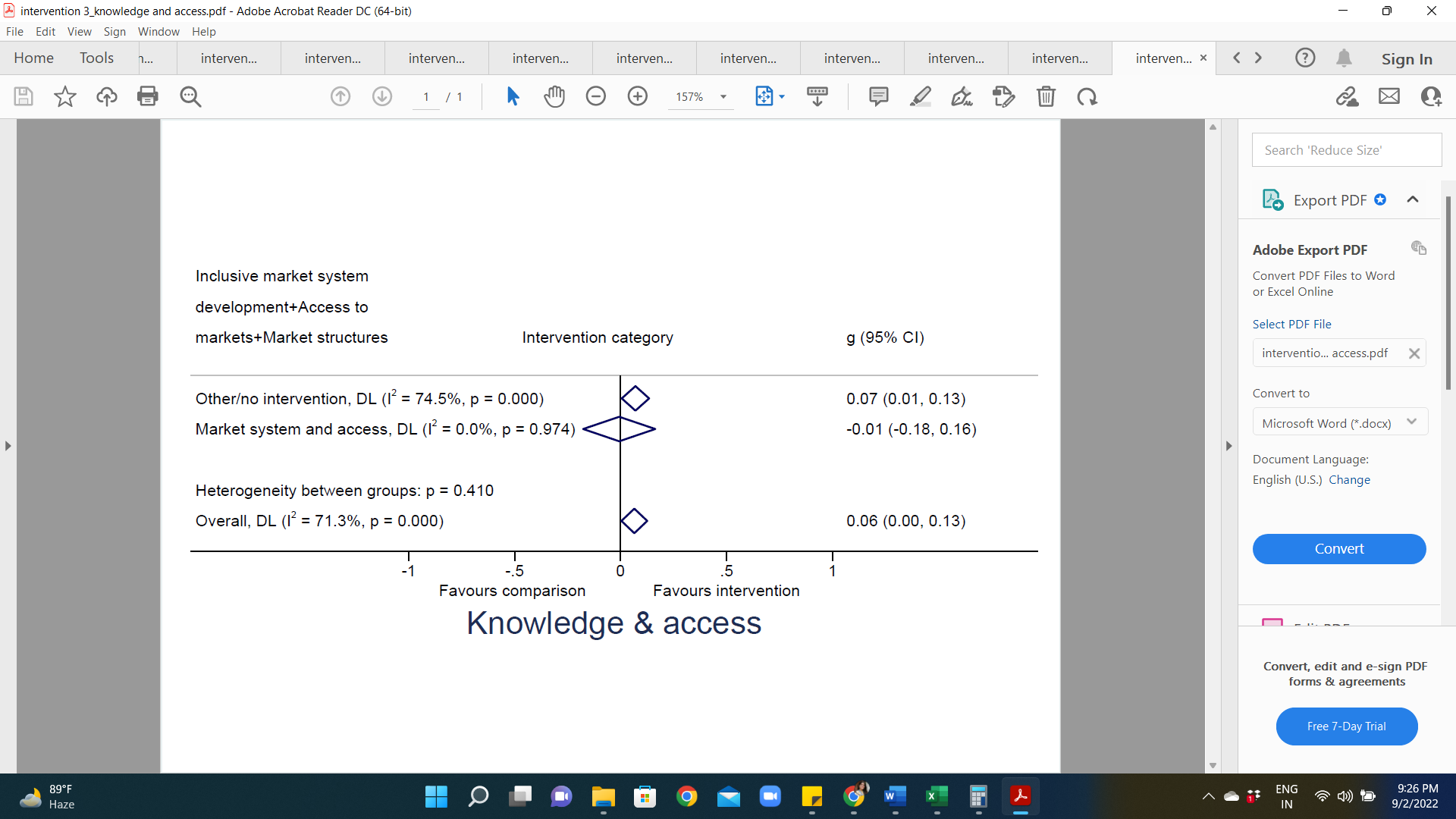

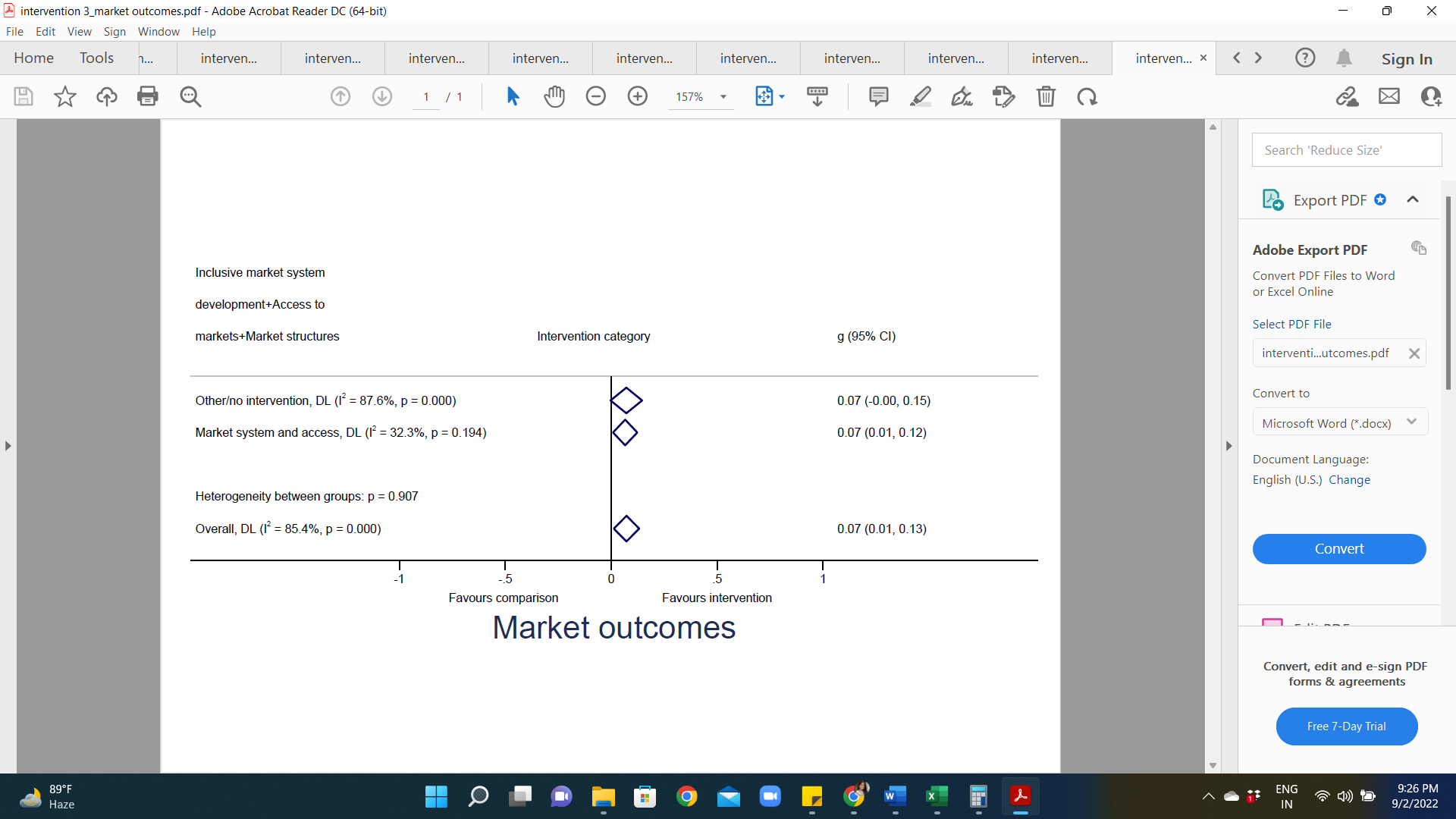

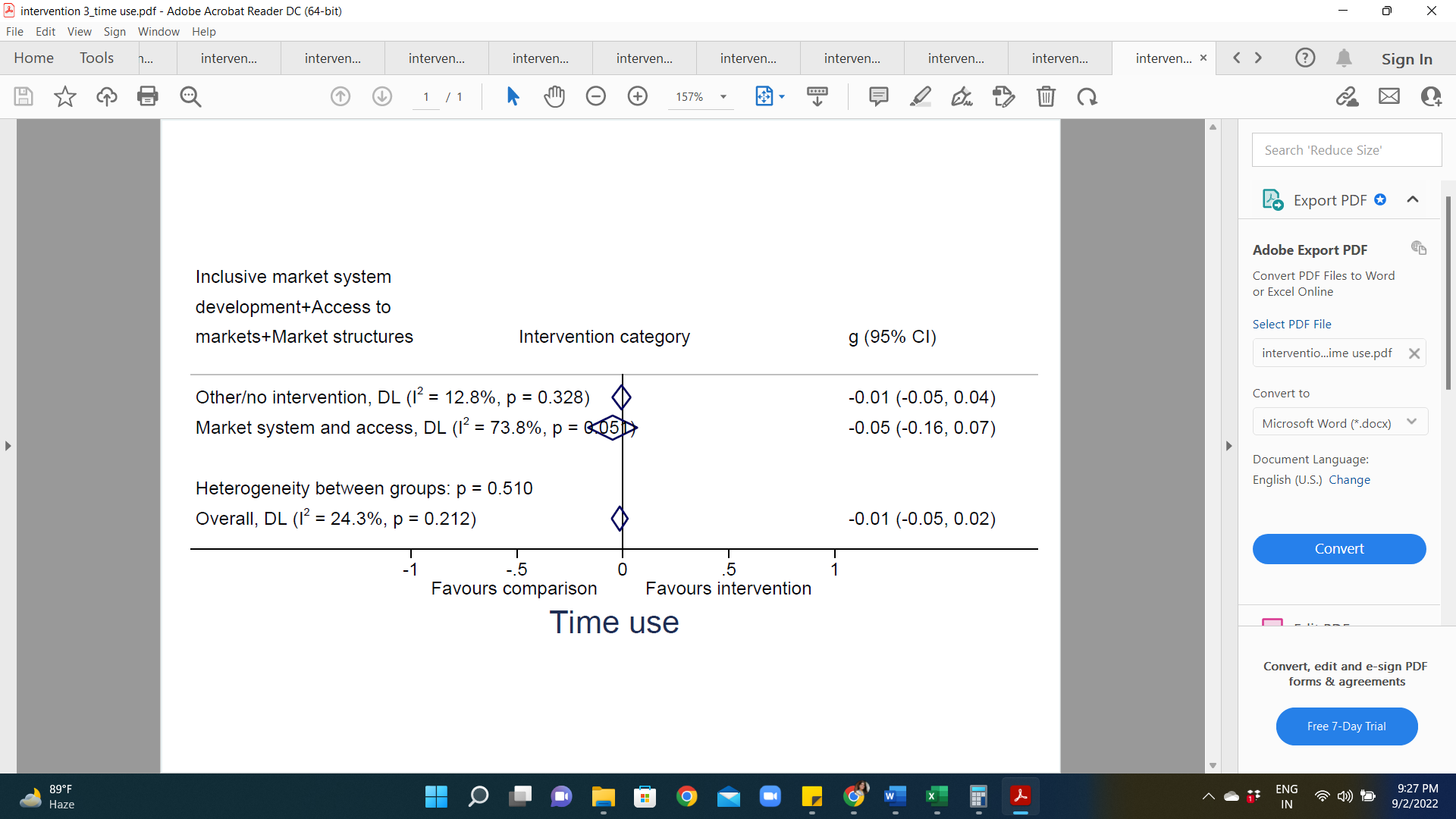


**Appendix G: List of excluded studies**

| **Author (Year)** | **Study title** | **Reason for exclusion** |
| --- | --- | --- |
| Bachmann (2012) | Potential and limitations of organic and fair trade cotton for improving livelihoods of smallholders: Evidence from Central Asia | Exclude on methodology and outcomes (No valid control group and No gender outcomes) |
| Ganesh et al. (2017) | Agricultural diversification in Nepal: Status, determinants, and its impact on rural poverty | Exclude on intervention and outcomes (No specific intervention and No gender outcomes) |
| Kikulwe et al. (2019) | Management of Banana Xanthomonas Wilt: Evidence from impact of adoption of cultural control practices in Uganda | Exclude on intervention (No VC intervention) |
| Kolapo et al. (2021) | Welfare and productivity impact of adoption of biofortified cassava by smallholder farmers in Nigeria | Exclude on intervention (No VC intervention) |
| Larson et al. (2015) | Are women less productive farmers? How markets and risk affect fertilizer use, productivity, and measured gender effects in Uganda | Exclude on intervention (No VC intervention) |
| Madheswaran & Dharmadhikary (2001) | Empowering rural women through self-help groups: lessons from Maharashtra Rural Credit Project | Exclude on intervention and methodology (No VC intervention and there is no control group) |
| Nikiema & Sakurai (2021) | Intrahousehold distribution of sales revenue and household nutritional outcomes: What if the wives controlled the farm revenue? | Exclude on intervention (No VC intervention) |
| Ojo et al. (2021) | Adoption of soil and water conservation technology and its effect on the productivity of smallholder rice farmers in Southwest Nigeria | Exclude on intervention (No VC intervention) |
| Resende et al. (2019) | Economic and productivity incentives to produce organically in Brazil: Evidence from strawberry production in the Federal District | Exclude on intervention and outcomes (No clear intervention and No gender outcomes) |
| van Rijn (2014) | Social capital, agricultural innovation and the evaluation of agricultural development initiatives | Exclude on methodology (Thesis outline) |
| Wang et al. (2021a) | Evaluating the impacts of smallholder farmer's participation in modern agricultural value chain tactics for facilitating poverty alleviation: A case study of kiwifruit industry in Shaanxi, China | Exclude on intervention (No specific intervention) |
| Wang et al. (2021b) | Does participation in agricultural value chain activities influence smallholder fruit grower production performance? A cross-sectional study of apple farmers in Shandong, China | Exclude on intervention (No specific intervention) |
| Zhao et al. (2021) | Empirical study on the effects of technology training on the forest-related income of rural poverty-stricken households - based on the PSM method | Exclude on outcomes (No gender outcomes) |
| Alejandro (2012) | The economic impact of improved bean varieties and determinants of market participation: Evidence from Latin America and Angola | Exclude based on study design (Regression Study design) |
| Ali (2015) | Investigating the gender gap in agricultural productivity: Evidence from Uganda | Exclude based on study design (Regression Study design) |
| Ali (2020) | Gender and impact of climate change adaptation on soybean farmers' revenue in rural Togo, West Africa | Exclude on intervention |
| AO (2021) | Organization model, vertical integration, and farmers’ income growth: Empirical evidence from large-scale farmers in Lin’an, China | Exclude on methodology (Simulation model study) |
| Arndt | Agricultural technology, risk, and gender: A CGE analysis of Mozambique | Exclude on methodology (Simulation model study) |
| Aryal (2020) | Adoption and economic impacts of laser land levelling in the irrigated rice-wheat system in Haryana, India using endogenous switching regression | Exclude on intervention |
| Asante (2017) | Integrated crop-livestock management practices, technical efficiency and technology ratios in extensive small-ruminant systems in Ghana | Exclude on intervention |
| Awotide (2016) | Agricultural technology adoption, commercialization and smallholder rice farmers' welfare in rural Nigeria | Exclude on intervention |
| Bannor (2021) | Entrepreneurial behaviour among non-timber forest product-growing farmers in Ghana: An analysis in support of a reforestation policy | Exclude on methodology (Regression study) |
| Barah (2010) | System of rice intensification (SRI): economic and ecological benefits of improved production practice for food security and resource conservation | Exclude on intervention |
| Chiputwa (2016) | Sustainability standards, gender, and nutrition among smallholder farmers in Uganda | Exclude on outcome |
| de Andrade (2021) | Gender and small-scale fisheries in Brazil: Insights for a sustainable development agenda | Exclude on methodology (Regression study) |
| de Brauw & Bulte (2021) | Migration, labor and women's empowerment: Evidence from an agricultural value chain in Bangladesh | Exclude on methodology (Regression study) |
| Dietz (2018) | Women's empowerment in rural Honduras and its determinants: Insights from coffee communities in Ocotepeque and Copan | Exclude |
| Dissanayake (2014) | Empowerment of women through self-reliance approach in the rice processing village programme | Exclude on methodology |
| Essien (2014) | An analysis of access to credit markets and the performance of small scale agro-based enterprises in the Niger Delta Region of Nigeria | Exclude on methodology (Regression study) |
| Fabry (2022) | Decent work in global food value chains: Evidence from Senegal | Exclude on methodology (Regression study) |
| Gathorne-Hardy (2016) | System of rice intensification provides environmental and economic gains but at the expense of social sustainability: A multidisciplinary analysis in India | Exclude on methodology |
| Gbetnkom (2007) | Forest management, gender, and food security of the rural poor in Africa | Exclude on methodology (Regression study) |
| Genereuse (2012) | Analysis of factors influencing women participation in coffee value chain in Huye District, Rwanda | Exclude on methodology (Regression study) |
| Kuwornu (2013) | Global GAP standard compliance and smallholder pineapple farmers' access to export markets: Implications for incomes | Exclude on methodology |
| Kwapong (2021) | Determinants of scale of farm operation in the eastern region of Ghana | Exclude on methodology (Regression study) |
| Lin (2021) | Farmer social networks: The role of advice ties and organizational leadership in agroforestry adoption | Exclude on methodology (Regression study) |
| Liu (2019) | Technical training and rice farmers' adoption of low-carbon management practices: The case of soil testing and formulated fertilization technologies in Hubei, China | Exclude on intervention |
| Makinya (2021) | The importance of store hygiene for reducing post-harvest losses in smallholder farmers' stores: Evidence from a maize-based farming system in Kenya | Exclude on methodology (Regression study) |
| Martey (2014) | Market information and extent of agricultural commercialization: Empirical evidence from smallholder farmers in Effutu Municipality of Ghana | Exclude on methodology (Regression study) |
| Mehnaz (2021) | Women farmers' access to and control over farming resources and their role in decision making process in the rural areas of Khyber Pakhtunkhwa, Pakistan | Exclude on methodology (Associational study) |
| Menasbo (2020) | Does fertilizer adoption enhance smallholders' commercialization? An endogenous switching regression model from northern Ethiopia | Exclude on methodology (Regression study) |
| Gondwe (2017) | Correlates and consequences of women's participation in the cowpea value chain in Eastern Zambia | Exclude on methodology (Regression study) |
| Grillos (2018) | Women's participation in environmental decision-making: Quasi-experimental evidence from northern Kenya | Exclude on intervention |
| Hanjra (2011) | The political economy of maize production and poverty reduction in Zambia: Analysis of the last 50 years | Exclude on methodology (Regression study) |
| Hichaambwa (2015) | Determinants and welfare effects of smallholder participation in horticultural markets in Zambia | Exclude on methodology (Regression study) |
| Imai (2014) | Agricultural employment, wages and poverty in developing countries | Exclude on methodology (model) |
| Ingutia (2022) | Do farmer groups improve the situation of women in agriculture in rural Kenya? | Exclude on methodology (Regression study) |
| Jalal (2015) | Food insecurity mediates the effect of a poverty-alleviation programme on psychosocial health among the ultra-poor in Bangladesh | Exclude on intervention |
| Kassie (2017) | Agroforestry and farm income diversification: synergy or trade-off? The case of Ethiopia | Exclude on methodology (Regression study) |
| Khandker (1996) | The Bangladesh Rural Advancement Committee's credit programmes | Exclude on methodology |
| Koide (2021) | Viability of smallholder dairy cattle management and its intensification strategies based on whole-farm analyses in southern Mozambique | Exclude on methodology |
| Rifai (2019) | Advancing rural microfinance through local government: Social commitment and financial sustainability in riau province of Indonesia | Exclude on methodology |
| Kumar (2015) | Rural labour employment and their livelihoods in Bihar: Micro-level evidences from selected villages | Exclude on intervention |
| Kundu (2022) | Occupational diversification as livelihood strategy among the agricultural labour households of West Bengal, India | Exclude on intervention and methodology (Regression) |
| Ng'atigwa (2020) | Assessment of factors influencing youth involvement in horticulture agribusiness in Tanzania: A case study of Njombe region | Exclude on methodology and outcome (Regression study design) |
| Niu (2021) | Sustainable intensification of cultivated land use and its influencing factors at the farming household scale: A case study of Shandong Province, China | Exclude on methodology (Regression study) |
| Ochieng (2010) | What is the effect of management interventions package on productivity of indigenous chicken in Western Kenya? | Exclude on methodology (Regression study) |
| Odurukwe (2003) | Intra-household impacts of small farm commercialization of maize enterprise in Abia State, Nigeria | Exclude on methodology (Regression study) |
| Olaleye (2009) | Effect of dry season tomato farming on poverty alleviation among women farmers in Niger State, Nigeria | Exclude on methodology (Regression study) |
| Omeje (2020) | Participation of stakeholders in aquaculture value chain of the West African Agricultural Productivity Programme in Nigeria | Exclude on methodology (Regression study) |
| Opata (2020) | Impact of women's share of income on selected value chains expenditure in rural south-east Nigeria | Exclude on methodology (Regression study) |
| Põldaru (2018) | Optimization of arable land use to guarantee food security in Estonia | Exclude on outcome |
| Rahman (2007) | Adoption of commercial broiler and layer packages of Bangladesh: a means of empowering women for rural development | Exclude on methodology |
| Rahman (2019) | Women's gainful employment in 'gher' farming system (prawn-carp-rice integrated culture) in Bangladesh: Trends and determinants | Exclude on methodology (Regression study) |
| Rakhshanda (2018) | Secured land rights, household welfare and agricultural productivity: Evidence from rural Pakistan | Exclude on intervention |
| Rana (2007) | Influence of socio-economic and cultural factors in rice varietal diversity management on-farm in Nepal | Exclude on methodology (Regression study) |
| Reetsch (2020) | Organic farm waste management in degraded banana-coffee-based farming systems in NW Tanzania | Exclude on outcome |
| Regassa (2021) | Incentivising and retaining public servants in remote areas: A discrete choice experiment with agricultural extension agents in Ethiopia | Exclude on outcome |
| Regmi (2016) | Impact of remittance on food security in Bangladesh | Exclude on outcome |
| Rehan (2017) | Determinants of on-farm diversification in Bangladesh | Exclude on methodology (Regression study) |
| Reimer (2014) | Are modern varieties always better? An economic analysis of maize varietal selection | Exclude on methodology (Regression study) |
| Rekha (2019) | Achieving food security in India: Need for an integrated approach | Exclude on outcome |
| Rekik (2020) | Understanding soil health and associated farmers' perceptions in Colombian coffee systems | Exclude on methodology (Regression study) |

*Note: VC = value chain.*
